# Identification of core competencies for exercise oncology professionals: A Delphi study of United States and Australian participants

**DOI:** 10.1002/cam4.70004

**Published:** 2024-07-24

**Authors:** Mary A. Kennedy, Kelley Covington Wood, Anna Campbell, Melanie Potiaumpai, Christopher M. Wilson, Anna L. Schwartz, Jessica Gorzelitz, Maxime Caru, Kathryn H. Schmitz

**Affiliations:** ^1^ Nutrition and Health Innovation Research Institute, School of Medical and Health Sciences Edith Cowan University Joondalup Western Australia Australia; ^2^ ReVital Cancer Rehabilitation, Select Medical Mechanicsburg Pennsylvania USA; ^3^ School of Applied Sciences Edinburgh Napier University Edinburgh UK; ^4^ Division of Hematology and Oncology University of Pittsburgh Pittsburgh Pennsylvania USA; ^5^ Physical Therapy Program, School of Health Sciences Oakland University Rochester Michigan USA; ^6^ Founding Residency Program Director Beaumont Health Oncology Residency Troy Michigan USA; ^7^ College of Nursing, University of Nebraska Medical Center Omaha Nebraska USA; ^8^ Department of Health and Human Physiology University of Iowa Iowa City Iowa USA; ^9^ Department of Pediatrics, Division of Hematology and Oncology Pennsylvania State Health Children's Hospital Hershey Pennsylvania USA

**Keywords:** competency, Delphi, exercise, oncology, professional development

## Abstract

**Introduction:**

Integration of exercise into standard oncology care requires a highly skilled workforce of exercise professionals; however, competency requirements have not kept pace with advancements in the field. Therefore, the aim of this study was to obtain consensus on core competencies required for an exercise professional to be qualified to work with adults undergoing active cancer treatment.

**Materials and Methods:**

A three‐round modified electronic Delphi process was used. In Round 1, an international group of 64 exercise oncology stakeholders (i.e., exercise oncology professionals (*n* = 29), clinical referrers (*n* = 21), and people with lived experience (*n* = 14)) responded to open‐ended prompts eliciting perspectives regarding competencies needed for an exercise oncology professional to work with adults receiving active cancer treatment. Subsequently, only exercise oncology professionals participated, ranking the importance of competencies. In Round 2, professionals received summary feedback, ranked new competencies generated from open‐ended responses, and reranked competencies not reaching consensus. In the final round, professionals finalized consensus ranking and rated frequency and mastery level for each.

**Results:**

Consensus was reached on 103 core competencies required for exercise professionals to be qualified to deliver care to adults undergoing active cancer treatment. The core competencies represent 10 content areas and reflect the needs of clinical referrers and people with lived experience of receiving cancer treatment.

**Conclusions:**

The core competencies identified reflect significant advancements in the field of exercise oncology. Results will underpin the development of education, certification, and employment requirements for exercise oncology professionals, providing a critical step toward achieving routine integration of exercise into standard oncology care.

## Graphical abstract

Integration of exercise into standard oncology care requires a highly skilled workforce of exercise professionals; however, competency requirements have not kept pace with advancements in the field. An expert consensus process was used to identify core competency requirements of exercise oncology professionals working with people undergoing active cancer treatment to underpin the development of their future education, certification, and employment requirements.

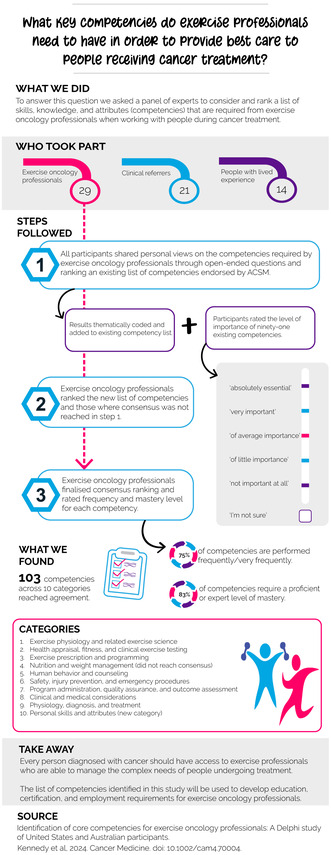

## 1 INTRODUCTION

Calls to integrate exercise into standard oncology care are being led by the United States (US) and Australia^1^, ^2^, ^3^ to ensure all people living with and beyond cancer can access this evidence‐based intervention shown to help manage multiple health‐related side effects of cancer treatment.^4^, ^5^ In the US, a 2018 Roundtable convened by the American College of Sports Medicine (ACSM) called for clinicians to incorporate exercise assessment, advice, and referrals as standard practice for people with cancer.^1^ This call for integration was supported by a 2022 guideline from the American Society of Clinical Oncology (ASCO) stating oncology providers should recommend exercise during active treatment with curative intent.^2^ In parallel, the Clinical Oncology Society of Australia (COSA) issued a position statement in 2018 calling on all healthcare professionals involved in the care of people with cancer to embed exercise as a standard component of cancer care.^3^

Despite these calls to action, exercise is not effectively translated into clinical practice: Data consistently suggest fewer than 15% of people diagnosed with cancer receive a referral to exercise during cancer treatment.^6^, ^7^, ^8^ The Moving Through Cancer (MTC) task force, a multidisciplinary group of exercise oncology experts, summarized the complex issues underpinning this poor translation into five strategic priority areas to be addressed to achieve routine integration of exercise in cancer care by 2029.^9^ One identified priority was the need for a well‐developed exercise workforce sufficiently competent in oncology.

Identifying exercise professionals competent in oncology is difficult for several reasons. University degrees, required for many roles, vary widely in their content, and specific training in oncology is minimal at best. Consequently, many exercise professionals seek additional oncology training, and many people look to ACSM for this training as the organization is the internationally recognized leader of exercise promotion and certification. There are two exercise oncology certifications that have been developed by or endorsed by ACSM (i.e., ACSM's Cancer Exercise Trainer (CET) certification & CanRehab); however, they were developed in 2008 and 2006, respectively and their supporting competencies have not been reviewed since inception. Therefore, these exemplar certifications are not reflective of the significant advancements made in the field over the past 15 years.^4^, ^5^, ^10^ Further, more than 20 exercise oncology certifications have emerged since ACSM's CET and CanRehab certifications were developed, and these newer certifications vary widely in content, delivery methods, and requirements. Additionally, many professionals gain skills on‐the‐job as real‐world opportunities for exercise oncology professionals have become more available. The resulting exercise oncology workforce is comprised of a wide range of professionals with disparate skill sets that are difficult to quantify. This heterogeneity makes it hard to enact the practice and policy changes needed to support translation into clinical care, such as third‐party payer reimbursement for qualified exercise professionals.

As efforts continue to push for the establishment of routine integration of exercise into cancer care, there is a need to identify the core competencies required of the workforce capable of delivering this service. Therefore, the aim of this study was to obtain consensus on the knowledge, skills, and competencies (i.e., core competencies) for an exercise professional to be qualified to work with adults undergoing active cancer treatment. To achieve this, we followed a modified Delphi process that included an international panel of experts actively involved in exercise oncology services. Clarification of these competencies will underpin the development of future education, certification, and employment requirements for exercise professionals, representing a critical step to achieve routine integration into standard oncology care.

## 2 MATERIALS AND METHODS

This manuscript was guided by Delphi reporting guidelines outlined by Spranger et al. to ensure all required information was captured.^11^

We conducted a modified electronic Delphi study using a mixed methods approach that included three iterative rounds of electronic surveys. The Delphi process facilitates group consensus on a specific issue and has been used extensively in the development of health competencies across a variety of fields.^12^, ^13^, ^14^, ^15^ Online administration enables group discussion from a geographically diverse set of participants while maintaining anonymity and removing potential for domination of the group's opinion by individuals with a strong voice.^16^ The work was led by the MTC task force. Ethical approval was provided by Edith Cowan University's Human Research Ethics Committee (ID: 2021–02658 KENNEDY). All individuals provided informed consent prior to participation.

### 2.1 Participant recruitment

Experts from three stakeholder groups (i.e., exercise oncology professionals, clinical referrers, and people with lived experience of participating in exercise during cancer treatment) were identified using a purposive sampling approach.^17^ Recruitment was limited to those providing or receiving care in the US or Australia because these two countries have active calls for exercise integration; recruitment was driven through the MTC task force members' networks. An initial list of experts for each stakeholder group was generated according to the criteria below. Participant anonymity was retained throughout the process.

#### 2.1.1 Exercise oncology professionals

Exercise professionals known to be actively working in a clinical oncology setting to provide exercise programming to people on active cancer treatment were invited to participate. Invitees included people trained to prescribe exercise for people with chronic conditions (i.e., exercise physiologists, cancer exercise trainers) and licensed practitioners trained to improve a person's mobility and function, and/or restore optimal health to return to important activities of life (i.e., physical therapists/physiotherapists, occupational therapists). People whose experience was limited to research settings were excluded. Exercise oncology professionals were asked to complete all three rounds of the study as they are the only group with expertise to critically evaluate day‐to‐day competency requirements. An initial email invitation was sent, followed by a reminder approximately 3 weeks later. Invitations for the second and third rounds were sent approximately eight and 12 months after completion of the Round 1 survey. A nominal incentive was offered in the third‐round invitation for completion of the final survey. Participants who did not complete a given round were not invited into subsequent rounds.

#### 2.1.2 Clinical referrers

Clinicians known to actively provide exercise referrals to people receiving cancer treatment were invited to inform the competency development. The requirement to actively refer ensured participants could share a personal perspective about exercise oncology referrals. Clinical referrers were asked to participate in Part I of the Round 1 survey only, as they do not have the expertise to comprehensively assess the scope of work required by an exercise oncology professional. An initial email invitation was sent to identified experts. One reminder email was sent to nonrespondents 3 weeks later with no further contact.

#### 2.1.3 People with lived experience

People who participated in an exercise program while receiving cancer treatment were invited to inform competency development. They were asked to complete Part I of the Round 1 survey only as they do not have the expertise to comprehensively assess the scope of work required by an exercise oncology professional. In addition to referrals from the MTC task force, the exercise oncology professionals invited into the study were asked to invite potential participants for this group from their client list using an invitation email created by the study team.

### 2.2 Survey design

The survey was designed to confirm consensus across three rounds. There is no set standard for how to determine consensus in literature.^18^ A priori, consensus was set at ≥90% (summing both “absolutely essential” and “very important”) of respondents for each item. This high level of agreement was chosen because of the large volume of items and desire to focus only on the most crucial competencies while accounting for some variation in opinion. Each item was presented a maximum of two times. Proposed items that did not reach consensus the first time were represented for forced consensus (yes/no). Items that did not meet criteria to move forward on this subsequent round were removed.

#### 2.2.1 Round 1 survey: establishing agreement with existing competencies and brainstorming new ideas

The Round 1 survey contained two parts.

### 2.3 Part I

Participants' demographic and professional background information was confirmed, and then their personal reflections on the knowledge, skills, and abilities required for exercise oncology professionals were elicited through open‐ended questions to brainstorm ideas without bias.^19^ The Round 1 survey can be viewed in Supplemental File S1.

### 2.4 Part II

Ninety‐one competencies described across two internationally recognized exercise oncology certification programs commonly used in the field (i.e., ACSM‐CET and CanRehab) were presented. The list resulted from comparing the original ACSM‐CET (*n* = 79) and CanRehab (*n* = 31) competencies and removing duplicates. The resulting 91 competencies were presented across nine categories defined by ACSM (Table 4). Participants rated the level of importance of each competency using a 5‐point Likert scale (“absolutely essential”, “very important”, “of average importance”, “of little importance”, “not important at all”). Participants were also provided the option of “I'm not sure” for each statement. After rating each competency in a category, experts were asked whether the whole category should be required (yes/no) and to add any competencies that were not captured by the existing list.

#### 2.4.1 Round 2 survey: clarifying responses and establishing agreement with newly developed competencies

A description of overall key themes from Round 1 was summarized, and all competencies that achieved predefined consensus (≥90%) in Round 1 were presented at the beginning of each survey category. Competencies that did not achieve ≥90% agreement were represented with results of their consensus outcomes from Round 1; experts were asked to rate their agreement for inclusion (yes/no). For whole categories that did not reach predefined consensus, experts were asked to rate their agreement with whether the category and each of the competencies within it should remain included (yes/no). Finally, new competencies and categories generated in Round 1 were presented for experts to rate their level of importance according to a 4‐point Likert scale. The “not important at all” option was removed in this round based on the findings from Round 1. The Round 2 survey can be viewed in Supplemental File S2.

#### 2.4.2 Round 3 survey: final consensus and frequency/mastery

All competencies that achieved predefined consensus (≥90%) in Rounds 1 and 2 were presented with a summary of previous results, and experts were asked to rate how often each competency was used in practice (i.e., frequency) and what level of skill each required (i.e., mastery) according to a 4‐point Likert scale. Frequency options ranged from “rarely (less than monthly)” to “very frequently (daily)”; mastery options ranged from “advanced beginner skill” to “expert skill.” The option to select “I'm not sure” was provided for each. Competencies introduced in Round 2 that reached near consensus (80%–89%) were represented, and experts were asked to rate their agreement for inclusion (yes/no), as well as rate frequency and level of mastery for each. Competencies that did not reach consensus of at least 80% when introduced in Round 2 were removed to reduce participant burden based on the experience of Round 1 and 2 results. The Round 3 survey can be viewed in Supplemental File S3.

**FIGURE 1:**
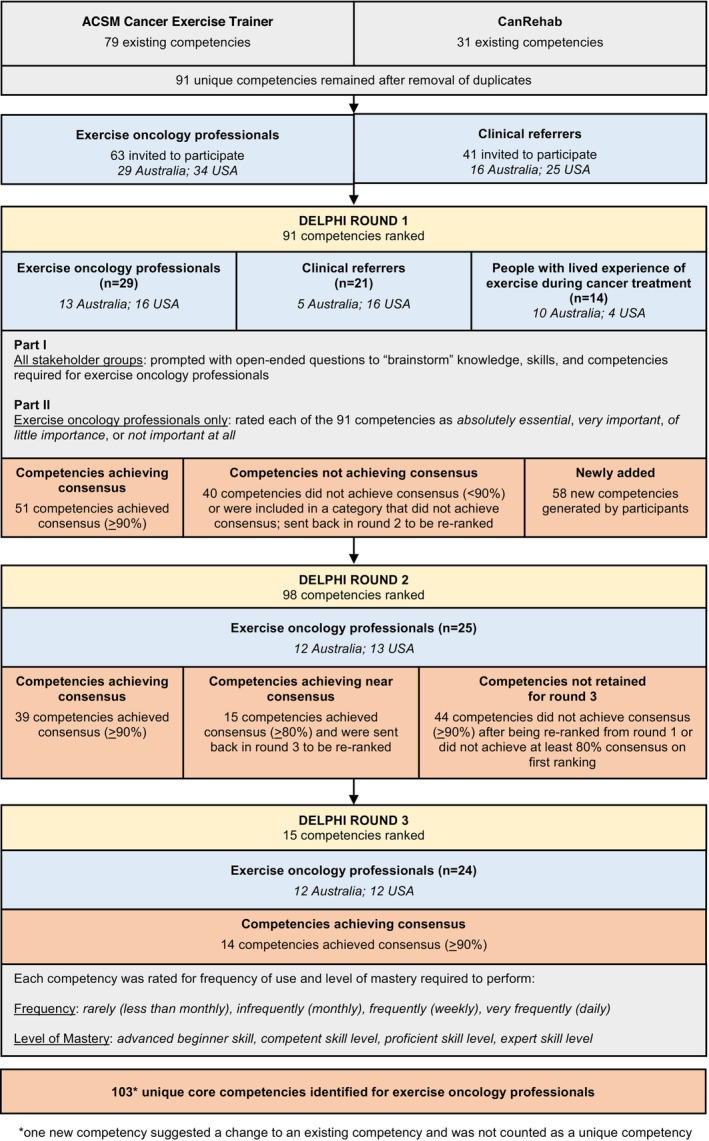
Flow diagram of the process of identifying core competencies for exercise oncology professionals. *One new competency suggested a change to an existing competency and was not counted as a unique competency.

The process to reach the core competencies is outlined in Figure 1.

## 3 DATA ANALYSIS

Frequency of Likert scale responses was calculated (SPSS for Mac, version 29). Two reviewers (MK and KW) independently assessed open‐ended responses provided in Round 1 to determine alignment with existing competencies (yes/no). Open‐ended responses determined to align were coded as duplicates and removed from further analysis. Responses presenting new content were inductively coded, grouped according to common themes, then aligned with an appropriate ACSM category for evaluation in subsequent rounds. New categories were created for responses that did not fit within an existing one. A third reviewer (CM) was brought in to offer an alternative viewpoint and review all responses as a form of member checking. Every decision underwent thorough discussion. In instances where the initial two reviewers disagreed, the third reviewer offered fresh insight to facilitate the resolution of all coding decisions.

## 4 RESULTS

### 4.1 Round 1, Part I

#### 4.1.1 Demographics

We invited 63 exercise oncology professionals and 41 clinical referrers to participate, from which 29 (46%) and 21 (51%) consented, respectively. Additionally, 14 people with lived experience participated. There was near equal representation from the US (56%; *n* = 36) and Australia (44%; *n* = 28) across all stakeholder groups (Tables 1, 2, 3).

**TABLE 1:** Characteristics of exercise oncology professionals.

	No. (%)		
	Round 1 (*n* = 29)	Round 2 (*n* = 25)	Round 3 (*n* = 24)
Age			
<30	8 (27.6)	6 (24.0)	5 (20.8)
31–40	13 (44.8)	13 (52.0)	13 (54.2)
41–50	6 (20.7)	5 (20.0)	5 (20.8)
51–60	1 (3.4)	0 (0.0)	0 (0.0)
61–70	1 (3.4)	1 (4.0)	1 (4.2)
Sex			
Female	19 (65.5)	17 (68.0)	16 (66.7)
Male	10 (34.5)	8 (32.0)	8 (33.3)
Racial and ethnic background			
White (not of Hispanic origin)	28 (96.6)	24 (96.0)	23 (95.8)
Black (not of Hispanic origin)	1 (3.4)	1 (4.0)	1 (4.2)
Country			
United States	16 (55.2)	13 (52.0)	12 (50.0)
Australia	13 (44.8)	12 (48.0)	12 (50.0)
Highest level of education			
Bachelor's degree	13 (44.8)	12 (48.0)	11 (45.8)
Master's degree	9 (31.0)	7 (28.0)	7 (29.2)
PhD/ScD	5 (17.2)	5 (20.0)	5 (20.8)
DPT	2 (6.9)	1 (16.7)	1 (4.2)
Type of exercise professional			
Exercise physiologist	20 (69.0)	18 (72.0)	17 (70.8)
Physio/physical therapist	7 (24.1)	5 (20.0)	5 (20.8)
Other^a^	2 (6.9)	2 (8.0)	2 (8.3)
Completed specific oncology training			
Yes	27 (93.1)	23 (92.0)	22 (91.7)
No	2 (6.9)	2 (8.0)	2 (8.3)
Years of experience in oncology			
0–5	8 (27.6)	6 (24.0)	5 (20.8)
6–10	12 (41.4)	11 (44.0)	11 (45.8)
11–15	3 (10.3)	3 (12.0)	3 (12.5)
16–20	4 (13.8)	4 (16.0)	4 (16.7)
21+	2 (6.9)	1 (4.0)	1 (4.2)
% of patients with cancer dx			
<25%	3 (10.3)	2 (8.0)	2 (8.3)
26–50	2 (6.9)	2 (8.0)	2 (8.3)
51%–75%	7 (24.1)	7 (28.0)	7 (29.2)
76%–99%	5 (17.2)	3 (12.0)	3 (12.5)
100%	12 (41.4)	11 (44.0)	10 (41.7)
New patients per month with cancer dx			
0–5	5 (17.2)	3 (12.0)	3 (12.5)
6–10	6 (20.7)	5 (20.0)	5 (20.8)
11–20	8 (27.6)	7 (28.0)	7 (29.2)
21–25	4 (13.8)	4 (16.0)	4 (16.7)
26–30	1 (3.4)	1 (4.0)	1 (4.2)
30+	5 (17.2)	5 (20.0)	4 (16.7)

Abbreviations: DPT, Doctorate of Physical Therapy; Dx, diagnosis; PhD, Doctorate of Philosophy.

^a^ Other includes cancer exercise trainer and occupational therapist/researcher.

**TABLE 2:** Characteristics of clinical referrers.

	(*n* = 21)
Age^a^	
31–40	12 (57.1)
41–50	2 (9.5)
51–60	6 (28.6)
Sex^a^	
Male	10 (47.6)
Female	10 (47.6)
Racial and ethnic background	
White (not of Hispanic origin)	15 (71.4)
Asian or Pacific Islander	2 (9.5)
Hispanic or Latino	2 (9.5)
Other	2 (9.5)
Country	
United States	16 (76.2)
Australia	5 (23.8)
Highest level of education	
Bachelor's degree	2 (9.5)
Professional degree (JD)	1 (7.1)
Doctoral degree	18 (85.7)
Type of clinician	
Medical oncologist	7 (33.3)
Physiatrist	7 (33.3)
Radiation oncologist	2 (9.5)
Surgical oncologist	2 (9.5)
Registered nurse	2 (9.5)
Occupational therapist	1 (4.8)
Years of experience in oncology	
0–5	4 (19.0)
6–10	7 (33.3)
11–15	4 (19.0)
16–20	2 (9.5)
21+	4 (19.0)

^a^ *n* = 1 = missing.

**TABLE 3:** Characteristics of people with lived experience.

	(*n* = 14)
Age	
31–40	1 (7.1)
41–50	3 (21.4)
51–60	2 (14.3)
61–70	6 (42.9)
70+	2 (14.3)
Sex	
Male	7 (50.0)
Female	7 (50.0)
Racial/ethnic background	
White (not of Hispanic origin)	11 (78.6)
Indian	3 (21.4)
Country	
Australia	7 (50.0)
United States	7 (50.0)
Highest level of education	
High school or equivalent	4 (28.6)
Associate degree	2 (14.3)
Bachelor's degree	2 (14.3)
Professional/Doctoral degree	2 (14.2)
Other	4 (28.6)
Cancer diagnosis	
Prostate	7 (50.0)
Breast	6 (42.9)
Other^a^	3 (21.3)
Have you been told your disease has spread	
Yes	7 (50.0)
No	7 (50.0)
What types of treatment have you undertaken	
Chemotherapy	10 (71.4)
Radiation	9 (64.3)
Surgery	6 (42.9)
Hormone therapy	6 (42.9)
Targeted therapy	1 (7.1)
What stage(s) of treatment did you participate in exercise?	
During chemotherapy	8 (57.1)
During radiotherapy	7 (50.0)
During hormone therapy	6 (42.9)
Before surgery	2 (14.3)
During targeted therapy	1 (7.1)

^a^ Other includes colon and rectal, uterine, and lung.

#### 4.1.2 Personal reflections

The open‐ended personal reflection questions generated 446 participant responses, resulting in 58 new items for consideration (Table 4). Most of the new competencies (*n* = 47; 81%) aligned with an existing ACSM category. A new category was created (personal skills & attributes) for the remaining competencies. Twenty responses described components of an ideal program structure. They were removed from further analysis as they were not specific to a professional's abilities and considered out of the scope of the project.

**TABLE 4:** Complete list of included competencies.

Specific knowledge, skill, and ability (KSA) for evaluation	Source	Frequency Mean (SD)	Mastery Mean (SD)
Category 1: Exercise physiology and related exercise science (consensus = 100%)			
1. Knowledge of physiologic outcomes that may be improved by exercise training among cancer survivors.	ACSM	3.88 (0.34)	3.04 (0.86)
2. Knowledge of symptoms and psychological attributes that may be improved by exercise training among cancer survivors.	ACSM	3.67 (0.56)	3.04 (0.81)
3. Knowledge of lymph, immunologic, cardiac, neurologic, endocrine, and hematologic systems as they pertain to cancer specific exercise issues. Incorporate musculoskeletal into this item^a^.	ACSM Participant addition	3.75 (0.44)	3.08 (0.88)
4. Knowledge of cancer diagnosis and treatment effects on physiological response to acute and chronic exercise, particularly with regard to physical deconditioning, body composition changes, and range of motion.	ACSM + CanRehab	3.83 (0.38)	3.33 (0.82)
5. Understand the emerging evidence regarding the potential effects of exercise on the physiology of cancer treatment (e.g., accelerated aging).	Participant addition	3.21 (0.66)	2.88 (0.95)
6. Understand the impact of exercise on oncology related comorbidities such as cardiotoxicity, diabetes, etc.	Participant addition	3.58 (0.50)	3.13 (0.80)
7. Understand how exercise can impact cognition and mental health.	Participant addition	3.25 (0.68)	2.75 (0.85)
8. Understand how exercise can assist cancer patients across the disease spectrum (diagnosis, treatment, recovery, palliative care).	Participant addition	3.67 (0.57)	3.25 (0.85)
Category 2: Health appraisal, fitness, and clinical exercise testing (93%)			
1. Ability to obtain a basic history regarding cancer diagnosis (e.g., type, stage) and treatment (e.g., surgeries, systemic, and targeted therapies).	ACSM	3.75 (0.53)	3.04 (1.1)
2. Knowledge of and the ability to recognize the adverse acute, chronic, and late effects of cancer treatments.	ACSM	3.63 (0.50)	3.33 (0.76)
3. Ability to obtain medical history for other health conditions (e.g., neurological, cardiovascular, musculoskeletal, pulmonary) that may co‐occur and interact with adverse effects of cancer treatments.	ACSM	3.63 (0.58)	3.04 (0.81)
4. Knowledge of and ability to discuss physiologic systems affected by cancer and treatment and how this would affect the major components of fitness, including balance, agility, speed, flexibility, endurance, and strength.	ACSM	3.63 (0.50)	3.21 (0.66)
5. Knowledge of how cancer and its treatments may alter balance, agility, speed, flexibility, endurance, and strength in cancer survivors and ability to select/modify and interpret tests of these fitness elements.	ACSM	3.78 (0.52)	3.38 (0.77)
6. Knowledge of how cancer and its treatments may affect body composition in cancer survivors and ability to select/modify and interpret tests of body composition in cancer survivors.	ACSM	3.29 (0.86)	3.00 (0.78)
7. Knowledge of categories of patients that require medical clearance prior to testing or exercise prescription.	ACSM	3.42 (0.78)	3.13 (0.85)
8. Knowledge of cancer‐specific relative and absolute contraindications to exercise testing.	ACSM	3.21 (0.83)	3.25 (0.79)
9. How to assess, interpret and record a client's baseline parameters within the categories of cardio‐respiratory endurance, muscular strength and endurance, flexibility, range of motion, balance, and body composition based on their physical and psychological parameters related to their cancer but also considering other associated medical conditions such as diabetes, anxiety, depression, hypertension, arthritis, osteoporosis, and cardiac disease which may be associated with cancer treatments.	CanRehab	3.63 (0.46)	3.21 (0.72)
10. Individual risk stratification using recognized guidelines.	CanRehab	3.21 (0.66)	3.04 (0.75)
11. Ability to perform a subjective interview to understand patient's goals and patient burden of symptoms from cancer or cancer treatment.	Participant addition	3.71 (0.55)	3.00 (0.78)
12. Ability to develop and use appropriate assessment protocols.	Participant addition	3.46 (0.72)	3.13 (0.85)
13. Ability to effectively review medical chart notes to understand cancer diagnosis (e.g., stage/grade of cancer) and treatments.	Participant addition	3.46 (0.72)	2.96 (0.75)
Category 3: Exercise prescription and programming (97%)			
1. Knowledge of current guidelines for exercise in cancer survivors.	ACSM + CanRehab	3.46 (0.78)	3.04 (0.96)
2. Ability to describe benefits and risks of exercise training in the cancer survivor.	ACSM + CanRehab	3.79 (0.42)	3.13 (0.99)
3. Ability to recognize relative and absolute contraindications for starting or resuming an exercise program, and knowledge of when it is necessary to refer participant back to an appropriate care provider or when they are eligible for referral to community‐based exercise programs.	ACSM + CanRehab	3.29 (0.91)	3.42 (0.65)
4. Knowledge of potential for overtraining with the cancer survivor.	ACSM	2.96 (0.81)	2.96 (0.69)
5. How to design an individualized exercise program based on the initial assessment.	CanRehab	3.71 (0.46)	3.42 (0.65)
6. How to determine which baseline parameters can be monitored during the forthcoming exercise program in order to assess ongoing effectiveness and if necessary modify the program and offer alternative exercises.	CanRehab	3.33 (0.70)	3.22 (0.60)
7. Knowledge, skill, and ability to undertake appropriate ongoing screening in order to detect a change in condition and modify exercise prescription/program based on a current medical condition.	ACSM + CanRehab	Frequency	Mastery
8. Knowledge, skill, and ability to undertake appropriate ongoing screening in order to detect a change in condition and modify exercise prescription/program based on time since diagnosis on or off adjuvant treatment.	ACSM + CanRehab	3.25 (0.68)	3.29 (0.69)
9. Knowledge, skill, and ability to undertake appropriate ongoing screening in order to detect a change in condition and modify exercise prescription/program based on type of current therapies (e.g., no swimming during radiation).	ACSM + CanRehab	3.33 (0.57)	3.29 (0.62)
10. Knowledge, skill, and ability to undertake appropriate ongoing screening in order to detect a change in condition and modify exercise prescription/program based on type and recency of surgical procedures (e.g., curative or reconstructive).	ACSM + CanRehab	3.17 (0.63)	3.33 (0.57)
11. Knowledge, skill, and ability to undertake appropriate ongoing screening in order to detect a change in condition and modify exercise prescription/program based on range of motion.	ACSM + CanRehab	3.21 (0.78)	3.08 (1.02)
12. Knowledge, skill, and ability to undertake appropriate ongoing screening in order to detect a change in condition and modify exercise prescription/program based on the presence of implants.	ACSM + CanRehab	2.67 (0.92)	2.92 (0.83)
13. Knowledge, skill, and ability to undertake appropriate ongoing screening in order to detect a change in condition and modify exercise prescription/program based on amputations/fusions.	ACSM + CanRehab	Frequency	Mastery
14. Knowledge, skill, and ability to undertake appropriate ongoing screening in order to detect a change in condition and modify exercise prescription/program based on effects of treatment on all elements of fitness (agility, speed, coordination, flexibility, strength, and endurance).	ACSM + CanRehab	2.13 (0.90)	3.00 (0.83)
15. Knowledge, skill, and ability to undertake appropriate ongoing screening in order to detect a change in condition and modify exercise prescription/program based on hematologic considerations (e.g., anemia, neutropenia).	ACSM + CanRehab	3.04 (0.81)	3.25 (0.61)
16. Knowledge, skill, and ability to undertake appropriate ongoing screening in order to detect a change in condition and modify exercise prescription/program based on presence of a central line (PICC or Port).	ACSM + CanRehab	2.83 (0.57)	3.13 (0.61)
17. Knowledge, skill, and ability to undertake appropriate ongoing screening in order to detect a change in condition and modify exercise prescription/program based on current adverse effects of treatment, both acute and chronic.	ACSM + CanRehab	3.63 (0.50)	3.38 (0.65)
18. Knowledge, skill, and ability to undertake appropriate ongoing screening in order to detect a change in condition and modify exercise prescription/program based on individuals that may be at increased risk for adverse late effects that could increase risks associated with exercise (e.g., heart failure).	ACSM + CanRehab	3.00 (0.72)	3.21 (0.66)
19. Ability to safely and appropriately progress exercise to ensure an appropriately intense exercise dose to stimulate desired adaptations while minimizing risk is important to ensure not only safety but also efficacy of exercise.	Participant addition	3.63 (0.58)	3.33 (0.87)
20. Ability to adapt the program on demand in response to highs and lows of energy, emotion or function.	Participant addition	3.54 (0.51)	3.13 (0.80)
21. When to start resistance based exercises.	Participant addition	3.58 (0.50)	2.96 (0.91)
22. Knowledge of how to add progressive overload in an exercise prescription while also finding the balance between what is enough, but what is not too much.	Participant addition	3.71 (0.55)	3.29 (0.86)
23. Provide education and strategies for pacing activity throughout the day outside of physical exercise activities, including avoiding sedentary behaviors.	Participant addition	3.33 (0.48)	2.79 (0.88)
24. Ability to effectively use the Borg Scale or other perceived exertion charts.	Participant addition	3.63 (0.58)	2.83 (1.11)
25. Ability to identify and use appropriate tools to monitor progress.	Participant addition	3.33 (0.64)	2.88 (1.12)
Category 4: Nutrition and weight management (76%)			
*Category did not reach consensus* ^b^			
Category 5: Human behavior and counseling (93%)			
1. Knowledge to identify a teachable moment for cancer survivors and ability to use that time to provide appropriate information and education about resuming or adopting an exercise program.	ACSM	3.29 (0.62)	3.13 (0.78)
2. General knowledge of psychosocial problems common to cancer survivors, such as depression, anxiety, fear of recurrence, sleep disturbances, body image, sexual dysfunction, and work and marital difficulties.	ACSM	3.38 (0.58)	2.96 (0.86)
3. Knowledge of behavioral strategies that can enhance motivation and adherence (e.g., goal setting, exercise logs, planning).	ACSM + CanRehab	3.38 (0.58)	2.96 (0.91)
4. Knowledge of the impact of cancer diagnosis and treatment on quality of life (QOL), and the potential for exercise to enhance a range of QOL outcomes for survivors (e.g., sleep, fatigue, and other factors).	ACSM	3.58 (0.50)	3.09 (0.90)
5. Knowledge of how cancer and cancer treatment relate to ability and readiness to start an exercise program.	ACSM	3.46 (0.66)	3.17 (0.82)
6. Demonstrate communication skills and compassion for patients/clients who have suffered the physical and psychological trauma of cancer and its management.	CanRehab	3.67 (0.48)	3.04 (0.81)
7. Understand the patient's goals for exercise and know how to use them to set realistic expectations for exercise.	Participant addition	3.46 (0.59)	2.92 (0.88)
8. Demonstrate an understanding of the patient's personal circumstances, needs, and concerns relating to their cancer treatment.	Participant addition	3.21 (0.72)	2.79 (0.88)
9. Understand common barriers to (and facilitators of) exercise and be able to work with patients to overcome as many as possible.	Participant addition	3.50 (0.51)	3.0 (0.83)
10. Know when and how to refer to and collaborate with Registered Dieticians.^c^	Participant addition	2.83 (0.76)	2.58 (0.88)
Category 6: Safety, injury prevention, and emergency procedures (consensus = 97%)			
1. Knowledge of and ability to recognize and respond to cancer‐specific safety issues, such as: susceptibility to infection, musculoskeletal and orthopedic changes, unilateral edema, fatigue, lymphedema, neurological changes, osteoporosis, and cognitive decline associated with treatment.	ACSM + CanRehab	3.29 (0.75)	3.33 (0.57)
2. Knowledge of and ability to respond to cancer specific emergencies, including: sudden loss of limb function, fever in immune‐incompetent patient, and mental status changes.	ACSM + CanRehab	2.29 (1.09)	3.21 (0.88)
3. Knowledge of and ability to respond to the signs and symptoms of new onset and major life threatening complications of cancer, such as superior vena cava syndrome (SVCS), sepsis or infection, and spinal cord compression.	ACSM + CanRehab	1.71 (0.91)	3.38 (0.92)
4. Knowledge of and ability to write‐up incident documentation related to cancer specific adverse events.	ACSM	1.67 (0.87)	3.00 (0.72)
Category 7: Program administration, quality assurance, and outcome assessment (consensus = 93%)			
1. How to establish a safe and stimulating activity environment sensitive to the physical and psychological, confidentially needs of patients/clients with cancer including the appropriateness of group or individual therapies.	CanRehab	3.25 (0.73)	2.96 (0.93)
2. Select appropriate objective outcome measures to address needs raised patient history, including Patient Related Outcome Measures (PROMS) and quality of life assessments.	Participant addition	3.09 (0.85)	2.96 (0.88)
3. Establish collaborative working professional relationships with the oncology treatment and cancer rehabilitation teams where possible.	Participant addition	2.92 (0.78)	3.25 (0.68)
4. Understand your role as part of a multidisciplinary care team.	Participant addition	3.25 (0.79)	3.08 (0.72)
Category 8: Clinical and medical considerations (96%)			
1. Knowledge of the major long‐term effects among childhood cancer survivors that may require careful screening and program adaptation for these individuals.	ACSM	2.00 (0.89)	3.17 (0.70)
2. Knowledge of the common side effects and symptoms of typical cancer treatments (surgeries, chemotherapy, radiation, hormone manipulations, other drugs).	ACSM + CanRehab	3.75 (0.44)	3.25 (0.85)
3. Knowledge that cancer treatment may accelerate functional decline associated with aging, particularly in the elderly, and that exercise programming may need to be adjusted accordingly.	ACSM	3.42 (0.65)	3.13 (0.74)
4. Knowledge of the combined effects of aging and cancer‐treatment on exercise capacity and selection of appropriate testing modalities and interpretation of results.	ACSM	3.26 (0.54)	3.29 (0.75)
5. Knowledge of the common sites of metastases and ability to design and implement appropriate exercise programs consistent with this knowledge.	ACSM	3.04 (0.75)	3.42 (0.72)
6. Knowledge of the signs and symptoms associated with new onset lymphedema, and the major cancer types associated with increased lymphedema risk (e.g., breast, head, and neck cancer).	ACSM	2.92 (0.78)	3.25 (0.79)
7. Knowledge of lymphedema risk reduction practices, and exercise guidelines.	ACSM	3.04 (0.75)	3.17 (0.87)
8. Knowledge of how cancer treatment may alter cardiovascular risk factors, and inappropriate far responses to exercise testing or training.	ACSM	3.08 (0.83)	3.00 (0.78)
9. Knowledge of lymphatic, neurological and immune system factors in cancer survivors that may require further evaluation by medical or allied health professionals before participation in physical activity.	ACSM	2.88 (0.90)	3.25 (0.74)
10. Knowledge of how common cancer treatments affects the ability of cancer survivors to perform exercise, and how to adjust programs accordingly.	ACSM	3.58 (0.50)	3.30 (0.64)
11. Knowledge of the effect of cancer treatment on balance and mobility and the ability to develop an appropriate exercise program that minimizes fall/injury risk.	ACSM	3.50 (0.66)	3.13 (0.95)
12. Knowledge and ability to recognize the limits in the scope of practice for exercise professionals in working with cancer survivors with complex medical issues.	ACSM	3.17 (0.57)	3.17 (0.70)
13. Be familiar with and able to interpret medical information in the context of exercise prescriptions.	Participant addition	3.46 (0.72)	3.22 (0.74)
14. Know common cancer pathophysiology, staging, grading, type of cancer (e.g., TNM score and how this impacts exercise prescription and precautions to consider or implement, etc).	Participant addition	3.29 (0.75)	3.04 (0.86)
15. Understand breast reconstruction.	Participant addition	2.88 (0.68)	3.00 (0.89)
16. General tissue healing timeframes, to then apply to exercise prescription postsurgery as core foundational knowledge.	Participant addition	2.96 (0.69)	3.04 (0.69)
17. Understand the symptoms specific to typical presentation of various cancer diagnoses.	Participant addition	3.39 (0.72)	3.08 (0.88)
18. Ability to identify potential signs of skeletal metastases progression that may warrant further investigation.	Participant addition	2.08 (0.97)	3.21 (0.78)
19. Knowledge of the expected effects of treatment and their impact on patients' ability to exercise (i.e., when patients will feel well or unwell during a treatment cycle).	Participant addition	3.38 (0.58)	3.17 (0.76)
20. Knowledge of common effects of cancer treatment on energy balance and body composition for individuals with nonmetastatic disease.^c^	ACSM	3.17 (0.57)	3.04 (0.81)
21. Knowledge of effects of cancer cachexia on energy balance, intake, and activity level among individuals with metastatic disease.^c^	ACSM	2.96 (0.75)	3.38 (0.58)
22. Ability to discern when a participant's nutritional status would be best managed by referral to a registered dietitian.^c^	ACSM	3.17 (0.76)	2.75 (0.94)
Category 9: Physiology, diagnosis, and treatment (93%)			
1. Knowledge of the most common warning signs of recurrence for common cancers, and when to recommend that clients seek additional medical evaluation.	ACSM	2.13 (0.87)	3.13 (0.92)
2. General knowledge of current cancer treatment strategies, including surgery, systemic therapies (e.g., chemotherapy) and targeted therapies (e.g, anti‐angiogenesis inhibitors).	ACSM + CanRehab	3.43 (0.66)	3.13 (0.82)
3. Knowledge of how lifestyle factors, including nutrition, physical activity, and heredity, influence hypothesized mechanisms of cancer etiology, reduce the risk of relapse after initial treatments, and improve long‐term survival.	ACSM + CanRehab	3.26 (0.86)	3.00 (0.95)
4. Understand whether the goal of treatment is curative or palliative and recognize how to support a patient through each scenario.	Participant addition	3.13 (0.92)	3.13 (1.01)
5. Be aware of and keep up‐to‐date with current research and best practice methods in the field.	Participant addition	2.57 (0.66)	3.13 (0.87)
6. Recognize potential side effects of a patient's medications and potential contraindications for exercise.	Participant addition	3.17 (0.78)	3.09 (0.85)
Category 10: Personal skills and attributes			
1. Ability to be flexible with programming based on a patient's needs.	Participant addition	3.70 (0.47)	3.30 (0.77)
2. Verbal and written communication skills necessary to clearly describe programming goals, expectations, and patient progress to both patients and clinicians.	Participant addition	3.74 (0.54)	3.17 (0.89)
3. Ability to empathize with patients.	Participant addition	3.96 (0.21)	3.00 (1.04)
4. Listening skills.	Participant addition	4.00 (0.00)	2.91 (1.08)
5. Ability to observe patient needs and respond accordingly.	Participant addition	4.00 (0.00)	3.09 (1.11)
6. Ability to manage patient programming in an organized and efficient manner.	Participant addition	3.87 (0.46)	2.91 (1.04)
7. Demonstrate patience in approach to a patient's needs.	Participant addition	3.83 (0.39)	2.87 (1.01)
8. Ability to establish rapport with patients in a therapeutic relationship.	Participant addition	3.91 (0.29)	2.96 (1.15)
9. A positive approach aiming to make exercise as enjoyable as possible for the patient.	Participant addition	3.70 (0.56)	2.74 (1.01)
10. Problem solving/critical thinking skills.	Participant addition	3.87 (0.34)	3.13 (0.92)
11. Be willing to accept feedback for programming and professional improvement.	Participant addition	3.30 (0.70)	2.83 (1.03)

Abbreviation: ACSM, American College of Sports Medicine.

^a^ Musculoskeletal incorporated to this item.

^b^ Four items that reached consensus in this category distributed to other categories.

^c^ Originally part of eliminated category = Category 4: Nutrition and weight management.

*Note*: Frequency: (1) Rarely (less than monthly); (2) Infrequently (monthly); (3) Frequently (weekly); (4) Very frequently (daily). Mastery: (1) Advanced beginner skill; (2) Competent skill level; (3) Proficient skill level; (4) Expert skill level.

### 4.2 Round 1, Part II

Twenty‐nine exercise oncology professionals completed Part II of the Round 1 survey. Six of the nine (67%) categories and 51 of the 91 (56%) competencies achieved consensus. Round 1 results are reported in Supplemental File S4.

### 4.3 Round 2

Twenty‐five exercise oncology professionals completed Round 2 (86% participant retention). Two of the three (67%) categories and 7 of the 40 (18%) competencies that were reranked achieved consensus. The one new category and 29 of the 58 (50%) new competencies achieved consensus, the remaining 30 were evenly split (*n* = 15; 25% each) between being removed and being reranked in Round 3. Round 2 results are reported in Supplemental File S4.

### 4.4 Round 3

Twenty‐four exercise oncology professionals completed Round 3 (83% participant retention from Round 1; 96% from Round 2). Fourteen of the 15 (93%) competencies that were reranked achieved consensus resulting in a total of 103 items for inclusion in the final set of core competencies. Of these, the majority were rated as being performed frequently or very frequently (75%) and requiring a proficient or expert level of mastery (83%). A summary of Round 3 results is provided in Table 4; complete results are reported in Supplemental File S4.

## 5 DISCUSSION

Integration of exercise into standard oncology care requires a highly skilled workforce of exercise professionals; however, competencies requirements for this workforce have not kept pace with advancements in the field. The resultant qualification requirements for professional practice remain unclear. This study used a modified Delphi process to gain consensus for the core competencies required for exercise professionals to be able to work with adults undergoing active cancer treatment. A total of 149 competencies were ranked across three survey rounds over a 12‐month period. Consensus for 103 core competencies was achieved. These competencies will underpin the development of future education, certification, and professional practice requirements for exercise professionals to move toward widespread integration into standard oncology care.

The core competencies identified in this study reflect a maturation of the field of exercise oncology in the 15+ years since existing certifications were created by ACSM and CanRehab. Nearly half (*n* = 43) of the final 103 competencies were new suggestions from the panel of experts. Further, 67% (*n* = 30) of the 45 competencies not achieving consensus came from the original ACSM and/or CanRehab list. This shift reflects our evolving understanding and acceptance of the role exercise during cancer treatment. The literature available for the first iteration of exercise oncology guidelines largely examined the safety, feasibility, and efficacy of exercise.^5^, ^20^ In 2018, when the literature was rereviewed,^4^, ^5^ the exponential growth in high‐quality research across the field allowed for the provision of specific exercise prescription guidance. Practical application of exercise oncology research in clinical practice also increased during this time as oncology clinicians and people who have received a cancer diagnosis have begun to recognize the benefits exercise provides.^6^, ^21^

The study supports the need for workforce training to be exercise specific. Consensus was low for competencies that described context‐dependent skills (e.g., working with a medical record), skills not directly related to exercise (e.g., sun exposure, nutrition), very specialized skills (e.g., lead balance exercises), and skills related to general program administration. This shift toward exercise‐focused competencies reflects the movement toward multi‐disciplinary care teams allowing for each professional to remain highly specialized.^22^

While most (81%) of the 58 newly generated competencies aligned with an existing ACSM category, one new category was developed (personal skills & attributes). This new category primarily describes skills required to care for people based on their personal needs and preferences, not simply the requirements of their condition.^23^ This person‐centered approach to care is the recommended model of cancer care across disciplines.^24^ Consensus to recognize skills such as “ability to empathize with patients” and “demonstrate patience in approach to a patient's needs” as core competencies in exercise oncology demonstrates professionals' alignment with delivery of best practice cancer care.

Personal reflections from clinical referrers and people with lived experience of exercising during cancer treatment offered important implementation considerations for the future of the exercise oncology certification processes. Clinical referrers' input focused on the need for trust in the exercise professionals abilities (e.g., good reputation, qualified, experienced). This is unsurprising based on the “trust gap” that limits referrals between clinicians and exercise professionals^25^; however, it emphasizes the need for standardization of exercise oncology credentialing. Feedback from those with lived experience highlights a potential need for a practicum component to be included in the certification process. Their comments strongly aligned with the human behavior and counseling category, which includes skills that require an exercise professional to extend beyond book knowledge. Incorporation of a requirement for the demonstration of practical skills may also increase the validity of the certification among clinicians. The field of health coaching offers an exemplar for this certification approach.^26^

This study used a recognized expert consensus building process, inclusive of international and multiple stakeholder perspectives, to identify competencies for the exercise oncology workforce. However, there are limitations in its design. First, as there is no standard definition of a modified Delphi approach,^18^ the decision to force agreement across three rounds was made to reduce participant burden because of the large number of items to be assessed. Given the high level of agreement, it is unlikely results would have been significantly different with additional rounds. Next, the study included experts from only two countries. While methods of program delivery vary across countries, the guidelines for exercise oncology professionals are internationally accepted.^4^, ^5^ Finally, these competencies reflect the highest level of care necessary, as the study specifically asked about working with adults actively receiving cancer treatment. Future research should investigate how to create a stepped certification program to reflect the skill sets required for delivering exercise programming to people at different stages of the cancer continuum.^27^, ^28^

In conclusion, widespread agreement about the required competencies for exercise oncology professionals will allow for the creation of new training models and propel parallel initiatives required to achieve integration into standard oncology care.^9^ For example, in the US, while physical therapists can receive third‐party reimbursement for their services, other exercise professionals cannot. There are currently not enough oncology‐trained physical therapists to meet patient demand. Further, the current payment methodology for physical therapist services aligns with an impairment model of rehabilitation, which may limit wellness‐focused exercise interventions. The workforce capable of billing for their services needs to be expanded to ensure a cost‐effective solution to meet patient demand. The establishment of standardized competencies will help to streamline the skillset that can be expected from exercise professionals with an oncology “certification.” This standardization will facilitate policy change efforts to enable all exercise oncology professionals to receive third‐party payer reimbursement. The credentialing process for Accredited Exercise Physiologists (AEP) in Australia provides an exemplar for a standardized credential that has achieved government support.^29^ While work needs to be done to optimize the integration of AEPs into the Australian Medicare system, the national standardization of the AEP credential facilitates a clear understanding of the minimum training and competency level that can be expected of all exercise professionals in the country. Further, while current training programs for both physical therapists and exercise physiologists include some of the 103 core competencies described in this study, neither covers all. To embed exercise into standard oncology care, the workforce—inclusive of all exercise professionals and physical therapists—needs to be upskilled to be able to provide the high‐quality care required of referring clinicians and expected by patients. This study represents a foundational step toward achieving that goal; however, future work needs to focus on the development of implementation strategies to promote the use of the identified competencies in all exercise oncology training and certification programs.

## 6 CONCLUSION

The core competencies identified through this study reflect significant advancements made in the field of exercise oncology. Results will underpin future education, certification, and employment requirements for exercise oncology professionals and allow for widespread development of the workforce, which represents a critical step toward achieving routine integration of exercise into standard oncology care.

## AUTHOR CONTRIBUTIONS

**Mary A. Kennedy:** Conceptualization (equal); data curation (lead); formal analysis (lead); methodology (equal); project administration (lead); resources (equal); writing – original draft (lead); writing – review and editing (equal). **Kelley Covington Wood:** Conceptualization (equal); formal analysis (supporting); methodology (equal); project administration (supporting); writing – original draft (supporting); writing – review and editing (equal). **Anna Campbell:** Conceptualization (equal); methodology (equal); writing – original draft (supporting); writing – review and editing (equal). **Melanie Potiaumpai:** Conceptualization (equal); methodology (supporting); writing – review and editing (equal). **Christopher M. Wilson:** Conceptualization (equal); methodology (supporting); writing – review and editing (equal). **Anna L. Schwartz:** Conceptualization (equal); methodology (supporting); writing – review and editing (equal). **Jessica Gorzelitz:** Conceptualization (equal); methodology (supporting); writing – review and editing (equal). **Maxime Caru:** Conceptualization (equal); methodology (supporting); writing – review and editing (equal). **Kathryn H. Schmitz:** Conceptualization (equal); formal analysis (supporting); funding acquisition (lead); investigation (equal); methodology (equal); project administration (supporting); resources (supporting).

## FUNDING INFORMATION

Dr. Schmitz is supported by American Cancer Society CRP‐22‐081‐CTPS.

## CONFLICT OF INTEREST STATEMENT

No authors declare any conflicts of interest.

## Supporting information


Data S1.

## Data Availability

The authors confirm that the data supporting the findings of this study are available within the article and its supplementary materials.
