# Supplementary material for: Identification of core competencies for exercise oncology professionals: A Delphi study of United States and Australian participants
Source: Cancer Med. 2024 Jul 24;13(14):e70004. doi: 10.1002/cam4.70004 (PMC11267632; doi:10.1002/cam4.70004)
Supplement: Supplementary file 1 — Data S1. [file CAM4-13-e70004-s001.zip › S1.Round1 Survey.docx]

# Exercise Oncology Workforce Delphi

Thank you for sharing your time and expertise with us. Your responses will help to define the specialized skillset and training requirements an exercise professional needs to deliver exercise programming to people with cancer and move us closer to the goal of making exercise standard practice in oncology.

Exercise oncology workforce development: A Delphi I agree to proceed

study I do not wish to participate


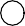

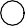


We are conducting a Delphi study to reach expert consensus on the knowledge, skills, and competencies required for exercise oncology professionals to work with people in an oncology setting.

Your participation in this research project will involve answering questions in an online survey. You will be sent an email link to a series of online surveys over the course of the project. You will be asked to provide your email address in the survey. It will only be used to contact you for the follow-up surveys that are part of this study. It will not be

shared with anyone and will not be used for any other purposes.

Your participation is voluntary. If you do not wish to take part, you do not have to. Your decision to take part in the study will not impact your future involvement with exercise oncology initiatives or opportunities to collaborate with any of the study’s investigator team.

There are no direct benefits of taking part. We hope the research leads to an improved understanding of workforce development needs for exercise oncology professionals, which will help exercise become a standard part of oncology care in the future.

There are no foreseeable risks to participation. In the unlikely event that any questions cause you concern you should let the researcher know. If you do

not wish to answer a question, you may request to skip it, or you may stop immediately.

Results will be published in research journals or presented at research conferences. Your name or any other identifying information will not be included in any of the publications or presentations.

By agreeing to participate you are consenting to take part in the research project and have your personal information used as described above.

**Demographic and Professional Information**

**The following section asks questions about you, your professional background, and your experience with exercise oncology.**

What is your first name?

What is your last name?

What is your email address?

What is your age? < or = 30

31 - 40


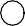

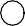

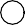

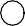

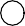

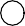


41 - 50

51 - 60

61 - 70

71+

What is your sex? Male

Female Other


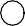

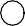

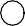

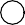


Prefer not to say

What is the highest level of education you have High school or equivalent

completed? Associate's degree


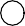

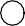

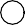

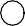

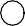

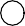

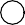


Bachelor's degree Master's degree Professional degree Doctoral degree Other

Please specify what type of doctoral degree (e.g. PhD, MD/MBBS MD, DPT) PhD/ScD

DPT


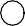

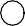

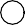

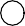


Other

Please specify "other"

Please specify which professional degree (e.g. MBA, JD)

Please specify "other"

What best describes your racial/ethnic background? American Indian or Alaskan Native

Aboriginal or Torres Strait Islander Asian or Pacific Islander


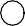

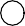

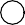

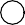

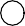

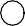

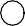


Black (not of Hispanic origin) White (not of Hispanic origin) Hispanic/Latino

Other

Please specify "Other"

What stakeholder group do you represent? Exercise oncology professional


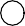

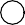


Clinician who refers patients to exercise oncology programs


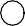
 Cancer survivor


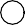

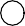

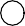


What type of exercise professional are you? Exercise physiologist/Exercise scientist Physiotherapist/Physical Therapist Other

Please specify "Other"

What type of clinician are you? General/Primary Care Practitioner Surgical oncologist

Medical oncologist Radiation oncologist Nurse practitioner Registered nurse Physiatrist


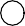

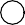

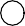

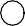

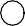

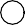

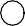

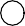


Other

Please specify "Other"

What type of cancer have you been diagnosed with?
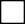
 Bladder

Select as many diagnoses as apply.
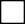
 Breast


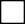
 Colon and Rectal
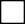
 Endometrial


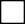
 Kidney


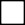
 Leukemia (all types)


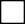
 Liver and Intrahepatic Bile Duct
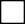
 Lung (including Bronchus)


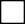
 Melanoma


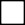
 Non-Hodgkin Lymphoma
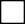
 Pancreas


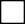
 Prostate
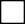
 Thyroid
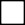
 Other

Please specify "Other"

Has you been told your disease has spread? Yes No

What types of treatment have you undertaken or are
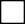
 Surgery

currently undertaking? Check all that apply.
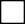
 Chemotherapy
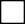
 Radiation


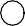

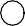


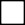
 Hormone therapy
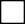
 Immunotherapy
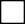
 Targeted therapy
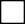
 Other

At what stage(s) did you participate in exercise
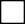
 Before surgery

during treatment? Check all that apply.
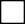
 During chemotherapy
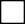
 During radiotherapy


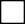
 During hormone therapy
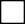
 During immunotherapy
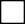
 During targeted therapy
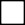
 Other

Where are you employed?

What organization were you referred to for exercise before and/or during treatment?

Who referred you to take part in this survey (first, last name)?

What is the total number of years you have worked as 0 - 5 years an oncology professional? 6 - 10 years

11 - 15 years


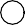

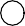

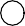

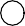

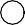

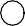


16 - 20 years

21+ years Not applicable

In the last year, what percentage of your patient < or = 25%

population had a cancer diagnosis? 26 - 50% 51 - 75%


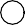

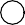

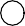

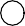

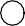


76 - 99%

100%

Of the patients with cancer you saw in the last year, what proportion were curative? For example if 80% were

curative, slide bar to 80. 0 50 100

*(Place a mark on the scale above)*

Of the patients you saw with cancer in the last year,
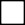
 Bladder what were the three most common types you treated?
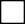
 Breast

Please select three choices.
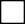
 Colon and Rectal
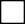
 Endometrial


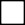
 Kidney


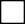
 Leukemia (all types)


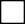
 Liver and Intrahepatic Bile Duct
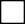
 Lung (including Bronchus)


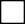
 Melanoma


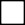
 Non-Hodgkin Lymphoma
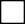
 Pancreas


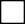
 Prostate
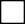
 Thyroid
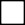
 Other

Please specify "Other"

In the last year, on average, how many new patients 0 - 5


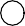


with a cancer diagnosis did you work with each month? 6 - 10

11 - 20

21 - 25

26 - 30

30+

In the last year, on average, how many new patients < or = 25% with a cancer diagnosis did you refer to exercise each 26 - 50% month? 51 - 75%

76 - 99%

100%

Do you have any specific considerations when referring I only refer people with specific diagnoses patients to exercise? Please select all that apply. I only refer people of a certain age range

I only refer people who have previously been active I refer most patients to exercise unless there is

a clear contraindication Other

Please specify what patient diagnoses you refer to

exercise (e.g. breast only, curative only, etc.)

Please specify what age range you refer to exercise

(e.g. under 80 years only.)

Please specify other with regard to what

considerations you make regarding the types of patients you refer to exercise.

What is the age range of the patients you routinely Less than 21 years of age work with? Check all that apply. 21 - 65 years of age

65+ years of age

Have you completed any additional ACSM Certified Cancer Exercise Trainer

training/professional development or hold ACE Cancer Exercise Specialist licenses/certification in exercise oncology? Please CanRehab Cancer and Exercise Rehabilitation Level check all of the following courses you have completed. 4 Qualification

CETI Cancer Exercise Specialist

If yours is not listed, please select "other". CETI Advanced Cancer Exercise Specialist EX-MED Cancer PD

If you have not completed any exercise oncology Exercise Oncology at ECU Professional Development specific courses, please select "I have not completed course

exercise oncology specific additional training" Grad Cert Exercise Medicine (Oncology) at ECU

Maple Tree Exercise Oncology Instructor

MedFit Education Foundation: Essentials of Cancer Exercise

NFPT Cancer Recovery Specialist

PORI Oncology Rehabilitation Certification

Pinc and Steele Certified Cancer Rehabilitation Physiotherapist/Occupational Therapist

Pink Ribbon Program Breast Cancer Exercise Specialist

ReVital Cancer Rehabilitation Program Specialist Thrive Health Cancer and Exercise Training for

Health and Fitness Professionals

University of Northern Colorado L1 Clinical Cancer Exercise Specialist

University of Northern Colorado L2 Clinical Cancer Exercise Specialist

Yoga4Cancer Other

I have not completed exercise oncology specific additional training

Please specify name of certifying organization

Please specify name of licence/certification

**Personal Reflections for Exercise Oncology Professionals**

**In this section of the survey we want to understand what knowledge and skills you think exercise oncology professionals need to successfully work with people who are undergoing cancer treatment.**

**Please remember the following definition when completing the survey:**

**Exercise oncology professional: a person responsible for developing and/or supervising exercise programming for people with cancer as they undergo treatment.**

From your perspective, what does an exercise oncology professional need to KNOW to effectively work with

patients undergoing cancer treatment? List at least five. Please number each one for clarity.

From your perspective, what does an exercise oncology professional need to be able to DO to effectively work

with patients undergoing cancer treatment. List at least five. Please number each one for clarity.

What competencies/skills would make you feel comfortable referring a patient to an exercise

oncology professional?

What competencies do you think are important for an exercise oncology professional to have to effectively

integrate with an oncology care team?

What was important about an exercise oncology professional for you to feel comfortable working with

them during treatment?

Please provide any additional comments about the knowledge and skills you think are important for an

exercise oncology professional to have to work with people undergoing cancer treatment?

Do you think certifications (beyond university Yes

training) are necessary for exercise oncology No

professionals to demonstrate their specialized ability I don't know/Not sure to work with people with cancer?

**The next section displays the current knowledge, skills, and competencies required of exercise oncology professionals who earn certifications considered to be "gold standard" in the field.**

**PLEASE NOTE: This section of the survey contains nine categories that contain between 4 to 21 specific descriptions of current skills and required areas of knowledge or an exercise professional to earn the certification.**

**This section will take approximately 30 minutes to complete. You can start and stop if you need.**

**For each specific description, you will be asked to rank how important you think it is for an exercise oncology professional to be knowledgeable about this topic or be able to perform/have this skill. You will then be asked to consider the category as a whole and suggest any modifications you would make.**

-

**Rate how important each knowledge/skill/competency is for an exercise oncology professional**

**working with people undergoing cancer treatment.**

**CATEGORY 1: EXERCISE PHYSIOLOGY AND RELATED EXERCISE SCIENCE**

Not Important

At All

Of Little

Importance

Of Average

Importance

Very

Important

Absolutely

Essential

I'm Not Sure

Knowledge of physiologic outcomes that may be improved by exercise training among cancer survivors.

Knowledge of symptoms and psychological attributes that may be improved by exercise training among cancer survivors.

Knowledge of lymph, immunologic, cardiac, neurologic, endocrine and hematologic systems as they pertain to cancer specific exercise issues.

Knowledge of acute and chronic effects of exercise on

temperature regulation and the adverse thermoregulatory/vasomotor symptoms (e.g. hot flashes) experienced by many cancer survivors.

Knowledge of cancer diagnosis and treatment effects on physiological response to acute and chronic exercise, particularly with regard to physical deconditioning, body composition changes, and range of motion.

In your opinion, should "EXERCISE PHYSIOLOGY AND Yes RELATED SCIENCE" be a required category of knowledge No for exercise oncology professionals?

If "No", why not?

Is there anything that is not captured from the list

of proposed knowledge, skills, and competencies within

the category of "EXERCISE PHYSIOLOGY AND RELATED SCIENCE"?

**Rate how important each knowledge/skill/competency is for an exercise oncology professional**

**working with people undergoing cancer treatment.**

**CATEGORY 2: HEALTH APPRAISAL, FITNESS AND CLINICAL EXERCISE TESTING**

Not Important

At All

Of Little

Importance

Of Average

Importance

Very

Important

Absolutely

Essential

I'm Not Sure

Ability to obtain a basic history regarding cancer diagnosis (e.g., type, stage) and treatment (e.g., surgeries, systemic and targeted therapies).

Knowledge of and the ability to recognize the adverse acute, chronic, and late-effects of cancer treatments.

Ability to obtain medical history for other health conditions (e.g. neurological, cardiovascular, musculoskeletal, pulmonary)

that may co-occur and interact with adverse effects of cancer treatments.

Knowledge of and ability to discuss physiologic systems affected by cancer and treatment and how this would affect the major components of fitness, including balance, agility, speed, flexibility, endurance, and strength.

Knowledge of how cancer and its treatments may alter balance,

agility, speed, flexibility, endurance, and strength in cancer survivors and ability to select/modify and interpret tests of these fitness elements.

Knowledge of how cancer and its treatments may affect body

composition in cancer survivors and ability to select/modify and interpret tests of body composition in cancer survivors.

Knowledge of categories of patients that require medical clearance prior to testing or exercise prescription.

Knowledge of cancer-specific relative and absolute contraindications to exercise testing.

How to assess, interpret and record a client's baseline parameters within the categories of cardio-respiratory endurance, muscular strength and endurance, flexibility, range of motion, balance, body composition based on their physical and psychological parameters related to their cancer but also considering other associated medical conditions such as diabetes, anxiety, depression, hypertension, arthritis, osteoporosis, cardiac disease which may be associated with cancer treatments.

Individual risk stratification using recognized guidelines.

In your opinion, should "HEALTH APPRAISAL, FITNESS AND Yes CLINICAL EXERCISE TESTING" be a required category of No knowledge for exercise oncology professionals?Do you

have any additional comments about the category ?

If "No", why not?

Is there anything that is not captured from the list

of proposed knowledge, skills, and competencies within

the category of "HEALTH APPRAISAL, FITNESS AND CLINICAL TESTING"?

**Rate how important each knowledge/skill/competency is for an exercise oncology professional**

**working with people undergoing cancer treatment.**

**CATEGORY 3: EXERCISE PRESCRIPTION AND PROGRAMMING**

Not Important

At All

Of Little

Importance

Of Average

Importance

Very

Important

Absolutely

Essential

I'm Not Sure

Knowledge of current guidelines for exercise in cancer survivors.

Ability to describe benefits and risks of exercise training in the cancer survivor.

Ability to recognize relative and absolute contraindications for

starting or resuming an exercise program, and knowledge of when it is necessary to refer participant back to an appropriate care provider or when they are eligible for referral to community-based exercise programs.

Knowledge of potential for overtraining with the cancer survivor.

How to design an individualized exercise program based on the

initial assessment.

How to determine which baseline parameters can be monitored during the forthcoming exercise program in order to assess ongoing effectiveness and if necessary modify the program and offer alternative exercises.

How to ensure carers/caregivers are comfortable with the

principles of the exercise prescription.

The important general lifestyle factors after cancer and the ability to signpost clients to suitable written materials regarding weight control, adequate protein intake relevant to the level of exercise, healthy and unhealthy diets, supplements, smoking, sun exposure, carcinogens and environmental pollutants.

Knowledge of and ability to use appropriate sun protection for

outdoor programming.

Knowledge, skill and ability to undertake appropriate ongoing screening in order to detect a change in condition and modify exercise prescription/program based on a current medical condition

Knowledge, skill and ability to undertake appropriate ongoing screening in order to detect a change in condition and modify exercise prescription/program based on time since diagnosis on or off adjuvant treatment

Knowledge, skill and ability to undertake appropriate ongoing screening in order to detect a change in condition and modify exercise prescription/program based on type of current therapies (e.g. no swimming during radiation)

Knowledge, skill and ability to undertake appropriate ongoing screening in order to detect a change in condition and modify exercise prescription/program based on type and recency of surgical procedures (e.g., curative or reconstructive)

Knowledge, skill and ability to undertake appropriate ongoing screening in order to detect a change in condition and modify exercise prescription/program based on range of motion

Knowledge, skill and ability to undertake appropriate ongoing screening in order to detect a change in condition and modify exercise prescription/program based on presence of implants

Knowledge, skill and ability to undertake appropriate ongoing screening in order to detect a change in condition and modify exercise prescription/program based on amputations/fusions

Knowledge, skill and ability to undertake appropriate ongoing screening in order to detect a change in condition and modify exercise prescription/program based on effects of treatment on all elements of fitness (agility, speed, coordination, flexibility, strength, and endurance)

Knowledge, skill and ability to undertake appropriate ongoing screening in order to detect a change in condition and modify exercise prescription/program based on hematologic considerations (e.g. anemia, neutropenia)

Knowledge, skill and ability to undertake appropriate ongoing screening in order to detect a change in condition and modify exercise prescription/program based on presence of a central line (PIC or Port)

Knowledge, skill and ability to undertake appropriate ongoing screening in order to detect a change in condition and modify exercise prescription/program based on current adverse effects of treatment, both acute and chronic

Knowledge, skill and ability to undertake appropriate ongoing screening in order to detect a change in condition and modify exercise prescription/program based on individuals that may be at increased risk for adverse late effects that could increase risks associated with exercise (e.g., heart failure)

In your opinion, should "EXERCISE PRESCRIPTION AND Yes PROGRAMMING" be a required category of knowledge for No exercise oncology professionals?

If "No", why not? Yes

No

Is there anything that is not captured from the list

of proposed knowledge, skills, and competencies within

the category of "EXERCISE PRESCRIPTION AND PROGRAMMING"?

**Rate how important each knowledge/skill/competency is for an exercise oncology professional**

**working with people undergoing cancer treatment.**

**CATEGORY 4: NUTRITION & WEIGHT MANAGEMENT**

Not Important

At All

Of Little

Importance

Of Average

Importance

Very

Important

Absolutely

Essential

I'm Not Sure

Knowledge of common effects of cancer treatment on energy

balance and body composition for individuals with

non-metastatic disease.

Knowledge of effects of cancer cachexia on energy balance, intake, and activity level among individuals with metastatic disease.

Knowledge of relationship between body composition as a risk factor for the development of some cancers, and possibly as a risk factor for cancer recurrence.

Knowledge that many cancer survivors may use complementary and alternative medicine (CAM) approaches, and of the potential for these remedies to influence exercise testing and prescription parameters.

Ability to identify unintentional weight change that may relate to disease status and recommend that the client seek appropriate medical attention.

Knowledge of effect of chemotherapy and radiation on the mouth and gastrointestinal system, and the result of these changes on appetite, and food preferences and choices.

Ability to discern when a participant's nutritional questions or status would be best managed by referral to a registered dietitian.

Knowledge of current nutrition guidelines during and after cancer treatment.

Knowledge of hydration needs specific to cancer patients and survivors.

Knowledge of safety of weight loss programs for cancer survivors.

In your opinion, should "NUTRITION & WEIGHT Yes MANAGEMENT" be a required category of knowledge for No exercise oncology professionals?

If "No", why not?

Is there anything that is not captured from the list

of proposed knowledge, skills, and competencies within

the category of "NUTRITION & WEIGHT MANAGEMENT"?

**Rate how important each knowledge/skill/competency is for an exercise oncology professional**

**working with people undergoing cancer treatment.**

**CATEGORY 5: HUMAN BEHAVIOR AND COUNSELING**

Not Important

At All

Of Little

Importance

Of Average

Importance

Very

Important

Absolutely

Essential

I'm Not Sure

Knowledge to identify a teachable moment for cancer survivors and ability to use that time to provide appropriate information and education about resuming or adopting an exercise program.

General knowledge of

psycho-social problems common to cancer survivors, such as depression, anxiety, fear of recurrence, sleep disturbances, body image, sexual dysfunction, and work and marital difficulties.

Knowledge of behavioral strategies that can enhance motivation and adherence (e.g. goal setting, exercise logs, planning).

Knowledge of the impact of cancer diagnosis and treatment on quality of life (QOL), and the potential for exercise to enhance a range of QOL outcomes for survivors (e.g. sleep, fatigue, and other factors).

Knowledge of and ability to determine effectiveness of group exercise programming vs. individual exercise to meet client's needs.

Knowledge of how cancer and cancer treatment relate to ability and readiness to start an exercise program.

Ability to facilitate the social support needs that are cancer specific including connections to websites and local support groups.

Demonstrate communication skills and compassion for patients/clients who have suffered the physical and psychological trauma of cancer and its management.

In your opinion, should "HUMAN BEHAVIOR AND Yes COUNSELING" be a required category of knowledge for No exercise oncology professionals?

In "No", why not?

Is there anything that is not captured from the list

of proposed knowledge, skills, and competencies within

the category of "HUMAN BEHAVIOR AND COUNSELING"?

**Rate how important each knowledge/skill/competency is for an exercise oncology professional**

**working with people undergoing cancer treatment.**

**CATEGORY 6: SAFETY, INJURY PREVENTION, AND EMERGENCY PROCEDURES**

Not Important

At All

Of Little

Importance

Of Average

Importance

Very

Important

Absolutely

Essential

I'm Not Sure

Knowledge of and ability to recognize and respond to cancer-specific safety issues, such as: susceptibility to infection, musculoskeletal and orthopedic changes, unilateral edema, fatigue, lymphedema, neurological changes, osteoporosis, cognitive decline associated with treatment.

Knowledge of and ability to respond to cancer specific emergencies, including: sudden loss of limb function, fever in immune-incompetent patient, and mental status changes.

Knowledge of and ability to respond to the signs and symptoms of new onset and major life threatening complications of cancer, such as superior vena cava syndrome (SVCS), sepsis or infection, and spinal cord compression.

Knowledge of and ability to write-up incident documentation related to cancer specific adverse events.

In your opinion, should "SAFETY, INJURY PREVENTION, Yes AND EMERGENCY PROCEDURES" be a required category of No knowledge for exercise oncology professionals?

If "No", why not?

Is there anything that is not captured from the list

of proposed knowledge, skills, and competencies within

the category of "SAFETY, INJURY PREVENTION, AND EMERGENCY PROCEDURES"?

**Rate how important each knowledge/skill/competency is for an exercise oncology professional**

**working with people undergoing cancer treatment.**

**CATEGORY 7: PROGRAM ADMINISTRATION, QUALITY ASSURANCE & OUTCOME ASSESSMENT**

Not Important

At All

Of Little

Importance

Of Average

Importance

Very

Important

Absolutely

Essential

I'm Not Sure

Knowledge of role in administration and program management within a cancer center, cancer treatment facility, and outpatient setting.

Knowledge of the types of exercise resources and programs available nationally and in the local community and which of these programs cater specifically to the needs of cancer survivors.

Knowledge of and ability to implement effective, professional business practices and ethical promotion of personal training services to the cancer care community (e.g. physicians, nurses, social workers, physical therapists, survivors and their families).

Knowledge of the patient privacy standards and ability to

implement systems to ensure confidentiality of cancer related protected health information of participants.

Knowledge and ability to obtain referral from physician and

communicate with physician about adverse events, abilities and limitations of survivor, and outcomes of testing and training.

Ability to recommend appropriate websites and refer to other health professionals.

Knowledge of reimbursement programs as eligible/available.

Relevant medical/legal issues.

How to establish a safe and stimulating activity environment sensitive to the physical and psychological, confidentially needs of patients/clients with cancer including the appropriateness of group or individual therapies.

The management, evaluation and reporting of information, in verbal and written formats.

In your opinion, should "PROGRAM ADMINISTRATION, Yes QUALITY ASSURANCE & OUTCOME ASSESSMENT" be a required No

category of knowledge for exercise oncology professionals?

If "No", why not?

Is there anything that is not captured from the list

of proposed knowledge, skills, and competencies within

the category of "PROGRAM ADMINISTRATION, QUALITY ASSURANCE & OUTCOME ASSESSMENT"?

**Rate how important each knowledge/skill/competency is for an exercise oncology professional**

**working with people undergoing cancer treatment.**

**CATEGORY 8: CLINICAL AND MEDICAL CONSIDERATIONS**

Not Important

At All

Of Little

Importance

Of Average

Importance

Very

Important

Absolutely

Essential

I'm Not Sure

Knowledge of the major

long-term effects of treatment among childhood cancer survivors that may require careful screening and program adaptation for these individuals.

Knowledge of the common side effects and symptoms of typical

cancer treatments (surgeries, chemotherapy, radiation, hormone manipulations, other drugs).

Knowledge that cancer treatment may accelerate functional decline associated with aging, particularly in the elderly, and that exercise programming may need to be adjusted accordingly.

Knowledge of the combined effects of aging and

cancer-treatment on exercise capacity and selection of appropriate testing modalities and interpretation of results.

Knowledge of the common sites of metastases and ability to

design and implement appropriate exercise programs consistent with this knowledge.

Knowledge of the signs and symptoms associated with new onset lymphedema, and the major cancer types associated with increased lymphedema risk (e.g. breast, head and neck cancer).

Knowledge of lymphedema risk reduction practices, and exercise guidelines.

Knowledge of how cancer treatment may alter cardiovascular risk factors, and inappropriate cardiovascular responses to exercise testing or training.

Knowledge of lymphatic, neurological and immune system factors in cancer survivors that may require further evaluation by medical or allied health professionals before participation in physical activity.

Knowledge of how common cancer treatments affects the ability of cancer survivors to perform exercise, and how to adjust programs accordingly.

Knowledge of the effect of cancer treatment on balance and mobility and the ability to develop an appropriate exercise program that minimizes fall/injury risk.

Knowledge and ability to recognize the limits in the scope of practice for exercise professionals in working with cancer survivors with complex medical issues.

The structure of cancer services and the roles of different

professionals involved in the care of the patient at the various stages in their management pathway.

In your opinion, should "CLINICAL AND MEDICAL Yes CONSIDERATIONS" be a required category of knowledge No for exercise oncology professionals?

If "No", why not?

Is there anything that is not captured from the list

of proposed knowledge, skills, and competencies within

the category of "CLINICAL AND MEDICAL CONSIDERATIONS"?

**Rate how important each knowledge/skill/competency is for an exercise oncology professional**

**working with people undergoing cancer treatment.**

**CATEGORY 9: PHYSIOLOGY, DIAGNOSIS & TREATMENT**

Not Important

At All

Of Little

Importance

Of Average

Importance

Very

Important

Absolutely

Essential

I'm Not Sure

Knowledge of currently accepted screening practices for

surveillance of recurrence for common cancers (e.g. mammography, colonoscopy, prostate specific antigen, pap smears).

Knowledge of the pathology tests used to diagnose common cancers (e.g. biopsy, imaging technologies, and blood tests for tumor markers).

Knowledge of how to communicate effectively with the major medical specialties with whom cancer survivors may interact, including surgery, medical oncology, radiology, dietitians, and psychologists/psychiatrists.

Knowledge of the most common warning signs of recurrence for

common cancers, and when to recommend that clients seek additional medical evaluation.

Understand typical durations of cancer therapy for the major

cancers (breast, prostate, melanoma, ovary, lung, colon), and that therapies are continually evolving/changing.

General knowledge of current cancer treatment strategies, including surgery, systemic therapies (e.g. chemotherapy) and targeted therapies (e.g, anti-angiogenesis inhibitors).

Knowledge of how lifestyle factors, including nutrition, physical activity, and heredity, influence hypothesized mechanisms of cancer etiology, reduce the risk of relapse after initial treatments, and improve long-term survival.

Knowledge of relationship between body composition as a risk factor for the development of some cancers, and possibly as a risk factor for cancer recurrence.

General knowledge of the descriptive epidemiology of cancer, including the prevalence, incidence, and survival statistics for the major cancer types.

General knowledge of cancer biology (e.g., initiation, promotion/progression, and metastases), particularly for the four most common cancers: lung, breast, colon, and prostate.

The environmental/risk factors that can cause cancer and the factors which help our bodies defend against it.

In your opinion, should "PHYSIOLOGY, DIAGNOSIS & Yes

TREATMENT" be a required category of knowledge for No exercise oncology professionals?

If "No", why not?

Is there anything that is not captured from the list

of proposed knowledge, skills, and competencies within

the category of "PHYSIOLOGY, DIAGNOSIS & TREATMENT"?

**These final questions ask you to reflect on all of the categories you have just reviewed as a**

**whole. Please consider any final comments you would like to make.**

Are there any categories of knowledge that should be Yes

added to the current list: No

1. Exercise physiology and related exercise science
2. Health appraisal, fitness, and clinical testing
3. Exercise prescription and programming
4. Nutrition and weight management
5. Human behavior and counselling
6. Safety, injury prevention, and emergency procedures
7. Program administration, quality assurance, and outcome assessment
8. Clinical and medical considerations
9. Physiology, diagnosis, and treatment

If "Yes", what should be added?

Is there anything else you want to add about the knowledge and skills you think are important for an exercise oncology professional to have to work with people undergoing cancer treatment?

Do you think certifications beyond university training Yes

are necessary for exercise oncology professionals to No demonstrate their specialized ability to work with

people with cancer?

# Exercise Oncology Workforce Delphi

These are your survey instructions that you would enter for your survey participants. You may put whatever text you like here, which may include information about the purpose of the survey, who is taking the survey, or how to take the survey.

Surveys can use a single survey link for all respondents, which can be posted on a webpage or emailed out from your email application of choice. By default, all survey responses are collected anonymously (that is, unless your survey asks for name, email, or other identifying information). If you wish to track individuals who have taken your survey, you may upload a list of email addresses into a Participant List within REDCap, in which you can have REDCap send them an email invitation, which will track if they have taken the survey and when it was taken. This method still collects responses anonymously, but if you wish to identify an individual respondent's answers, you may do so by also providing an Identifier in your Participant List. Of course, in that case you may want to inform your respondents in your survey's instructions that their responses are not being collected anonymously and can thus be traced back to them.

Exercise oncology workforce development: A Delphi I agree to proceed

study I do not wish to participate

We are conducting a Delphi study to reach expert consensus on the knowledge, skills, and competencies required for exercise oncology professionals to work with people with cancer in an oncology setting.

**Demographic and Professional Information**

**The following section asks questions about you, your professional background, and your experience with exercise oncology.**

What is your name?

What is your email address?

What is your age? < 30

31 - 40

41 - 50

51 - 60

61 - 70

71+

What is your sex? Male

Female Other

Prefer not to say

What is the highest level of education you have High school or equivalent

completed? Associate's degree

Bachelor's degree Master's degree Professional degree Doctoral degree Other

Please specify what type of doctoral degree (e.g. PhD, MD, DPT)

Please specify which professional degree (e.g. MBA, JD)

Please specify "other"

What best describes your racial/ethnic background? American Indian or Alaskan Native

Aboriginal or Torres Strait Islander Asian or Pacific Islander

Black (not of Hispanic origin) White (not of Hispanic origin) Hispanic/Latino

Other

Please specify "Other"

What stakeholder group do you represent? Exercise oncology professional

Clinician who refers patients to exercise oncology programs

Cancer survivor

What type of exercise professional are you? Exercise physiologist/Exercise scientist Physiotherapist/Physical Therapist Other

Please specify "Other"

What type of clinician are you? General/Primary Care Practitioner Surgical oncologist

Medical oncologist Radiation oncologist Nurse practitioner Registered nurse Physiatrist

Other

Please specify "Other"

What type of cancer have you been diagnosed with? Breast Prostate Bladder Colon Endometrial Espophageal Kidney Stomach Other

Please specify "Other"

What types of treatment have you undertaken or are Surgery

currently undertaking? Check all that apply. Chemotherapy Radiation

Hormone therapy

At what stage(s) did you participate in exercise Before surgery

during treatment? Check all that apply. During chemotherapy During radiotherapy

During hormone therapy

What cancer care organization do you represent?

Who referred you to take part in this survey?

What is the total number of years you have worked as 0 - 5 years an oncology professional? 6 - 10 years

11 - 15 years

16 - 20 years

20+ years Not applicable

In the last year, what percentage of your patient < 25%

population had a cancer diagnosis? 26 - 50% 51 - 75%

76 - 99%

100%

Of the patients with cancer you saw in the last year,

what percentage were curative? 0 50 100

*(Place a mark on the scale above)*

Of the patients with cancer you saw in the last year,

what percentage were palliative? 0 50 100

*(Place a mark on the scale above)*

Of the patients you saw with cancer in the last year, Bladder what were the three most common types you treated? Breast

Please select three choices. Colon and Rectal Endometrial

Kidney

Leukemia (all types)

Liver and Intrahepatic Bile Duct Lung (including Bronchus)

Melanoma

Non-Hodgkin Lymphoma Pancreas

Prostate Thyroid Other

Please specify "Other"

In the last year, on average, how many new patients 0 - 5

with a cancer diagnosis did you work with each month? 6 - 10

11 - 20

21 - 25

26 - 30

30+

In the last year, on average, how many new patients < 25% with a cancer diagnosis did you refer to exercise each 26 - 50% month? 51 - 75%

76 - 99%

100%

Do you have any specific considerations when referring I only refer people with specific diagnoses patients to exercise? Please select all that apply. I only refer people of a certain age range

I only refer people who have previously been active I refer most patients to exercise unless there is

a clear contraindication Other

Please specify what patient diagnoses you refer to

exercise (e.g. breast only, curative only, etc.)

Please specify what age range you refer to exercise

(e.g. under 80 years only.)

Please specify other with regard to what

considerations you make regarding the types of patients you refer to exercise.

What is the age range of the patients you routinely Less than 21 years of age work with? Check all that apply. 21 - 65 years of age

65+ years of age

**Have you completed any additional training/professional development or hold licenses/certification in exercise oncology? Please select "yes" to indicate which of the following courses you have completed.**

**If yours is not listed, please select "other".**

**If you have not completed any exercise oncology specific courses, please select "I have not completed exercise oncology specific additional training"**

Yes

ACSM Certified Cancer Exercise Trainer

ACE Cancer Exercise Specialist

CanRehab Cancer and Exercise Rehabilitation Level 4 Qualification

CETI Cancer Exercise Specialist

CETI Advanced Cancer Exercise Specialist

EX-MED Cancer PD

Exercise Oncology at ECU Professional Development course

Grad Cert Exercise Medicine

(Oncology) at ECU

Maple Tree Exercise Oncology Instructor

MedFit Education Foundation: Essentials of Cancer Exercise

NFPT Cancer Recovery Specialist

PORI Oncology Rehabilitation Certification

Pinc and Steele Certified Cancer Rehabilitation Physiotherapist/Occupational Therapist

Pink Ribbon Program Breast Cancer Exercise Specialist

ReVital Cancer Rehabilitation Program Specialist

Thrive Health Cancer and Exercise Training for Health and Fitness Professionals

University of Northern Colorado L1 Clinical Cancer Exercise Specialist

University of Northern Colorado L2 Clinical Cancer Exercise Specialist

Yoga4Cancer Other

I have not completed exercise oncology specific additional training

Please specify name of certifying organization

Please specify name of licence/certification

**Personal Reflections for Exercise Oncology Professionals**

**In this section of the survey we want to understand what knowledge, skills, and competencies you feel are required of exercise oncology professionals to successfully work with people who are undergoing cancer treatment.**

**Please keep the following definitions in mind when completing the survey:**

**Exercise oncology professional: a person responsible for developing and/or supervising exercise programming for people with cancer as they undergo treatment.**

**Knowledge: topic areas the exercise oncology professional has a theoretical and/or practical understanding of in order to do their job.**

**Skills: the specific tasks an exercise oncology professional should be able to perform effectively in their role.**

**Competencies: the combination of knowledge and skills that allow an exercise oncology professional to perform their role effectively.**

List at least five areas of knowledge you think a clinical exercise oncology professional needs to have

in their role. In other words, what information do you want the exercise oncology professional to have a deep

understanding of?

List at least five skills you think a clinical

exercise oncology professional should have in their

role. In other words, what do you want the exercise oncology professional to be able to do well in their

role? Can be clinical (e.g., conduct exercise tests) or professional (e.g., manage a clinic).

List at least five competencies you think an exercise oncology professional should have in their role. In

other words, how does the exercise oncology professional need to perform their role (e.g.,

performs well under pressure, manages time well)?

List the minimum professional training (e.g., degrees, certifications, internships/practicum) you consider

necessary for a clinical exercise oncology professional. If you don't know or don't have an

opinion, write "Not sure".

**blah blah blah**

The next section displays the current knowledge, skills, and competencies required of exercise oncology professionals who earn certifications considered to be "gold standard" in the field. There are nine categories that contain between 4 to 13 specific descriptions. For each specific description, you will first be asked to rank how important you think it is for an exercise oncology to be knowledgeable about this topic or be able to perform/have this skill. Next, you will be presented with the same list and you will be asked to rank how frequently you use that specific knowledge area or skill/competency in practice. Finally, you will be asked to consider the category as a whole and suggest any modifications you would make.

Please take your time. This section will take approximately 30 minutes to complete. You can start and stop if you need.

This information will be critical in helping us.

**CATEGORY: EXERCISE PHYSIOLOGY AND RELATED EXERCISE SCIENCE**

**Rate how important each knowledge/skill/competency is for an exercise oncology professional working with people undergoing cancer treatment.**

Not Important At All Of Little Importance Very Important Absolutely Essential

Knowledge of physiologic outcomes that may be improved by exercise training among cancer survivors.

Knowledge of symptoms and psychological attributes that may be improved by exercise training among cancer survivors.

Knowledge of lymph, immunologic, cardiac, neurologic, endocrine and hematologic systems as they pertain to cancer specific exercise issues.

Knowledge of acute and chronic effects of exercise on temperature regulation and the adverse thermoregulatory/vasomotor symptoms (e.g. hot flashes) experienced by many cancer survivors.

Knowledge of cancer diagnosis and treatment effects on physiological response to acute and chronic exercise, particularly with regard to physical deconditioning, body composition changes, and range of motion.

**CATEGORY: EXERCISE PHYSIOLOGY AND RELATED EXERCISE SCIENCE**

**Rate how frequently you use each knowledge/skill/competency in your professional role working with people undergoing cancer treatment.**

Knowledge of physiologic outcomes that may be improved by exercise training among cancer survivors.

Never Infrequently

(monthly)

Frequently (weekly) Very frequently

(daily)

Knowledge of symptoms and psychological attributes that may be improved by exercise training among cancer survivors.

Knowledge of lymph, immunologic, cardiac, neurologic, endocrine and hematologic systems as they pertain to cancer specific exercise issues.

Knowledge of acute and chronic effects of exercise on temperature regulation and the adverse thermoregulatory/vasomotor symptoms (e.g. hot flashes) experienced by many cancer survivors.

Knowledge of cancer diagnosis and treatment effects on physiological response to acute and chronic exercise, particularly with regard to physical deconditioning, body composition changes, and range of motion.

In your opinion, should "EXERCISE PHYSIOLOGY AND Yes RELATED SCIENCE" be a required category of knowledge No for exercise oncology professionals?

If "No", why not?

Is there anything that is not captured from list of proposed knowledge, skills, and competencies within

the category of "EXERCISE PHYSIOLOGY AND RELATED SCIENCE"?

**CATEGORY: HEALTH APPRAISAL, FITNESS AND CLINICAL EXERCISE TESTING**

**Rate how important each knowledge/skill/competency is for an exercise oncology professional working with people undergoing cancer treatment.**

Not Important At All Of Little Importance Very Important Absolutely Essential

Ability to obtain a basic history regarding cancer diagnosis (e.g., type, stage) and treatment (e.g., surgeries, systemic and targeted therapies).

Knowledge of and the ability to recognize the adverse acute, chronic, and late-effects of cancer treatments.

Ability to obtain medical history for other health conditions (e.g. neurological, cardiovascular, musculoskeletal, pulmonary) that may co-occur and interact with adverse effects of cancer treatments.

Knowledge of and ability to discuss physiologic systems affected by cancer and treatment and how this would affect the major components of fitness, including balance, agility, speed, flexibility, endurance, and strength.

Knowledge of how cancer and its treatments may alter balance, agility, speed, flexibility, endurance, and strength in cancer survivors and ability to select/modify and interpret tests of these fitness elements.

Knowledge of how cancer and its treatments may affect body composition in cancer survivors and ability to select/modify and interpret tests of body composition in cancer survivors.

Knowledge of categories of patients that require medical clearance prior to testing or exercise prescription.

Knowledge of cancer-specific relative and absolute contraindications to exercise testing.

How to assess, interpret and record a client's baseline parameters within the categories of cardio-respiratory endurance, muscular strength and endurance, flexibility, range of motion, balance, body composition based on their physical and psychological parameters related to their cancer but also considering other associated medical conditions such as diabetes, anxiety, depression, hypertension, arthritis, osteoporosis, cardiac disease which may be associated with cancer treatments.

Individual risk stratification using recognized guidelines.

**CATEGORY: HEALTH APPRAISAL, FITNESS AND CLINICAL EXERCISE TESTING**

**Rate how frequently you use each knowledge/skill/competency in your professional role working with people undergoing cancer treatment.**

Ability to obtain a basic history regarding cancer diagnosis (e.g., type, stage) and treatment (e.g., surgeries, systemic and targeted therapies).

Never Infrequently

(monthly)

Frequently (weekly) Very frequently

(daily)

Knowledge of and the ability to recognize the adverse acute, chronic, and late-effects of cancer treatments.

Ability to obtain medical history for other health conditions (e.g. neurological, cardiovascular, musculoskeletal, pulmonary) that may co-occur and interact with adverse effects of cancer treatments.

Knowledge of and ability to discuss physiologic systems affected by cancer and treatment and how this would affect the major components of fitness, including balance, agility, speed, flexibility, endurance, and strength.

Knowledge of how cancer and its treatments may alter balance, agility, speed, flexibility, endurance, and strength in cancer survivors and ability to select/modify and interpret tests of these fitness elements.

Knowledge of how cancer and its treatments may affect body composition in cancer survivors and ability to select/modify and interpret tests of body composition in cancer survivors.

Knowledge of categories of patients that require medical clearance prior to testing or exercise prescription.

Knowledge of cancer-specific relative and absolute contraindications to exercise testing.

How to assess, interpret and record a client's baseline parameters within the categories of cardio-respiratory endurance, muscular strength and endurance, flexibility, range of motion, balance, body composition based on their physical and psychological parameters related to their cancer but also considering other associated medical conditions such as diabetes, anxiety, depression, hypertension, arthritis, osteoporosis, cardiac disease which may be associated with cancer treatments.

Individual risk stratification using recognized guidelines.

Do you have any additional comments about the category "HEALTH APPRAISAL, FITNESS AND CLINICAL EXERCISE

TESTING"? For example, is it a required category of knowledge for exercise oncology professionals? Are

there any additional knowledge, skills, or competencies you would add? Any other changes to suggest?

**CATEGORY: EXERCISE PRESCRIPTION AND PROGRAMMING**

**Rate how important each knowledge/skill/competency is for an exercise oncology professional working with people undergoing cancer treatment.**

Not Important At All Of Little Importance Very Important Absolutely Essential

1. Knowledge of current guidelines for exercise in cancer survivors.

Ability to describe benefits and risks of exercise training in the cancer survivor.

Ability to recognize relative and absolute contraindications for starting or resuming an exercise program, and knowledge of when it is necessary to refer participant back to an appropriate care provider or when they are eligible for referral to community-based exercise programs.

Knowledge, skill and ability to undertake appropriate ongoing screening in order to detect a change in condition and modify exercise prescription/program based on: a. current medical condition ○ b. time since diagnosis on or off adjuvant treatment ○ c. type of current therapies (e.g. no swimming during radiation) ○ d. type and recency of surgical procedures (e.g., curative or reconstructive)

- e. range of motion ○ f. presence of implants ○ g. amputations/fusions ○ h. effects of treatment on all elements of fitness (agility, speed, coordination, flexibility, strength, and endurance) ○ i. hematologic considerations (e.g. anemia, neutropenia) ○ j. presence of a central line (PIC or Port) ○ k. current adverse effects of treatment, both acute and chronic ○ l. individuals that may be at increased risk for adverse late effects that could increase risks associated with exercise (e.g., heart failure)

Knowledge of potential for overtraining with the cancer survivor.

Knowledge of and ability to use appropriate sun protection for outdoor programming.

How to design an individualized exercise program based on the initial assessment.

How to determine which baseline parameters can be monitored during the forthcoming exercise program in order to assess ongoing effectiveness and if necessary modify the program and offer alternative exercises.

How to ensure carers are comfortable with the principles of the exercise prescription.

The important general lifestyle factors after cancer and the ability to signpost clients to suitable written materials regarding weight control, adequate protein intake relevant to the level of exercise, healthy and unhealthy diets, supplements, smoking, sun exposure, carcinogens and environmental pollutants.

**CATEGORY: EXERCISE PRESCRIPTION AND PROGRAMMING**

**Rate how frequently you use each knowledge/skill/competency in your professional role working with people undergoing cancer treatment.**

Knowledge of current guidelines for exercise in cancer survivors.

Ability to describe benefits and risks of exercise training in the cancer survivor.

Never Infrequently

(monthly)

Frequently (weekly) Very frequently

(daily)

Ability to recognize relative and absolute contraindications for starting or resuming an exercise program, and knowledge of when it is necessary to refer participant back to an appropriate care provider or when they are eligible for referral to community-based exercise programs.

Knowledge, skill and ability to undertake appropriate ongoing screening in order to detect a change in condition and modify exercise prescription/program based on: ○ a. current medical condition ○ b. time since diagnosis on or off adjuvant treatment ○ c. type of current therapies (e.g. no swimming during radiation) ○ d. type and recency of surgical procedures (e.g., curative or reconstructive)

- e. range of motion ○ f. presence of implants ○ g. amputations/fusions ○ h. effects of treatment on all elements of fitness (agility, speed, coordination, flexibility, strength, and endurance) ○ i. hematologic considerations (e.g. anemia, neutropenia) ○ j. presence of a central line (PIC or Port) ○ k. current adverse effects of treatment, both acute and chronic ○ l. individuals that may be at increased risk for adverse late effects that could increase risks associated with exercise (e.g., heart failure)

Knowledge of potential for overtraining with the cancer survivor.

Knowledge of and ability to use appropriate sun protection for outdoor programming.

How to design an individualized exercise program based on the initial assessment.

How to determine which baseline parameters can be monitored during the forthcoming exercise program in order to assess ongoing effectiveness and if necessary modify the program and offer alternative exercises.

How to ensure carers are comfortable with the principles of the exercise prescription.

The important general lifestyle factors after cancer and the ability to signpost clients to suitable written materials regarding weight control, adequate protein intake relevant to the level of exercise, healthy and unhealthy diets, supplements, smoking, sun exposure, carcinogens and environmental pollutants.

**CATEGORY: NUTRITION & WEIGHT MANAGEMENT**

**Rate how important each knowledge/skill/competency is for an exercise oncology professional working with people undergoing cancer treatment.**

Not Important At All Of Little Importance Very Important Absolutely Essential

Knowledge of common effects of cancer treatment on energy balance and body composition for individuals with

non-metastatic disease.

Knowledge of effects of cancer cachexia on energy balance, intake, and activity level among individuals with metastatic disease.

Knowledge of relationship between body composition as a risk factor for the development of some cancers, and possibly as a risk factor for cancer recurrence.

Knowledge that many cancer survivors may use complementary and alternative medicine (CAM) approaches, and of the potential for these remedies to influence exercise testing and prescription parameters.

Ability to identify unintentional weight change that may relate to disease status and recommend that the client seek appropriate medical attention.

Knowledge of effect of chemotherapy and radiation on the mouth and gastrointestinal system, and the result of these changes on appetite, and food preferences and choices.

Ability to discern when a participant's nutritional questions or status would be best managed by referral to a registered dietitian.

Knowledge of current nutrition guidelines during and after cancer treatment.

Knowledge of hydration needs specific to cancer patients and survivors.

Knowledge of safety of weight loss programs for cancer survivors.

**CATEGORY: NUTRITION & WEIGHT MANAGEMENT**

**Rate how frequently you use each knowledge/skill/competency in your professional role working with people undergoing cancer treatment.**

Knowledge of common effects of cancer treatment on energy balance and body composition for individuals with

non-metastatic disease.

Never Infrequently

(monthly)

Frequently (weekly) Very frequently

(daily)

Knowledge of effects of cancer cachexia on energy balance, intake, and activity level among individuals with metastatic disease.

Knowledge of relationship between body composition as a risk factor for the development of some cancers, and possibly as a risk factor for cancer recurrence.

Knowledge that many cancer survivors may use complementary and alternative medicine (CAM) approaches, and of the potential for these remedies to influence exercise testing and prescription parameters.

Ability to identify unintentional weight change that may relate to disease status and recommend that the client seek appropriate medical attention.

Knowledge of effect of chemotherapy and radiation on the mouth and gastrointestinal system, and the result of these changes on appetite, and food preferences and choices.

Ability to discern when a participant's nutritional questions or status would be best managed by referral to a registered dietitian.

Knowledge of current nutrition guidelines during and after cancer treatment.

Knowledge of hydration needs specific to cancer patients and survivors.

Knowledge of safety of weight loss programs for cancer survivors.

**CATEGORY: HUMAN BEHAVIOR AND COUNSELING**

**Rate how important each knowledge/skill/competency is for an exercise oncology professional working with people undergoing cancer treatment.**

Not Important At All Of Little Importance Very Important Absolutely Essential

Knowledge to identify a teachable moment for cancer survivors and ability to use that time to provide appropriate information and education about resuming or adopting an exercise program.

General knowledge of

psycho-social problems common to cancer survivors, such as depression, anxiety, fear of recurrence, sleep disturbances, body image, sexual dysfunction, and work and marital difficulties.

Knowledge of behavioral strategies that can enhance motivation and adherence (e.g. goal setting, exercise logs, planning).

Knowledge of the impact of cancer diagnosis and treatment on quality of life (QOL), and the potential for exercise to enhance a range of QOL outcomes for survivors (e.g. sleep, fatigue, and other factors).

Knowledge of and ability to determine effectiveness of group exercise programming vs. individual exercise to meet client's needs.

Knowledge of how cancer and cancer treatment relate to ability and readiness to start an exercise program.

Ability to facilitate the social support needs that are cancer specific including connections to websites and local support groups.

Demonstrate communication skills and compassion for patients/clients who have suffered the physical and psychological trauma of cancer and its management.

**CATEGORY: HUMAN BEHAVIOR AND COUNSELING**

**Rate how frequently you use each knowledge/skill/competency in your professional role working with people undergoing cancer treatment.**

1. Knowledge to identify a teachable moment for cancer survivors and ability to use that time to provide appropriate information and education about resuming or adopting an exercise program.

Never Infrequently

(monthly)

Frequently (weekly) Very frequently

(daily)

1. General knowledge of

psycho-social problems common to cancer survivors, such as depression, anxiety, fear of recurrence, sleep disturbances, body image, sexual dysfunction, and work and marital difficulties.

1. Knowledge of behavioral strategies that can enhance motivation and adherence (e.g. goal setting, exercise logs, planning).
2. Knowledge of the impact of cancer diagnosis and treatment on quality of life (QOL), and the potential for exercise to enhance a range of QOL outcomes for survivors (e.g. sleep, fatigue, and other factors).
3. Knowledge of and ability to determine effectiveness of group exercise programming vs. individual exercise to meet client's needs.
4. Knowledge of how cancer and cancer treatment relate to ability and readiness to start an exercise program.
5. Ability to facilitate the social support needs that are cancer specific including connections to websites and local support groups.
6. Demonstrate communication skills and compassion for patients/clients who have suffered the physical and psychological trauma of cancer and its management.

**CATEGORY: SAFETY, INJURY PREVENTION, AND EMERGENCY PROCEDURES**

**Rate how important each knowledge/skill/competency is for an exercise oncology professional working with people undergoing cancer treatment.**

Not Important At All Of Little Importance Very Important Absolutely Essential

Knowledge of and ability to recognize and respond to cancer-specific safety issues, such as: susceptibility to infection, musculoskeletal and orthopedic changes, unilateral edema, fatigue, lymphedema, neurological changes, osteoporosis, cognitive decline associated with treatment.

Knowledge of and ability to respond to cancer specific emergencies, including: sudden loss of limb function, fever in immune-incompetent patient, and mental status changes.

Knowledge of and ability to respond to the signs and symptoms of new onset and major life threatening complications of cancer, such as superior vena cava syndrome (SVCS), sepsis or infection, and spinal cord compression.

Knowledge of and ability to write-up incident documentation related to cancer specific adverse events.

**CATEGORY: SAFETY, INJURY PREVENTION, AND EMERGENCY PROCEDURES**

**Rate how frequently you use each knowledge/skill/competency in your professional role working with people undergoing cancer treatment.**

Knowledge of and ability to recognize and respond to cancer-specific safety issues, such as: susceptibility to infection, musculoskeletal and orthopedic changes, unilateral edema, fatigue, lymphedema, neurological changes, osteoporosis, cognitive decline associated with treatment.

Never Infrequently

(monthly)

Frequently (weekly) Very frequently

(daily)

Knowledge of and ability to respond to cancer specific emergencies, including: sudden loss of limb function, fever in immune-incompetent patient, and mental status changes.

Knowledge of and ability to respond to the signs and symptoms of new onset and major life threatening complications of cancer, such as superior vena cava syndrome (SVCS), sepsis or infection, and spinal cord compression.

Knowledge of and ability to write-up incident documentation related to cancer specific adverse events.

**CATEGORY: PROGRAM ADMINISTRATION, QUALITY ASSURANCE & OUTCOME ASSESSMENT**

**Rate how important each knowledge/skill/competency is for an exercise oncology professional working with people undergoing cancer treatment.**

Not Important At All Of Little Importance Very Important Absolutely Essential

Knowledge of role in administration and program management within a cancer center, cancer treatment facility, and outpatient setting.

Knowledge of the types of exercise resources and programs available nationally and in the local community and which of these programs cater specifically to the needs of cancer survivors.

Knowledge of and ability to implement effective, professional business practices and ethical promotion of personal training services to the cancer care community (e.g. physicians, nurses, social workers, physical therapists, survivors and their families).

Knowledge of the patient privacy standards and ability to implement systems to ensure confidentiality of cancer related protected health information of participants.

Knowledge and ability to obtain referral from physician and communicate with physician about adverse events, abilities and limitations of survivor, and outcomes of testing and training.

Ability to recommend appropriate websites and refer to other health professionals.

Knowledge of reimbursement programs as eligible/available.

Relevant medico-legal issues.

How to establish a safe and stimulating activity environment sensitive to the physical and psychological, confidentially needs of patients/clients with cancer including the appropriateness of group or individual therapies.

The management, evaluation and reporting of information, in verbal and written formats.

**CATEGORY: PROGRAM ADMINISTRATION, QUALITY ASSURANCE & OUTCOME ASSESSMENT**

**Rate how frequently you use each knowledge/skill/competency in your professional role working with people undergoing cancer treatment.**

Knowledge of role in administration and program management within a cancer center, cancer treatment facility, and outpatient setting.

Never Infrequently

(monthly)

Frequently (weekly) Very frequently

(daily)

Knowledge of the types of exercise resources and programs available nationally and in the local community and which of these programs cater specifically to the needs of cancer survivors.

Knowledge of and ability to implement effective, professional business practices and ethical promotion of personal training services to the cancer care community (e.g. physicians, nurses, social workers, physical therapists, survivors and their families).

Knowledge of the patient privacy standards and ability to implement systems to ensure confidentiality of cancer related protected health information of participants.

Knowledge and ability to obtain referral from physician and communicate with physician about adverse events, abilities and limitations of survivor, and outcomes of testing and training.

Ability to recommend appropriate websites and refer to other health professionals.

Knowledge of reimbursement programs as eligible/available.

Relevant medico-legal issues.

How to establish a safe and stimulating activity environment sensitive to the physical and psychological, confidentially needs of patients/clients with cancer including the appropriateness of group or individual therapies.

The management, evaluation and reporting of information, in verbal and written formats.

**CATEGORY: CLINICAL AND MEDICAL CONSIDERATIONS**

**Rate how important each knowledge/skill/competency is for an exercise oncology professional working with people undergoing cancer treatment.**

Not Important At All Of Little Importance Very Important Absolutely Essential

Knowledge of the major

long-term effects of treatment among childhood cancer survivors that may require careful screening and program adaptation for these individuals.nagement within a cancer center, cancer treatment facility, and outpatient setting.

Knowledge of the common side effects and symptoms of typical cancer treatments (surgeries, chemotherapy, radiation, hormone manipulations, other drugs).

Knowledge that cancer treatment may accelerate functional decline associated with aging, particularly in the elderly, and that exercise programming may need to be adjusted accordingly.

Knowledge of the combined effects of aging and

cancer-treatment on exercise capacity and selection of appropriate testing modalities and interpretation of results.

Knowledge of the common sites of metastases and ability to design and implement appropriate exercise programs consistent with this knowledge.

Knowledge of the signs and symptoms associated with new onset lymphedema, and the major cancer types associated with increased lymphedema risk (e.g. breast, head and neck cancer).

Knowledge of lymphedema risk reduction practices, and exercise guidelines.

Knowledge of how cancer treatment may alter cardiovascular risk factors, and inappropriate cardiovascular responses to exercise testing or training.

Knowledge of lymphatic, neurological and immune system factors in cancer survivors that may require further evaluation by medical or allied health professionals before participation in physical activity.

Knowledge of how common cancer treatments affects the ability of cancer survivors to perform exercise, and how to adjust programs accordingly.

Knowledge of the effect of cancer treatment on balance and mobility and the ability to develop an appropriate exercise program that minimizes fall/injury risk.

Knowledge and ability to recognize the limits in the scope of practice for exercise professionals in working with cancer survivors with complex medical issues.

The structure of cancer services and the roles of different professionals involved in the care of the patient at the various stages in their management pathway.

**CATEGORY: CLINICAL AND MEDICAL CONSIDERATIONS**

**Rate how frequently you use each knowledge/skill/competency in your professional role working with people undergoing cancer treatment.**

1. Knowledge of the major long-term effects of treatment among childhood cancer survivors that may require careful screening and program

adaptation for these individuals.

Never Infrequently

(monthly)

Frequently (weekly) Very frequently

(daily)

1. Knowledge of the common side effects and symptoms of typical cancer treatments (surgeries, chemotherapy, radiation, hormone manipulations, other drugs).
2. Knowledge that cancer treatment may accelerate functional decline associated with aging, particularly in the elderly, and that exercise programming may need to be adjusted accordingly.
3. Knowledge of the combined effects of aging and

cancer-treatment on exercise capacity and selection of appropriate testing modalities and interpretation of results.

1. Knowledge of the common sites of metastases and ability to design and implement appropriate exercise programs consistent with this knowledge.
2. Knowledge of the signs and symptoms associated with new onset lymphedema, and the major cancer types associated with increased lymphedema risk (e.g. breast, head and neck cancer).
3. Knowledge of lymphedema risk reduction practices, and exercise guidelines.
4. Knowledge of how cancer treatment may alter cardiovascular risk factors, and inappropriate cardiovascular responses to exercise testing or training.
5. Knowledge of lymphatic, neurological and immune system factors in cancer survivors that may require further evaluation by medical or allied health professionals before participation in physical activity.
6. Knowledge of how common cancer treatments affects the ability of cancer survivors to perform exercise, and how to adjust programs accordingly.
7. Knowledge of the effect of cancer treatment on balance and mobility and the ability to develop an appropriate exercise program that minimizes fall/injury risk.
8. Knowledge and ability to recognize the limits in the scope of practice for exercise professionals in working with cancer survivors with complex medical issues.
9. The structure of cancer services and the roles of different professionals involved in the care of the patient at the various stages in their management pathway.

**CATEGORY: PHYSIOLOGY, DIAGNOSIS & TREATMENT**

**Rate how important each knowledge/skill/competency is for an exercise oncology professional working with people undergoing cancer treatment.**

Not Important At All Of Little Importance Very Important Absolutely Essential

Knowledge of currently accepted screening practices for surveillance of recurrence for common cancers (e.g. mammography, colonoscopy, prostate specific antigen, pap smears).

Knowledge of the pathology tests used to diagnose common cancers (e.g. biopsy, imaging technologies, and blood tests for tumor markers).

Knowledge of how to communicate effectively with the major medical specialties with whom cancer survivors may interact, including surgery, medical oncology, radiology, dietitians, and psychologists/psychiatrists.

Knowledge of the most common warning signs of recurrence for common cancers, and when to recommend that clients seek additional medical evaluation.

Understand typical durations of cancer therapy for the major cancers (breast, prostate, melanoma, ovary, lung, colon), and that therapies are continually evolving/changing.

General knowledge of current cancer treatment strategies, including surgery, systemic therapies (e.g. chemotherapy) and targeted therapies (e.g, anti-angiogenesis inhibitors).

Knowledge of how lifestyle factors, including nutrition, physical activity, and heredity, influence hypothesized mechanisms of cancer etiology, reduce the risk of relapse after initial treatments, and improve long-term survival.

Knowledge of relationship between body composition as a risk factor for the development of some cancers, and possibly as a risk factor for cancer recurrence.

General knowledge of the descriptive epidemiology of cancer, including the prevalence, incidence, and survival statistics for the major cancer types.

General knowledge of cancer biology (e.g., initiation, promotion/progression, and metastases), particularly for the four most common cancers: lung, breast, colon, and prostate.

The environmental/risk factors that can cause cancer and the factors which help our bodies defend against it.

**CATEGORY: PHYSIOLOGY, DIAGNOSIS & TREATMENT**

**Rate how frequently you use each knowledge/skill/competency in your professional role working with people undergoing cancer treatment.**

Knowledge of currently accepted screening practices for surveillance of recurrence for common cancers (e.g. mammography, colonoscopy, prostate specific antigen, pap smears).

Never Infrequently

(monthly)

Frequently (weekly) Very frequently

(daily)

Knowledge of the pathology tests used to diagnose common cancers (e.g. biopsy, imaging technologies, and blood tests for tumor markers).

Knowledge of how to communicate effectively with the major medical specialties with whom cancer survivors may interact, including surgery, medical oncology, radiology, dietitians, and psychologists/psychiatrists.

Knowledge of the most common warning signs of recurrence for common cancers, and when to recommend that clients seek additional medical evaluation.

Understand typical durations of cancer therapy for the major cancers (breast, prostate, melanoma, ovary, lung, colon), and that therapies are continually evolving/changing.

General knowledge of current cancer treatment strategies, including surgery, systemic therapies (e.g. chemotherapy) and targeted therapies (e.g, anti-angiogenesis inhibitors).

Knowledge of how lifestyle factors, including nutrition, physical activity, and heredity, influence hypothesized mechanisms of cancer etiology, reduce the risk of relapse after initial treatments, and improve long-term survival.

Knowledge of relationship between body composition as a risk factor for the development of some cancers, and possibly as a risk factor for cancer recurrence.

General knowledge of the descriptive epidemiology of cancer, including the prevalence, incidence, and survival statistics for the major cancer types.

General knowledge of cancer biology (e.g., initiation, promotion/progression, and metastases), particularly for the four most common cancers: lung, breast, colon, and prostate.

The environmental/risk factors that can cause cancer and the factors which help our bodies defend against it.
