# Supplementary material for: Identification of core competencies for exercise oncology professionals: A Delphi study of United States and Australian participants
Source: Cancer Med. 2024 Jul 24;13(14):e70004. doi: 10.1002/cam4.70004 (PMC11267632; doi:10.1002/cam4.70004)
Supplement: Supplementary file 1 — Data S1. [file CAM4-13-e70004-s001.zip › S2.Round2 Survey.docx]

Round 2: Exercise Oncology Workforce Survey

Thank you for sharing your time and expertise with us. Your responses will help to define the specialized skillset and training requirements an exercise professional needs to deliver exercise programming to people with cancer and move us closer to the goal of making exercise standard practice in oncology.

Exercise oncology workforce development: A Delphi study (Round 2)

We are conducting a Delphi study to reach expert consensus on the knowledge, skills, and competencies

required for exercise oncology professionals to work with people with cancer in an oncology setting. This is Round 2 of 3.


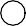
 I agree to proceed
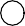
 I do not wish to participate

What is your name (first and last)?

What is your email address?

Round 2: Exercise Oncology Workforce Introduction

The specific results of each category of competencies you ranked in Round 1 of the Delphi study are described in the following sections of the survey. A few comments were made about the competencies overall. Please keep these comments in mind as you complete this round of the survey.

1. Awareness of common misconceptions. The participant stated: “I have found in our practice that so many can safely and effectively push limits when it comes to exercise, to help improve quality of life, muscular strength, endurance, etc. and so often when working with a cancer patient, many are either fearful to do so or treat them as if they are weak and incapable.”
2. Focus on implementation. The participant stated: “I suggest a heavier focus on the implementation pieces. As part of a clinical care team (plus some basic knowledge of how/where to find good clinical information) the biggest gaps and need is related to how to guide a patient to ‘move through’ cancer.”
3. Obtaining referrals. The participant stated: “I think the importance of obtaining referrals can not be underrated. A clinician can have all the knowledge in the world regarding a condition/illness and how to rehabilitate from it but if they are unable to sell the service to a referrer and build an appealing program their knowledge will be wasted.”

Finally, one participant asked for clarification about what is meant by "Exercise Oncology Professional". This survey aims to define the compentcies required for an exercise professional to be able to work with people undergoing active cancer treatment.

**Category 1: Exercise Physiology and Related Science n=29**

**Category agreement**

**There was full agreement that this should be a required category of knowledge for exercise oncology professionals.**

**Notes about category**

**One participant reinforced that it is “important to consider changes in cardiovascular, respiratory, muscular, lymphatic and neurological systems when working with the cancer patient in order to create the best program experience” and thought the items in this category adequately reflected these areas of knowledge.**

**Another participant pointed out that while many exercise professionals receive in-depth education on ECG and cellular/microbiologic information as part of their training, it may not be an essential component as it is not directly related to clinical practice.**

Specific items in category

Five specific knowledge, skills, and abilities (KSAs) were described in this category. The four below reached consensus (>90% agreement) as being a “very important” or “absolutely essential” area of knowledge for an exercise oncology professional:

1. Knowledge of physiologic outcomes that may be improved by exercise training among cancer survivors.
2. Knowledge of symptoms and psychological attributes that may be improved by exercise training among cancer survivors.
3. Knowledge of lymph, immunologic, cardiac, neurologic, endocrine and hematologic systems as they pertain to cancer specific exercise issues.
4. Knowledge of cancer diagnosis and treatment effects on physiological response to acute and chronic exercise, particularly with regard to physical deconditioning, body composition changes, and range of motion.

Do you have any concerns or comments with the statements that reached consensus for inclusion in Category 1: Exercise Physiology and Related Science?

If yes, please detail them in the comment box. If no, continue to next page.

**One KSA did not achieve consensus.**

**Do you think this statement should be included in the final list of competencies for Category 1: Exercise Physiology and Related Science?**

Knowledge of acute and chronic effects of exercise on temperature regulation and the adverse thermoregulatory/vasomotor symptoms (e.g., hot flashes) experienced by many cancer survivors.

(Round 1 results: 72% = very important/absolute essential; 28% = of average importance)
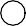
 Yes
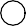
 No

**The following five suggestions were made as potential additions to Category 1: Exercise**

**Physiology and Related Science. Please rank your level of agreement with the importance of each item below.**

1. Add musculoskeletal to the list in this current Absolutely essential statement: Knowledge of lymph, immunologic, cardiac, Very important neurologic, endocrine, and hematologic systems as they Of average importance pertain to cancer specific exercise issues. Of little importance


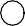

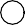

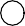

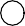

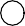


I'm not sure

1. Understand the emerging evidence regarding the Absolutely essential potential effects of exercise on the physiology of Very important

cancer treatment (e.g., accelerated ageing). Of average importance Of little importance


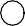

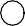

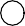

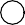

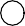


I'm not sure

1. Understand the impact of exercise on Absolutely essential

oncology-related comorbidities, such as Very important


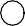

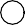

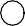

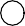

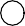


cardiotoxicity, diabetes, etc. Of average importance Of little importance

I'm not sure

1. Understand how exercise can impact cognition and Absolutely essential mental health. Very important

Of average importance Of little importance


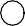

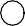

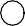

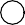

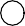


I'm not sure

1. Understand how exercise can assist cancer patients Absolutely essential across the cancer continuum Very important

(diagnosis/treatment/recovery/palliative care). Of average importance Of little importance


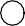

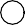

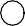

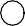

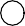


I'm not sure

Reflecting on the results of Round 1 and the category as a whole, do you have any additional comments or suggestions for Category 1: Exercise Physiology and Related Science?

If yes, please detail your comments below. If no, continue to the next page.

**Category 2: Health Appraisal, Fitness, and Clinical Exercise Testing n=29**

**Category agreement**

**Ninety-three percent of participants thought this should be a required category of knowledge for exercise oncology professionals.**

**Notes about category**

**Those that disagreed raised concerns for the following reasons:**

1. **they felt this category is a basic requirement of being a Board Certified Physical Therapist, or**
2. **because much of this information would be generated from the clinical care team, could be found in a patient's medical record, or should involve a partner conversation with the**

**provider.**

Specific items in category

Ten specific knowledge, skills, and abilities (KSAs) were described in this category. The nine below reached consensus (>90% agreement) as being a “very important” or “absolutely essential” area of knowledge for an exercise oncology professional:

1. Ability to obtain a basic history regarding cancer diagnosis (e.g., type, stage) and treatment (e.g., surgeries, systemic and targeted therapies).
2. Knowledge of and the ability to recognize the adverse acute, chronic, and late-effects of cancer treatments.
3. Ability to obtain medical history for other health conditions (e.g. neurological, cardiovascular, musculoskeletal, pulmonary) that may co-occur and interact with adverse effects of cancer treatments.
4. Knowledge of and ability to discuss physiologic systems affected by cancer and treatment and how this would affect the major components of fitness, including balance, agility, speed, flexibility, endurance, and strength.
5. Knowledge of how cancer and its treatments may alter balance, agility, speed, flexibility, endurance, and strength in cancer survivors and ability to select/modify and interpret tests of these fitness elements.
6. Knowledge of how cancer and its treatments may affect body composition in cancer survivors and ability to select/modify and interpret tests of body composition in cancer survivors.
7. Knowledge of categories of patients that require medical clearance prior to testing or exercise prescription.
8. Knowledge of cancer-specific relative and absolute contraindications to exercise testing.
9. How to assess, interpret and record a client's baseline parameters within the categories of cardio-respiratory endurance, muscular strength and endurance, flexibility, range of motion, balance, body composition based on their physical and psychological parameters related to their cancer but also considering other associated medical conditions such as diabetes, anxiety, depression, hypertension, arthritis, osteoporosis, cardiac disease which may be associated with cancer treatments.

Do you have any concerns or comments with the statements that reached consensus for inclusion in Category 2: Health Appraisal, Fitness, and Clinical Testing?

If yes, please detail them in the comment box. If no, continue to the next page.

**One KSA did not achieve consensus.**

**Do you think this statement should be included in the final list of competencies for Category 2: Health Appraisal, Fitness, and Clinical Exercise Testing?**

1. Individual risk stratification using recognized guidelines.

(Round 1 results: 83% = very important/absolute essential; 17% = of average importance)
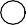
 Yes
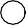
 No

**The following three suggestions were made as potential additions to Category 2: Health**

**Appraisal, Fitness, and Clinical Testing. Please rank your level of agreement with the importance of each item below.**

1. Ability to effectively review medical chart notes Absolutely essential


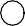

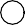

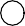

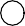

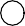


to understand cancer diagnosis and treatments. Very important

Of average importance Of little importance

I'm not sure

1. Ability to perform subjective interview to Absolutely essential

understand patient's goals and patient burden of Very important


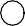

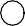

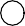

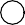

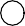


symptoms from cancer or cancer treatment. Of average importance Of little importance

I'm not sure

1. Ability to develop and use appropriate assessment Absolutely essential protocols. Very important

Of average importance Of little importance


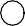

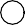

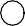

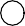

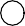


I'm not sure

Reflecting on the results of Round 1 and the category as a whole, do you have any additional comments or suggestions for Category 2: Health Appraisal, Fitness, and Clinical Testing?

If yes, please detail your comments below. If no, continue to the next page.

**Category 3: Exercise Prescription and Programming n=29**

**Category agreement**

**Ninety-seven percent of participants thought this should be a required category of knowledge for exercise oncology professionals.**

**Notes about category**

**Two participants noted the specific items provided a comprehensive list for this category.**

**One participant noted that the list may be “too medical” and raised concerns that some of the guidelines are very limiting to what patients should do and are often more preventative. They gave the example of guidelines for blood counts, noting while you don’t want someone with low platelets to fall and bleed out, the conservative guideline often leaves patients too scared to do anything. Their suggestion was to encourage building an exercise program that would also support clinical judgment. A similar concern was raised by another participant who noted that the “knowledge, skill, and ability to recognize all potential risks and adverse effects can result in very conservative exercise prescriptions” that do not stimulate desired adaptations.**

Specific items in category

Twenty-one specific knowledge, skills, and abilities (KSAs) were described in this category. Eighteen reached consensus (>90% agreement) as being a “very important” or “absolutely essential” area of knowledge for an exercise oncology professional:

1. Knowledge of current guidelines for exercise in cancer survivors.
2. Ability to describe benefits and risks of exercise training in the cancer survivor.
3. Ability to recognize relative and absolute contraindications for starting or resuming an exercise program, and knowledge of when it is necessary to refer participant back to an appropriate care provider or when they are eligible for referral to community-based exercise programs.
4. Knowledge of potential for overtraining with the cancer survivor.
5. How to design an individualized exercise program based on the initial assessment.
6. How to determine which baseline parameters can be monitored during the forthcoming exercise program in order to assess ongoing effectiveness and if necessary modify the program and offer alternative exercises.
7. Knowledge, skill and ability to undertake appropriate ongoing screening in order to detect a change in condition and modify exercise prescription/program based on a current medical condition
8. Knowledge, skill and ability to undertake appropriate ongoing screening in order to detect a change in condition and modify exercise prescription/program based on time since diagnosis on or off adjuvant treatment
9. Knowledge, skill and ability to undertake appropriate ongoing screening in order to detect a change in condition and modify exercise prescription/program based on type of current therapies (e.g. no swimming during radiation)
10. Knowledge, skill and ability to undertake appropriate ongoing screening in order to detect a change in condition and modify exercise prescription/program based on type and recency of surgical procedures (e.g., curative or reconstructive)
11. Knowledge, skill and ability to undertake appropriate ongoing screening in order to detect a change in condition and modify exercise prescription/program based on range of motion
12. Knowledge, skill and ability to undertake appropriate ongoing screening in order to detect a change in condition and modify exercise prescription/program based on presence of implants
13. Knowledge, skill and ability to undertake appropriate ongoing screening in order to detect a change in condition and modify exercise prescription/program based on amputations/fusions
14. Knowledge, skill and ability to undertake appropriate ongoing screening in order to detect a change in condition and modify exercise prescription/program based on effects of treatment on all elements of fitness (agility, speed, coordination, flexibility, strength, and endurance)
15. Knowledge, skill and ability to undertake appropriate ongoing screening in order to detect a change in condition and modify exercise prescription/program based on hematologic considerations (e.g. anemia, neutropenia)
16. Knowledge, skill and ability to undertake appropriate ongoing screening in order to detect a change in condition and modify exercise prescription/program based on presence of a central line (PIC or Port)
17. Knowledge, skill and ability to undertake appropriate ongoing screening in order to detect a change in condition and modify exercise prescription/program based on current adverse effects of treatment, both acute and chronic
18. Knowledge, skill and ability to undertake appropriate ongoing screening in order to detect a change in condition and modify exercise prescription/program based on individuals that may be at increased risk for adverse late effects that could increase risks associated with exercise (e.g., heart failure)

Do you have any concerns or comments with the statements that reached consensus for inclusion in Category 3: Exercise Prescription and Programming?

If yes, please detail them in the comment box. If no, continue to the next page.

**The following three KSAs did not achieve consensus.**

**Do you think these statements should be included in Category 3: Exercise Prescription and Programming?**

1. How to ensure carers/caregivers are comfortable with the principles of the exercise prescription. (Round 1 results: 66% = very important/absolute essential; 34% = of average importance)


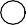
 Yes
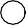
 No

1. The important general lifestyle factors after cancer and the ability to signpost clients to suitable written materials regarding weight control, adequate protein intake relevant to the level of exercise, healthy and unhealthy diets, supplements, smoking, sun exposure, carcinogens and environmental pollutants.

(Round 1 results: 69% = very important/absolute essential; 31% = of average importance)
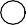
 Yes
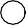
 No

1. Knowledge of and ability to use appropriate sun protection for outdoor programming.

(Round 1 results: 48% = very important/absolute essential; 48% = of average importance; 4% = of little importance)
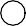
 Yes
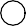
 No

**The following ten suggestions were made as potential additions to Category 3: Exercise**

**Prescription and Programming. Please rank your level of agreement with the importance of each item below.**

1. Ability to safely and appropriately progress Absolutely essential exercise to ensure appropriately intense exercise dose Very important


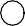

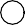

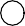

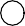

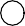


to stimulate desired adaptations while minimizing risk Of average importance is important to ensure not only safety but also Of little importance

efficacy of exercise. I'm not sure

1. Ability to adapt the program while navigating Absolutely essential changes in energy, emotional well-being, or function. Very important

Of average importance Of little importance


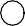

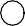

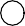

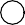

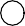


I'm not sure

1. Ability to lead patient in balance exercises. Absolutely essential Very important

Of average importance Of little importance


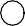

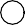

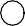

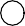

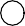


I'm not sure

1. Understanding of how to adjust treatment plan based Absolutely essential on patient finances/insurance coverage. Very important

Of average importance Of little importance


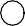

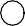

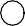

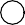

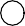


I'm not sure

1. Knowledge of when to start resistance based Absolutely essential

exercises. Very important


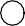

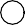

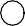

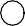

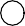


Of average importance Of little importance

I'm not sure

1. Knowledge of how to add progressive overload in an Absolutely essential exercise prescription, while also finding the balance Very important between what is enough, but what is not too much. Of average importance

Of little importance I'm not sure


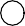

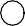

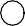

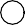

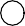


1. Understand manual skills with respect to treatment Absolutely essential status, safe and effective exercise prescription to Very important

improve outcomes and treatment adherence. Of average importance Of little importance


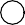

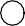

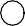

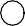

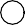


I'm not sure

1. Provide education and strategies for pacing Absolutely essential

activity throughout the day outside of physical Very important


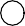

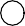

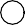

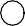

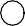


exercise activities, including avoiding sedentary Of average importance

behaviors. Of little importance

I'm not sure

1. Ability to effectively use the Borg Scale or other Absolutely essential

perceived exertion charts. Very important


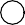

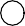

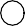

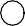

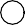


Of average importance Of little importance

I'm not sure

1. Ability to Identify and use appropriate tools to Absolutely essential


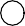

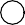

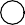


monitor progress. Very important

Of average importance Of little importance

I'm not sure

Reflecting on the results of Round 1 and the category as a whole, do you have any additional comments or suggestions for Category 3: Exercise Prescription and Programming?

If yes, please detail your comments below. If no, continue to the next page.

**Category 4: Nutrition and Weight Management n=29**

**Category agreement**

**Consensus was not reached about whether this should be a required category of knowledge for exercise oncology professionals. Nearly ¼ of respondents (24%) reported the category should not be included.**

**Notes about category**

**Concerns were raised around these competencies being “out of scope of practice” and “best left to a nutrition professional or someone with dual competency”. Two participants noted nutrition should be a “base level knowledge” but a referral should be made to a dietician for proper nutrition support/direct nutritional care. Another commented that “understanding nutrition with relation to exercise and how it impacts anatomy and physiology” is important. A suggestion was made that nutrition would be better suited as a Continuing Education Unit (CEU) or elective option. Finally, one participant noted this knowledge is part of board certification for Licensed Physical Therapists.**

**Within this section two participants noted the specific items provided a comprehensive list.**

Specific items in category

Ten specific knowledge, skills, and abilities (KSAs) were described in this category. Seven did NOT reach consensus (>90% agreement) as being a “very important” or “absolutely essential” area of knowledge for an exercise oncology professional. The three items that did reach consensus are marked with two asterisks (**) in the items listed below.

Do you think Nutrition and Weight Management should remain a required competency category for Exercise Oncology professionals?

Yes No

**Given the lack of agreement in Round 1 about the need for Nutrition and Weight Management as a required category of competency, please reconsider whether you think each should be included as an individual competency and note your response below.**

**Please note: some may be appropriate within other categories if Nutrition and Weight Management is removed.**

**Reminder: those with two asterisks (**) DID reach consensus for inclusion in Round 1.**

1. Knowledge of common effects of cancer treatment on energy balance and body composition for individuals with non-metastatic disease.**

Yes No

1. Knowledge of effects of cancer cachexia on energy balance, intake, and activity level among individuals with metastatic disease.**

Yes No

1. Knowledge of relationship between body composition as a risk factor for the development of some cancers, and possibly as a risk factor for cancer recurrence.

Yes No

1. Knowledge that many cancer survivors may use complementary and alternative medicine (CAM) approaches, and of the potential for these remedies to influence exercise testing and prescription parameters.

Yes No

1. Ability to identify unintentional weight change that may relate to disease status and recommend that the client seek appropriate medical attention.

Yes No

1. Knowledge of effect of chemotherapy and radiation on the mouth and gastrointestinal system, and the result of these changes on appetite, and food preferences and choices.

Yes No

1. Ability to discern when a participant's nutritional questions or status would be best managed by referral to a registered dietitian.**

Yes No

1. Knowledge of current nutrition guidelines during and after cancer treatment.

Yes No

1. Knowledge of hydration needs specific to cancer patients and survivors.

Yes No

1. Knowledge of safety of weight loss programs for cancer survivors.

Yes No

**The following four suggestions were made as potential additions to Category 4: Nutrition and Weight Management.**

**Please rank your level of agreement with the importance of each item below.**

**Reminder: the competency may be appropriate to include in a different category if Nutrition and Weight management is removed as a category.**

1. Understanding of the general guidelines of Absolutely essential

nutrition and how they are related to sarcopenia. Very important

Of average importance Of little importance

I'm not sure

1. Sarcopenic impact of body composition changes and Absolutely essential their impact on lean body mass and weight gain. Very important

Of average importance Of little importance

I'm not sure

1. Ability to administer an appropriate nutritional Absolutely essential

screening. Very important

Of average importance Of little importance

I'm not sure

1. Knowledge of when and how to refer to and Absolutely essential

collaborate with Registered Dieticians. Very important

Of average importance Of little importance

I'm not sure

Reflecting on the results of Round 1 and the category as a whole, do you have any additional comments or suggestions for Category 4: Nutrition and Weight Management?

If yes, please detail your comments below. If no, continue to the next page.

**Category 5: Human Behavior and Counseling n=29**

**Category agreement**

**Ninety-three percent of participants thought this should be a required category of knowledge for exercise oncology professionals.**

**Notes about category**

**Two participants raised concerns about the category as a whole. One felt the generalized knowledge in this category is met by being a board certified physical therapist. Another felt the skills in this category are important, but should not be mandatory because they can be learned in time.**

**Alternatively, one participant noted the specific items provided a comprehensive list for this category.**

Specific items in category

Eight specific knowledge, skills, and abilities (KSAs) were described in this category. The five below reached consensus (>90% agreement) as being a “very important” or “absolutely essential” area of knowledge for an exercise oncology professional.

1. Knowledge to identify a teachable moment for cancer survivors and ability to use that time to provide appropriate information and education about resuming or adopting an exercise program.
2. Knowledge of behavioral strategies that can enhance motivation and adherence (e.g. goal setting, exercise logs, planning).
3. Knowledge of the impact of cancer diagnosis and treatment on quality of life (QOL), and the potential for exercise to enhance a range of QOL outcomes for survivors (e.g. sleep, fatigue, and other factors).
4. Knowledge of how cancer and cancer treatment relate to ability and readiness to start an exercise program.
5. Demonstrate communication skills and compassion for patients/clients who have suffered the physical and psychological trauma of cancer and its management.

Do you have any concerns or comments with the statements that reached consensus for inclusion in Category 5: Human Behavior and Counseling?

If yes, please detail them in the comment box. If no, continue to next page.

**The following three KSAs did not achieve consensus.**

**Do you think these statements should be included in Category 5: Human Behavior and Counseling?**

1. General knowledge of psycho-social problems common to cancer survivors, such as depression, anxiety, fear of recurrence, sleep disturbances, body image, sexual dysfunction, and work and marital difficulties.

(Round 1 results: 86% = very important/absolute essential; 14% = of average importance) Yes No

1. Knowledge of and ability to determine effectiveness of group exercise programming vs. individual exercise to meet clients’ needs.

(Round 1 results: 66% = very important/absolute essential; 34% = of average importance) Yes No

1. Ability to facilitate the social support needs that are cancer specific including connections to websites and local support groups.

(Round 1 results: 66% = very important/absolute essential; 34% = of average importance; 4% = of little importance) Yes No

**The following eight suggestions were made as potential additions to Category 5: Human Behavior and Counseling.**

**Please rank your level of agreement with the importance of each item below.**

1. Understand the patient's goals for exercise and Absolutely essential know how to use them to set realistic expectations for Very important exercise. Of average importance

Of little importance I'm not sure

1. Knowledge of evidence-based health behavior change Absolutely essential strategies to help patients be as physically active as Very important possible. Three specific health behavior techniques Of average importance were raised: Of little importance

I'm not sure

a. Motivational Interviewing

1. Knowledge of evidence-based health behavior change Absolutely essential strategies to help patients be as physically active as Very important possible. Three specific health behavior techniques Of average importance were raised: Of little importance

I'm not sure

b. Health Coaching

1. Knowledge of evidence-based health behavior change Absolutely essential strategies to help patients be as physically active as Very important possible. Three specific health behavior techniques Of average importance were raised: Of little importance

I'm not sure

c. Cognitive Behavioral Therapy

1. Understand who is part of a patient's support Absolutely essential

system. Very important

Of average importance Of little importance

I'm not sure

1. Demonstrate an understanding of the patient's Absolutely essential personal circumstances, needs, and concerns relating Very important

to their cancer treatment. Of average importance Of little importance

I'm not sure

1. Understand common barriers to (and facilitators of) Absolutely essential exercise and be able to work with patient to overcome Very important

as many as possible. Of average importance

Of little importance I'm not sure

1. Understanding that to some patients, exercise Absolutely essential

reflects an opportunity to take some control. Very important

Of average importance Of little importance

I'm not sure

Reflecting on the results of Round 1 and the category as a whole, do you have any additional comments or suggestions for Category 5: Human Behavior and Counseling?

If yes, please detail your comments below. If no, continue to the next page.

**Category 6: Safety, Injury, Prevention, and Emergency Procedures n=29**

**Category agreement**

**Ninety-seven percent of participants thought this should be a required category of knowledge for exercise oncology professionals.**

**Notes about category**

**One participant noted the items in this category are requirements of being a Board Certified Physical Therapist.**

Specific items in category

Four specific knowledge, skills, and abilities (KSAs) were described in this category. All reached consensus (>90% agreement) as being a “very important” or “absolutely essential” area of knowledge for an exercise oncology professional.

1. Knowledge of and ability to recognize and respond to cancer-specific safety issues, such as: susceptibility to infection, musculoskeletal and orthopedic changes, unilateral edema, fatigue, lymphedema, neurological changes, osteoporosis, cognitive decline associated with treatment.
2. Knowledge of and ability to respond to cancer specific emergencies, including: sudden loss of limb function, fever in immune-incompetent patient, and mental status changes.
3. Knowledge of and ability to respond to the signs and symptoms of new onset and major life threatening complications of cancer, such as superior vena cava syndrome (SVCS), sepsis or infection, and spinal cord compression.
4. Knowledge of and ability to write-up incident documentation related to cancer specific adverse events.

Do you have any concerns or comments with the statements that reached consensus for inclusion in Category 6: Safety, Injury, Prevention, and Emergency Procedures?

If yes, please detail them in the comment box. If no, continue to next page.

Reflecting on the results of Round 1 and the category as a whole, do you have any additional comments or suggestions for Category 6: Safety, Injury, Prevention, and Emergency Procedures?

If yes, please detail your comments below. If no, continue to the next page.

**Category 7: Program Administration, Quality Assurance, & Outcome Assessment n=29**

**Category agreement**

**Ninety-three percent of participants thought this should be a required category of knowledge for exercise oncology professionals. However, consensus was reached for only 20% of individual knowledge, skills, and abilities (KSAs) described in this category.**

**Notes about category**

**One participant noted that the recommended KSAs are important to the care of any client receiving treatment, but they will be picked up in the workplace and should not be mandatory requirements of an individual. Another noted that the value of each KSA is dependent on the role of the exercise oncology professional (e.g., part of the clinical care team vs. a community partner).**

**Specific items in category**

**Ten specific knowledge, skills, and abilities (KSAs) were described in this category. Despite agreement that the category should be included, eight KSAs did NOT reach consensus (>90% agreement) as being a “very important” or “absolutely essential” area of knowledge for an exercise oncology professional. The three items that DID reach consensus are marked with two asterisks (**).**

**With this information in mind, please choose whether you think the statements below should be included in this category or not.**

1. Knowledge of role in administration and program management within a cancer center, cancer treatment facility, and outpatient setting.

Yes No

1. Knowledge of the types of exercise resources and programs available nationally and in the local community and which of these programs cater specifically to the needs of cancer survivors.

Yes No

1. Knowledge of and ability to implement effective, professional business practices and ethical promotion of personal training services to the cancer care community (e.g. physicians, nurses, social workers, physical therapists, survivors and their families).**

Yes No

1. Knowledge of the patient privacy standards and ability to implement systems to ensure confidentiality of cancer related protected health information of participants.

Yes No

1. Knowledge and ability to obtain referral from physician and communicate with physician about adverse events, abilities and limitations of survivor, and outcomes of testing and training.

Yes No

1. Ability to recommend appropriate websites and refer to other health professionals.

Yes No

1. Knowledge of reimbursement programs as eligible/available.

Yes No

1. Relevant medical/legal issues.

Yes No

1. How to establish a safe and stimulating activity environment sensitive to the physical and psychological, confidentiality needs of patients/clients with cancer including the appropriateness of group or individual therapies.**

Yes No

1. The management, evaluation and reporting of information, in verbal and written formats.

Yes No

1. Knowledge of role in administration and program management within a cancer center, cancer treatment facility, and outpatient setting.

Yes No

**The following three suggestions were made as potential additions to Category 7: Program**

**Administration, Quality Assurance, & Outcome Assessment. Please rank your level of agreement with the importance of each item below.**

1. Select appropriate objective outcome measures to Absolutely essential address needs raised patient history, including Very important

Patient Reported Outcome Measures (PROMS). Of average importance Of little importance

I'm not sure

1. Establish collaborative working professional Absolutely essential relationships with the oncology treatment and cancer Very important rehabilitation teams. Of average importance

Of little importance I'm not sure

1. Understand your role as part of a patient's Absolutely essential

multi-disciplinary care team. Very important

Of average importance Of little importance

I'm not sure

Reflecting on the results of Round 1 and the category as a whole, do you have any additional comments or suggestions for Category 7: Program Administration, Quality Assurance, and Outcome Assessment?

If yes, please detail your comments below. If no, continue to the next page.

**Category 8: Clinical and Medical Considerations n=28, n=1 missing**

**Category agreement**

**Ninety-six percent of participants thought this should be a required category of knowledge for exercise oncology professionals.**

**Notes about category**

**One participant noted the items in this category are requirements of being a Board Certified Physical Therapist.**

Specific items in category

Thirteen specific knowledge, skills, and abilities (KSAs) were described in this category. Eleven reached consensus (>90% agreement) as being a “very important” or “absolutely essential” area of knowledge for an exercise oncology professional.

1. Knowledge of the common side effects and symptoms of typical cancer treatments (surgeries, chemotherapy, radiation, hormone manipulations, other drugs).
2. Knowledge that cancer treatment may accelerate functional decline associated with aging, particularly in the elderly, and that exercise programming may need to be adjusted accordingly.
3. Knowledge of the combined effects of aging and cancer-treatment on exercise capacity and selection of appropriate testing modalities and interpretation of results.
4. Knowledge of the common sites of metastases and ability to design and implement appropriate exercise programs consistent with this knowledge.
5. Knowledge of the signs and symptoms associated with new onset lymphedema, and the major cancer types associated with increased lymphedema risk (e.g. breast, head and neck cancer).
6. Knowledge of lymphedema risk reduction practices, and exercise guidelines.
7. Knowledge of how cancer treatment may alter cardiovascular risk factors, and inappropriate far responses to exercise testing or training.
8. Knowledge of lymphatic, neurological and immune system factors in cancer survivors that may require further evaluation by medical or allied health professionals before participation in physical activity.
9. Knowledge of how common cancer treatments affects the ability of cancer survivors to perform exercise, and how to adjust programs accordingly.
10. Knowledge of the effect of cancer treatment on balance and mobility and the ability to develop an appropriate exercise program that minimizes fall/injury risk.
11. Knowledge and ability to recognize the limits in the scope of practice for exercise professionals in working with cancer survivors with complex medical issues.

Do you have any concerns or comments with the statements that reached consensus for inclusion in Category 8: Clinical and Medical Considerations?

If yes, please detail them in the comment box. If no, continue to next page.

**The following two KSAs did not achieve consensus.**

**Do you think these statements should be included in Category 8: Clinical and Medical Considerations?**

1. Knowledge of the major long-term effects of treatment among childhood cancer survivors that may require careful screening and program adaptation for these individuals.

(Round 1 results: 86% = very important/absolute essential; 11% = of average importance; 3% = not sure) Yes No

1. The structure of cancer services and the roles of different professionals involved in the care of the patient at the various stages in their management pathway.

(Round 1 results: 86% = very important/absolute essential; 14% = of average importance) Yes No

**The following eleven suggestions were made as potential additions to Category 8: Clinical and**

**Medical Considerations. Please rank your level of agreement with the importance of each item below.**

1. Ability to include specific discussions on brain Absolutely essential metastases and judgement/cognition as well as balance. Very important

Of average importance Of little importance

I'm not sure

1. Be familiar with and able to interpret medical Absolutely essential information in the context of exercise prescriptions. Very important

Of average importance Of little importance

I'm not sure

1. Understand roles and responsibilities of members of Absolutely essential a holistic multidisciplinary care team (e.g., exercise Very important

physiologist, physical therapist, occupational Of average importance

therapist, etc.). Of little importance

I'm not sure

1. Know common cancer pathophysiology, staging, Absolutely essential grading, type of cancer e.g., TNM score and how this Very important impacts exercise prescription and precautions to Of average importance

consider or implement etc. Of little importance I'm not sure

1. Understand breast cancer reconstruction. Absolutely essential Very important

Of average importance Of little importance

I'm not sure

1. General tissue healing timeframes, to then apply to Absolutely essential exercise prescription post-surgery as core Very important

foundational knowledge. Of average importance Of little importance

I'm not sure

1. Know when, where and how to seek guidance if the Absolutely essential client is not making expected gains in training. Very important

Of average importance Of little importance

I'm not sure

1. Emotional and psychological impact of reduced Absolutely essential

activity tolerance, pain, and the existential threat Very important

of a cancer diagnosis in many patients' sense of Of average importance

control and well-being. Of little importance

I'm not sure

1. Understand the symptoms specific to typical Absolutely essential

presentations of various cancer diagnoses. Very important

Of average importance Of little importance

I'm not sure

1. Ability to identify potential signs of skeletal Absolutely essential

metastases progression that may warrant further Very important

investigation. Of average importance

Of little importance I'm not sure

1. Knowledge of the expected effects of treatment and Absolutely essential their impact on patients' ability to exercise (i.e., Very important

when patients will feel well or unwell during a Of average importance

treatment cycle). Of little importance

I'm not sure

Reflecting on the results of Round 1 and the category as a whole, do you have any additional comments or suggestions for Category 8: Clinical and Medical Considerations?

If yes, please detail your comments below. If no, continue to the next page.

**Category 9: Physiology, Diagnosis, and Treatment n=28, n=1 missing**

**Category agreement**

**Ninety-three percent of participants thought this should be a required category of knowledge for exercise oncology professionals. However, consensus was reached for only ~20% of knowledge, skills, and abilities (KSAs) described in this category.**

**Notes about category**

**One participant noted the KSAs listed were outside the scope of practice for a Physical Therapist. Another noted that the category as a whole contains a lot of information that would need to be updated frequently. They suggested it should not be required knowledge for people who are not directly involved in program development, but instead this information should be part of a “knowledge seeking” requirement.**

**Specific items in category**

**The category contained eleven specific KSAs. Despite agreement that the category should be included, nine did not reach consensus (>90% agreement) as being a “very important” or “absolutely essential” area of knowledge for an exercise oncology professional. The two that DID reach consensus are marked with two asterisks (**).**

**With this information in mind, please choose whether you think the statements below should be included in this category or not.**

1. Knowledge of currently accepted screening practices for surveillance of recurrence for common cancers (e.g. mammography, colonoscopy, prostate specific antigen, pap smears).

Yes No

1. Knowledge of the pathology tests used to diagnose common cancers (e.g. biopsy, imaging technologies, and blood tests for tumor markers).

Yes No

1. Knowledge of how to communicate effectively with the major specialties with whom cancer survivors may interact, including surgery, medical oncology, radiology, dietitians, and psychologists/psychiatrists.

Yes No

1. Knowledge of the most common warning signs of recurrence for common cancers, and when to recommend that clients seek additional medical evaluation.**

Yes No

1. Understand typical durations of cancer therapy for the major cancers (breast, prostate, melanoma, ovary, lung, colon), and that therapies are continually evolving/changing.

Yes No

1. General knowledge of current cancer treatment strategies, including surgery, systemic therapies (e.g. chemotherapy) and targeted therapies (e.g, anti-angiogenesis inhibitors).**

Yes No

1. Knowledge of how lifestyle factors, including nutrition, physical activity, and heredity, influence hypothesized mechanisms of cancer etiology, reduce the risk of relapse after initial treatments, and improve long-term survival.

Yes No

1. Knowledge of relationship between body composition as a risk factor for the development of some cancers, and possibly as a risk factor for cancer recurrence.

Yes No

1. General knowledge of the descriptive epidemiology of cancer, including the prevalence, incidence, and survival statistics for the major cancer types.

Yes No

1. General knowledge of cancer biology (e.g., initiation, promotion/progression, and metastases), particularly for the four most common cancers: lung, breast, colon, and prostate.

Yes No

1. The environmental/risk factors that can cause cancer and the factors which help our bodies defend against it.

Yes No

**The following three suggestions were made as potential additions to Category 9: Physiology,**

**Diagnosis, and Treatment. Please rank your level of agreement with the importance of each item below.**

1. Understand whether the goal of treatment is Absolutely essential curative or palliative and recognize how to support a Very important

patient through each scenario. Of average importance Of little importance

I'm not sure

1. Be aware of and keep up-to-date with current Absolutely essential

research and best practice methods in the field. Very important

Of average importance Of little importance

I'm not sure

1. Recognize potential side effects of a patient's Absolutely essential

medications and potential contraindications for Very important

exercise. Of average importance

Of little importance I'm not sure

Reflecting on the results of Round 1 and the category as a whole, do you have any additional comments or suggestions for Category 9: Physiology, Diagnosis, and Treatment?

If yes, please detail your comments below. If no, continue to the next page.

**Category 10: Personal Skills and Attributes**

**A new category was identified through participant’s open-ended responses. This category reflects personal skills and attributes that were described as being important for exercise oncology professionals. Twelve items were identified in this category. While these may not be appropriate to evaluate on a certification exam, they are important for exercise oncology professionals to develop and for employers to help identify interpersonal skills necessary to succeed in the role.**

**Please rank your level of agreement about whether the following personal skills and attributes are important for an Exercise Oncology professional to be successful in their role.**

1. Ability to be flexible with programming based on a Absolutely essential patient's needs. Very important

Of average importance Of little importance

I'm not sure

1. Verbal and written communication skills necessary Absolutely essential to clearly describe programming goals, expectations, Very important

and patient progress to both patients and clinicians. Of average importance

Of little importance I'm not sure

1. Ability to empathize with patients. Absolutely essential Very important

Of average importance Of little importance

I'm not sure

1. Listening skills. Absolutely essential

Very important

Of average importance Of little importance

I'm not sure

1. Ability to build a professional network. Absolutely essential Very important

Of average importance Of little importance

I'm not sure

1. Ability to observe patient needs and respond Absolutely essential

accordingly. Very important

Of average importance Of little importance

I'm not sure

1. Ability to manage patient programming in an Absolutely essential

organized and efficient manner. Very important

Of average importance Of little importance

I'm not sure

1. Demonstrate patience in approach to a patient's Absolutely essential needs. Very important

Of average importance Of little importance

I'm not sure

1. Ability to establish rapport with patients in a Absolutely essential

therapeutic relationship. Very important

Of average importance Of little importance

I'm not sure

1. A positive approach aiming to make exercise as Absolutely essential enjoyable as possible for the patient. Very important

Of average importance Of little importance

I'm not sure

1. Problem solving/critical thinking skills. Absolutely essential Very important

Of average importance Of little importance

I'm not sure

1. Be willing to accept feedback for programming and Absolutely essential professional improvement. Very important

Of average importance Of little importance

I'm not sure

Reflecting on the results of Round 1 and the category as a whole, do you have any additional comments or suggestions for Category 10: Personal Skills and Attributes?

If yes, please detail your comments below. If no, continue to the next page.

You have reached the end of the Round 2 survey.

Please click the "Submit" button to complete the survey.

THANK YOU for contributing your time and expertise to this important project.
