# Supplementary material for: Identification of core competencies for exercise oncology professionals: A Delphi study of United States and Australian participants
Source: Cancer Med. 2024 Jul 24;13(14):e70004. doi: 10.1002/cam4.70004 (PMC11267632; doi:10.1002/cam4.70004)
Supplement: Supplementary file 1 — Data S1. [file CAM4-13-e70004-s001.zip › S3.Round3 Survey.docx]

Round 3: Exercise Oncology Workforce Survey

Thank you for sharing your time and expertise with us. Your responses will help to define the specialized skillset and training requirements an exercise professional needs to deliver exercise programming to people with cancer and move us closer to the goal of making exercise standard practice in oncology.

Exercise oncology workforce development: A Delphi study (Round 3)

We are conducting a Delphi study to reach expert consensus on the knowledge, skills, and competencies

required for exercise oncology professionals to work with people undergoing active treatment in an oncology setting. This is the final round of the survey.


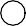
 I agree to proceed
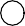
 I do not wish to participate

What is your name (first and last)?

What is your email address?

**Category 1: Exercise Physiology and Related Science n=25**

**Specific items in category**

**During Rounds 1 & 2 consensus (>90% agreement) was reached to include 8 knowledge, skills, and abilities (KSAs) in this category.**

**In this final step, please rank each KSA for:**

1. **Frequency**

**How frequently does the exercise oncology professional perform this activity?**

1. **Level of Mastery**

**Level of Mastery refers to the level of skill at which an exercise oncology professional performs during the management of patients/clients. What skill level would an exercise oncology professional demonstrate while performing this activity?**

KSA 1.1 Rarely (less than monthly)


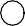

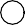

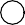

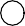

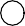


Knowledge of musculoskeletal, lymph, immunologic, Infrequently (monthly) cardiac, neurologic, endocrine, and hematologic Frequently (weekly) systems as they pertain to cancer specific exercise Very frequently (daily) issues. I'm not sure

KSA 1.1 Same as above Advanced beginner skill Competent skill level Proficient skill level Expert skill level

I'm not sure


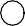

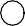

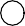

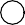

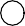


KSA 1.2 Rarely (less than monthly)

Knowledge of symptoms and psychological attributes Infrequently (monthly) that may be improved by exercise training among cancer Frequently (weekly) survivors. Very frequently (daily)


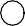

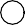

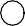

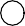

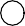


I'm not sure

KSA 1.2 Same as above Advanced beginner skill Competent skill level Proficient skill level Expert skill level

I'm not sure


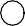

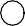

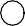

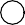

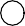


KSA 1.3 Rarely (less than monthly)

Knowledge of physiologic outcomes that may be improved Infrequently (monthly) by exercise training among cancer survivors. Frequently (weekly)


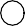

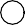

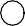

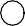

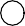


Very frequently (daily) I'm not sure

KSA 1.3 Same as above Advanced beginner skill Competent skill level Proficient skill level Expert skill level

I'm not sure


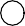

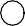

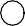

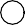

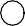


KSA 1.4 Rarely (less than monthly)


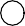

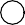

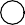

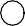

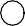


Knowledge of cancer diagnosis and treatment effects on Infrequently (monthly) physiological response to acute and chronic exercise, Frequently (weekly) particularly with regard to physical deconditioning, Very frequently (daily) body composition changes, and range of motion. I'm not sure

KSA 1.4 Same as above Advanced beginner skill Competent skill level Proficient skill level Expert skill level

I'm not sure


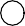

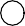

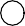

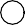

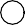


KSA 1.5 Rarely (less than monthly)

Understand the emerging evidence regarding the Infrequently (monthly)


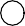

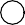

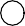

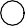

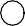


potential effects of exercise on the physiology of Frequently (weekly)

cancer treatment (e.g., accelerated aging). Very frequently (daily) I'm not sure

KSA 1.5 Same as above Advanced beginner skill Competent skill level Proficient skill level Expert skill level

I'm not sure


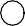

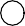

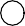

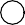

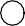


KSA 1.6 Rarely (less than monthly)

Understand the impact of exercise on oncology-related Infrequently (monthly) comorbidities, such as cardiotoxicity, diabetes, etc. Frequently (weekly)


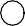

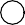

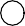

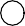

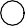


Very frequently (daily) I'm not sure

KSA 1.6 Same as above Advanced beginner skill Competent skill level Proficient skill level Expert skill level

I'm not sure


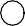

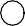

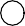

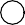

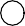


KSA 1.7 Rarely (less than monthly)

Understand how exercise can impact cognition and Infrequently (monthly) mental health. Frequently (weekly)


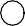

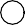

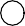

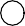

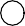


Very frequently (daily) I'm not sure

KSA 1.7 Same as above Advanced beginner skill Competent skill level Proficient skill level Expert skill level

I'm not sure


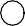

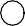

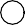

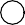

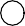


KSA 1.8 Rarely (less than monthly)

Understand how exercise can assist cancer patients Infrequently (monthly) across the disease spectrum Frequently (weekly)


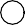

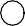

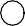

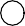

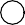


(diagnosis/treatment/recovery/palliative care). Very frequently (daily) I'm not sure

KSA 1.8 Same as above Advanced beginner skill Competent skill level Proficient skill level Expert skill level

I'm not sure


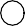

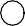

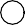

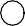

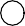


**Category 2: Health Appraisal, Fitness, and Clinical Exercise Testing**

**n=25**

**Specific items in category**

**During Rounds 1 & 2 consensus (>90% agreement) was reached to include 12 knowledge, skills, and abilities (KSAs) in this category.**

**In this final step, please rank each KSA for:**

1. **Frequency**

**How frequently does the exercise oncology professional perform this activity?**

1. **Level of Mastery**

**Level of Mastery refers to the level of skill at which an exercise oncology professional performs during the management of patients/clients. What skill level would an exercise oncology professional demonstrate while performing this activity?**

KSA 2.1 Rarely (less than monthly)


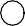

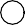

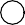

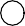

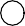


Ability to obtain a basic history regarding cancer Infrequently (monthly)

diagnosis (e.g., type, stage) and treatment (e.g., Frequently (weekly)

surgeries, systemic and targeted therapies). Very frequently (daily) I'm not sure

KSA 2.1 Same as above Advanced beginner skill Competent skill level Proficient skill level Expert skill level

I'm not sure


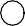

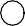

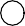

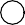

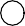


KSA 2.2 Rarely (less than monthly)

Knowledge of and the ability to recognize the adverse Infrequently (monthly) acute, chronic, and late-effects of cancer treatments. Frequently (weekly)


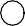

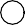

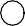

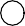

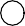


Very frequently (daily) I'm not sure

KSA 2.2 Same as above Advanced beginner skill Competent skill level Proficient skill level Expert skill level

I'm not sure


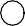

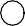

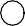


KSA 2.3 Rarely (less than monthly)

Ability to obtain medical history for other health Infrequently (monthly)

conditions (e.g. neurological, cardiovascular, Frequently (weekly) musculoskeletal, pulmonary) that may co-occur and Very frequently (daily) interact with adverse effects of cancer treatments. I'm not sure

KSA 2.3 Same as above Advanced beginner skill Competent skill level Proficient skill level Expert skill level

I'm not sure

KSA 2.4 Rarely (less than monthly)

Knowledge of and ability to discuss physiologic Infrequently (monthly) systems affected by cancer and treatment and how this Frequently (weekly) would affect the major components of fitness, Very frequently (daily)

including balance, agility, speed, flexibility, I'm not sure endurance, and strength.

KSA 2.4 Same as above Advanced beginner skill Competent skill level Proficient skill level Expert skill level

I'm not sure

KSA 2.5 Rarely (less than monthly)

Knowledge of how cancer and its treatments may alter Infrequently (monthly) balance, agility, speed, flexibility, endurance, and Frequently (weekly)

strength in cancer survivors and ability to Very frequently (daily)

select/modify and interpret tests of these fitness I'm not sure elements.

KSA 2.5 Same as above Advanced beginner skill Competent skill level Proficient skill level Expert skill level

I'm not sure

KSA 2.6 Rarely (less than monthly)

Knowledge of how cancer and its treatments may affect Infrequently (monthly) body composition in cancer survivors and ability to Frequently (weekly) select/modify and interpret tests of body composition Very frequently (daily) in cancer survivors. I'm not sure

KSA 2.6 Same as above Advanced beginner skill Competent skill level Proficient skill level Expert skill level

I'm not sure

KSA 2.7 Rarely (less than monthly)

Knowledge of categories of patients that require Infrequently (monthly)

medical clearance prior to testing or exercise Frequently (weekly)

prescription. Very frequently (daily)

I'm not sure

KSA 2.7 Same as above Advanced beginner skill Competent skill level Proficient skill level Expert skill level

I'm not sure

KSA 2.8 Rarely (less than monthly)

Knowledge of cancer-specific relative and absolute Infrequently (monthly) contraindications to exercise testing. Frequently (weekly)

Very frequently (daily) I'm not sure

KSA 2.8 Same as above Advanced beginner skill Competent skill level Proficient skill level Expert skill level

I'm not sure

KSA 2.9 Rarely (less than monthly)

How to assess, interpret and record a client's Infrequently (monthly)

baseline parameters within the categories of Frequently (weekly) cardio-respiratory endurance, muscular strength and Very frequently (daily) endurance, flexibility, range of motion, balance, body I'm not sure composition based on their physical and psychological

parameters related to their cancer but also considering other associated medical conditions such as diabetes, anxiety, depression, hypertension, arthritis, osteoporosis, cardiac disease which may be associated with cancer treatments.

KSA 2.9 Same as above Advanced beginner skill Competent skill level Proficient skill level Expert skill level

I'm not sure

KSA 2.10 Rarely (less than monthly)

Individual risk stratification using recognized Infrequently (monthly)

guidelines. Frequently (weekly)

Very frequently (daily) I'm not sure

KSA 2.10 Same as above Advanced beginner skill Competent skill level Proficient skill level Expert skill level

I'm not sure

KSA 2.11 Rarely (less than monthly)

Ability to perform subjective interview to understand Infrequently (monthly) patient's goals and patient burden of symptoms from Frequently (weekly) cancer or cancer treatment. Very frequently (daily)

I'm not sure

KSA 2.11 Same as above Advanced beginner skill Competent skill level Proficient skill level Expert skill level

I'm not sure

KSA 2.12 Rarely (less than monthly)

Ability to develop and use appropriate assessment Infrequently (monthly) protocols. Frequently (weekly)

Very frequently (daily) I'm not sure

KSA 2.12 Same as above Advanced beginner skill Competent skill level Proficient skill level Expert skill level

I'm not sure

One KSA that was suggested as an addition to this category was near consensus (> 80%).

For this KSA, please rate whether you think it should be included in the final list. Then rate the frequency of use in practice and level of mastery required.

KSA 2.13 Ability to effectively review medical chart Yes

notes to understand cancer diagnosis (e.g., No stage/grade of cancer) and treatments.

(Round 2 results: 88% = very important/absolute essential; 12% = of average importance)

KSA 2.13 Same as above Rarely (less than monthly) Infrequently (monthly) Frequently (weekly)

Very frequently (daily) I'm not sure

KSA 2.13 Same as above Advanced beginner skill Competent skill level Proficient skill level Expert skill level

I'm not sure

**Category 3: Exercise Prescription and Programming**

**n=25**

**Specific items in category**

**During Rounds 1 & 2 consensus (>90% agreement) was reached to include 23 knowledge, skills, and abilities (KSAs) in this category.**

**In this final step, please rank each KSA for:**

1. **Frequency**

**How frequently does the exercise oncology professional perform this activity?**

1. **Level of Mastery**

**Level of Mastery refers to the level of skill at which an exercise oncology professional performs during the management of patients/clients. What skill level would an exercise oncology professional demonstrate while performing this activity?**

KSA 3.1 Rarely (less than monthly)

Knowledge of current guidelines for exercise in cancer Infrequently (monthly) survivors. Frequently (weekly)

Very frequently (daily) I'm not sure

KSA 3.1 Same as above Advanced beginner skill Competent skill level Proficient skill level Expert skill level

I'm not sure

KSA 3.2 Rarely (less than monthly)

Ability to describe benefits and risks of exercise Infrequently (monthly)

training in the cancer survivor. Frequently (weekly) Very frequently (daily) I'm not sure

KSA 3.2 Same as above Advanced beginner skill Competent skill level Proficient skill level Expert skill level

I'm not sure

KSA 3.3 Rarely (less than monthly)

Ability to recognize relative and absolute Infrequently (monthly) contraindications for starting or resuming an exercise Frequently (weekly) program, and knowledge of when it is necessary to Very frequently (daily) refer participant back to an appropriate care provider I'm not sure

or when they are eligible for referral to community-based exercise programs.

KSA 3.3 Same as above Advanced beginner skill Competent skill level Proficient skill level Expert skill level

I'm not sure

KSA 3.4 Rarely (less than monthly)

Knowledge of potential for overtraining with the Infrequently (monthly)

cancer survivor. Frequently (weekly)

Very frequently (daily) I'm not sure

KSA 3.4 Same as above Advanced beginner skill Competent skill level Proficient skill level Expert skill level

I'm not sure

KSA 3.5 Rarely (less than monthly)

How to design an individualized exercise program based Infrequently (monthly) on the initial assessment. Frequently (weekly)

Very frequently (daily) I'm not sure

KSA 3.5 Same as above Advanced beginner skill Competent skill level Proficient skill level Expert skill level

I'm not sure

KSA 3.6 Rarely (less than monthly)

How to determine which baseline parameters can be Infrequently (monthly) monitored during the forthcoming exercise program in Frequently (weekly) order to assess ongoing effectiveness and if necessary Very frequently (daily) modify the program and offer alternative exercises. I'm not sure

KSA 3.6 Same as above Advanced beginner skill Competent skill level Proficient skill level Expert skill level

I'm not sure

KSA 3.7 Rarely (less than monthly)

Knowledge, skill and ability to undertake appropriate Infrequently (monthly) ongoing screening in order to detect a change in Frequently (weekly) condition and modify exercise prescription/program Very frequently (daily) based on a current medical condition. I'm not sure

KSA 3.7 Same as above Advanced beginner skill Competent skill level Proficient skill level Expert skill level

I'm not sure

KSA 3.8 Rarely (less than monthly)

Knowledge, skill and ability to undertake appropriate Infrequently (monthly) ongoing screening in order to detect a change in Frequently (weekly) condition and modify exercise prescription/program Very frequently (daily) based on time since diagnosis on or off adjuvant I'm not sure treatment.

KSA 3.8 Same as above Advanced beginner skill Competent skill level Proficient skill level Expert skill level

I'm not sure

KSA 3.9 Rarely (less than monthly)

Knowledge, skill and ability to undertake appropriate Infrequently (monthly) ongoing screening in order to detect a change in Frequently (weekly) condition and modify exercise prescription/program Very frequently (daily) based on type of current therapies (e.g. no swimming I'm not sure

during radiation).

KSA 3.9 Same as above Advanced beginner skill Competent skill level Proficient skill level Expert skill level

I'm not sure

KSA 3.10 Rarely (less than monthly)

Knowledge, skill and ability to undertake appropriate Infrequently (monthly) ongoing screening in order to detect a change in Frequently (weekly) condition and modify exercise prescription/program Very frequently (daily) based on type and recency of surgical procedures I'm not sure

(e.g., curative or reconstructive).

KSA 3.10 Same as above Advanced beginner skill Competent skill level Proficient skill level Expert skill level

I'm not sure

KSA 3.11 Rarely (less than monthly)

Knowledge, skill and ability to undertake appropriate Infrequently (monthly) ongoing screening in order to detect a change in Frequently (weekly) condition and modify exercise prescription/program Very frequently (daily) based on range of motion. I'm not sure

KSA 3.11 Same as above Advanced beginner skill Competent skill level Proficient skill level Expert skill level

I'm not sure

KSA 3.12 Rarely (less than monthly)

Knowledge, skill and ability to undertake appropriate Infrequently (monthly) ongoing screening in order to detect a change in Frequently (weekly) condition and modify exercise prescription/program Very frequently (daily) based on presence of implants. I'm not sure

KSA 3.12 Same as above Advanced beginner skill Competent skill level Proficient skill level Expert skill level

I'm not sure

KSA 3.13 Rarely (less than monthly)

Knowledge, skill and ability to undertake appropriate Infrequently (monthly) ongoing screening in order to detect a change in Frequently (weekly) condition and modify exercise prescription/program Very frequently (daily) based on amputations/fusions. I'm not sure

KSA 3.13 Same as above Advanced beginner skill Competent skill level Proficient skill level Expert skill level

I'm not sure

KSA 3.14 Rarely (less than monthly)

Knowledge, skill and ability to undertake appropriate Infrequently (monthly) ongoing screening in order to detect a change in Frequently (weekly) condition and modify exercise prescription/program Very frequently (daily) based on effects of treatment on all elements of I'm not sure

fitness (agility, speed, coordination, flexibility, strength, and endurance).

KSA 3.14 Same as above Advanced beginner skill Competent skill level Proficient skill level Expert skill level

I'm not sure

KSA 3.15 Rarely (less than monthly)

Knowledge, skill and ability to undertake appropriate Infrequently (monthly) ongoing screening in order to detect a change in Frequently (weekly) condition and modify exercise prescription/program Very frequently (daily) based on hematologic considerations (e.g. anemia, I'm not sure neutropenia).

KSA 3.15 Same as above Advanced beginner skill Competent skill level Proficient skill level Expert skill level

I'm not sure

KSA 3.16 Rarely (less than monthly)

Knowledge, skill and ability to undertake appropriate Infrequently (monthly) ongoing screening in order to detect a change in Frequently (weekly) condition and modify exercise prescription/program Very frequently (daily) based on presence of a central line (PICC or Port). I'm not sure

KSA 3.16 Same as above Advanced beginner skill Competent skill level Proficient skill level Expert skill level

I'm not sure

KSA 3.17 Rarely (less than monthly)

Knowledge, skill and ability to undertake appropriate Infrequently (monthly) ongoing screening in order to detect a change in Frequently (weekly) condition and modify exercise prescription/program Very frequently (daily) based on current adverse effects of treatment, both I'm not sure

acute and chronic.

KSA 3.17 Same as above Advanced beginner skill Competent skill level Proficient skill level Expert skill level

I'm not sure

KSA 3.18 Rarely (less than monthly)

Knowledge, skill and ability to undertake appropriate Infrequently (monthly) ongoing screening in order to detect a change in Frequently (weekly) condition and modify exercise prescription/program Very frequently (daily) based on individuals that may be at increased risk for I'm not sure

adverse late effects that could increase risks associated with exercise (e.g., heart failure).

KSA 3.18 Same as above Advanced beginner skill Competent skill level Proficient skill level Expert skill level

I'm not sure

KSA 3.19 Rarely (less than monthly)

Ability to safely and appropriately progress exercise Infrequently (monthly) to ensure appropriately intense exercise dose to Frequently (weekly) stimulate desired adaptations while minimizing risk is Very frequently (daily) important to ensure not only safety but also efficacy I'm not sure

of exercise.

KSA 3.19 Same as above Advanced beginner skill Competent skill level Proficient skill level Expert skill level

I'm not sure

KSA 3.20 Rarely (less than monthly)

Ability to adapt the program while navigating changes Infrequently (monthly) in energy, emotional well-being, or function. Frequently (weekly)

Very frequently (daily) I'm not sure

KSA 3.20 Same as above Advanced beginner skill Competent skill level Proficient skill level Expert skill level

I'm not sure

KSA 3.21 Rarely (less than monthly)

Knowledge of how to add progressive overload in an Infrequently (monthly) exercise prescription, while also finding the balance Frequently (weekly) between what is enough, but what is not too much. Very frequently (daily)

I'm not sure

KSA 3.21 Same as above Advanced beginner skill Competent skill level Proficient skill level Expert skill level

I'm not sure

KSA 3.22 Rarely (less than monthly)

Ability to effectively use the Borg Scale or other Infrequently (monthly)

perceived exertion charts. Frequently (weekly) Very frequently (daily) I'm not sure

KSA 3.22 Same as above Advanced beginner skill Competent skill level Proficient skill level Expert skill level

I'm not sure

KSA 3.23 Rarely (less than monthly)

Ability to identify and use appropriate tools to Infrequently (monthly)

monitor progress. Frequently (weekly)

Very frequently (daily) I'm not sure

KSA 3.23 Same as above Advanced beginner skill Competent skill level Proficient skill level Expert skill level

I'm not sure

Two KSAs that were suggested as additions to this category were near consensus (> 80%).

For each KSA, please rate whether you think it should be included in the final list. Then rate the frequency of use in practice and level of mastery required.

KSA 3.24 Yes

No

Knowledge of when to start resistance based exercises.

(84% = very important/absolute essential; 8% = of average importance; 4% of little importance; 4%=missing)

KSA 3.24 Same as above Rarely (less than monthly) Infrequently (monthly) Frequently (weekly)

Very frequently (daily) I'm not sure

KSA 3.24 Same as above Advanced beginner skill Competent skill level Proficient skill level Expert skill level

I'm not sure

KSA 3.25 Yes

No

Provide education and strategies for pacing activity throughout the day outside of physical exercise activities, including avoiding sedentary behaviors.

(84% = very important/absolute essential; 12% = of average importance; 4% of little importance)

KSA 3.25 Same as above Rarely (less than monthly) Infrequently (monthly) Frequently (weekly)

Very frequently (daily) I'm not sure

KSA 3.25 Same as above Advanced beginner skill Competent skill level Proficient skill level Expert skill level

I'm not sure

**Category 4: Nutrition and Weight Management**

**n=25**

**Specific items in category**

**Only 44% of respondents believed “Nutrition and Weight Management” should be an included category of KSAs for exercise oncology professionals. However, consensus (>90% agreement) was reached for 3 individual knowledge, skills, and abilities (KSAs) described in this category. These will be incorporated into a different category.**

**In this final step, please rank each KSA for:**

1. **Frequency**

**How frequently does the exercise oncology professional perform this activity?**

1. **Level of Mastery**

**Level of Mastery refers to the level of skill at which an exercise oncology professional performs during the management of patients/clients. What skill level would an exercise oncology professional demonstrate while performing this activity?**

KSA 4.1 Rarely (less than monthly)

Knowledge of common effects of cancer treatment on Infrequently (monthly) energy balance and body composition for individuals Frequently (weekly) with non-metastatic disease. Very frequently (daily)

I'm not sure

KSA 4.1 Same as above Advanced beginner skill Competent skill level Proficient skill level Expert skill level

I'm not sure

KSA 4.2 Rarely (less than monthly)

Knowledge of effects of cancer cachexia on energy Infrequently (monthly) balance, intake, and activity level among individuals Frequently (weekly) with metastatic disease. Very frequently (daily)

I'm not sure

KSA 4.2 Same as above Advanced beginner skill Competent skill level Proficient skill level Expert skill level

I'm not sure

KSA 4.3 Rarely (less than monthly)

Ability to discern when a participant's nutritional Infrequently (monthly) questions or status would be best managed by referral Frequently (weekly)

to a registered dietitian. Very frequently (daily) I'm not sure

KSA 4.3 Same as above Advanced beginner skill Competent skill level Proficient skill level Expert skill level

I'm not sure

One KSA that was suggested as an addition to this category was near consensus (> 80%).

For this KSA, please rate whether you think it should be included in the final list. Then rate the frequency of use in practice and level of mastery required.

KSA 4.4 Yes

No

Knowledge of when and how to refer to and collaborate with Registered Dieticians.

(88% = very important/absolute essential; 8% = of average importance; 4% of little importance)

KSA 4.4 Same as above Rarely (less than monthly) Infrequently (monthly) Frequently (weekly)

Very frequently (daily) I'm not sure

KSA 4.4 Same as above Advanced beginner skill Competent skill level Proficient skill level Expert skill level

I'm not sure

**Category 5: Human Behavior and Counseling**

**n=25**

**Specific items in category**

**During Rounds 1 & 2 consensus (>90% agreement) was reached to include 6 knowledge, skills, and abilities (KSAs) in this category.**

**In this final step, please rank each KSA for:**

1. **Frequency**

**How frequently does the exercise oncology professional perform this activity?**

1. **Level of Mastery**

**Level of Mastery refers to the level of skill at which an exercise oncology professional performs during the management of patients/clients. What skill level would an exercise oncology professional demonstrate while performing this activity?**

KSA 5.1 Rarely (less than monthly)

Knowledge to identify a teachable moment for cancer Infrequently (monthly) survivors and ability to use that time to provide Frequently (weekly) appropriate information and education about resuming Very frequently (daily) or adopting an exercise program. I'm not sure

KSA 5.1 Same as above Advanced beginner skill Competent skill level Proficient skill level Expert skill level

I'm not sure

KSA 5.2 Rarely (less than monthly)

Knowledge of behavioral strategies that can enhance Infrequently (monthly) motivation and adherence (e.g. goal setting, exercise Frequently (weekly) logs, planning). Very frequently (daily)

I'm not sure

KSA 5.2 Same as above Advanced beginner skill Competent skill level Proficient skill level Expert skill level

I'm not sure

KSA 5.3 Rarely (less than monthly)

Knowledge of the impact of cancer diagnosis and Infrequently (monthly) treatment on quality of life (QOL), and the potential Frequently (weekly) for exercise to enhance a range of QOL outcomes for Very frequently (daily) survivors (e.g. sleep, fatigue, and other factors). I'm not sure

KSA 5.3 Same as above Advanced beginner skill Competent skill level Proficient skill level Expert skill level

I'm not sure

KSA 5.4 Rarely (less than monthly)

Knowledge of how cancer and cancer treatment relate to Infrequently (monthly) ability and readiness to start an exercise program. Frequently (weekly)

Very frequently (daily) I'm not sure

KSA 5.4 Same as above Advanced beginner skill Competent skill level Proficient skill level Expert skill level

I'm not sure

KSA 5.5 Rarely (less than monthly)

Demonstrate communication skills and compassion for Infrequently (monthly) patients/clients who have suffered the physical and Frequently (weekly) psychological trauma of cancer and its management. Very frequently (daily)

I'm not sure

KSA 5.5 Same as above Advanced beginner skill Competent skill level Proficient skill level Expert skill level

I'm not sure

KSA 5.6 Rarely (less than monthly)

General knowledge of psycho-social problems common to Infrequently (monthly) cancer survivors, such as depression, anxiety, fear of Frequently (weekly) recurrence, sleep disturbances, body image, sexual Very frequently (daily) dysfunction, and work and marital difficulties. I'm not sure

KSA 5.6 Same as above Advanced beginner skill Competent skill level Proficient skill level Expert skill level

I'm not sure

Three KSAs that were suggested as additions to this category were near consensus (> 80%).

For each KSA, please rate whether you think it should be included in the final list. Then rate the frequency of use in practice and level of mastery required.

KSA 5.7 Yes

No

Understand the patient's goals for exercise and know how to use them to set realistic expectations for exercise.

(88% = very important/absolute essential; 8% = of average importance; 4% of little importance)

KSA 5.7 Same as above Rarely (less than monthly) Infrequently (monthly) Frequently (weekly)

Very frequently (daily) I'm not sure

KSA 5.7 Same as above Advanced beginner skill Competent skill level Proficient skill level Expert skill level

I'm not sure

KSA 5.8 Yes

No

Demonstrate an understanding of the patient's personal circumstances, needs, and concerns relating to their cancer treatment.

(84% = very important/absolute essential; 12% = of average importance; 4% of little importance)

KSA 5.8 Same as above Rarely (less than monthly) Infrequently (monthly) Frequently (weekly)

Very frequently (daily) I'm not sure

KSA 5.8 Same as above Advanced beginner skill Competent skill level Proficient skill level Expert skill level

I'm not sure

KSA 5.9 Yes

No

Understand common barriers to (and facilitators of) exercise and be able to work with a patient to overcome as many as possible.

(88% = very important/absolute essential; 8% = of average importance; 4% of little importance)

KSA 5.9 Same as above Rarely (less than monthly) Infrequently (monthly) Frequently (weekly)

Very frequently (daily) I'm not sure

KSA 5.9 Same as above Advanced beginner skill Competent skill level Proficient skill level Expert skill level

I'm not sure

**Category 6: Safety, Injury, Prevention, and Emergency Procedures**

**n=25**

**Specific items in category**

**During Rounds 1 & 2 consensus (>90% agreement) was reached to include 4 knowledge, skills, and abilities (KSAs) in this category.**

**In this final step, please rank each KSA for:**

1. **Frequency**

**How frequently does the exercise oncology professional perform this activity?**

1. **Level of Mastery**

**Level of Mastery refers to the level of skill at which an exercise oncology professional performs during the management of patients/clients. What skill level would an exercise oncology professional demonstrate while performing this activity?**

KSA 6.1 Rarely (less than monthly)

Knowledge of and ability to recognize and respond to Infrequently (monthly) cancer-specific safety issues, such as: susceptibility Frequently (weekly)

to infection, musculoskeletal and orthopedic changes, Very frequently (daily) unilateral edema, fatigue, lymphedema, neurological I'm not sure

changes, osteoporosis, cognitive decline associated with treatment.

KSA 6.1 Same as above Advanced beginner skill Competent skill level Proficient skill level Expert skill level

I'm not sure

KSA 6.2 Rarely (less than monthly)

Knowledge of and ability to respond to cancer specific Infrequently (monthly) emergencies, including: sudden loss of limb function, Frequently (weekly) fever in immune-incompetent patient, and mental status Very frequently (daily) changes. I'm not sure

KSA 6.2 Same as above Advanced beginner skill Competent skill level Proficient skill level Expert skill level

I'm not sure

KSA 6.3 Rarely (less than monthly)

Knowledge of and ability to respond to the signs and Infrequently (monthly) symptoms of new onset and major life threatening Frequently (weekly) complications of cancer, such as superior vena cava Very frequently (daily) syndrome (SVCS), sepsis or infection, and spinal cord I'm not sure compression.

KSA 6.3 Same as above Advanced beginner skill Competent skill level Proficient skill level Expert skill level

I'm not sure

KSA 6.4 Rarely (less than monthly)

Knowledge of and ability to write-up incident Infrequently (monthly)

documentation related to cancer specific events. Frequently (weekly) Very frequently (daily) I'm not sure

KSA 6.4 Same as above Advanced beginner skill Competent skill level Proficient skill level Expert skill level

I'm not sure

**Category 7: Program Administration, Quality Assurance, & Outcome Assessment**

**n=25**

**Specific items in category**

**During Rounds 1 & 2 consensus (>90% agreement) was reached to include 1 knowledge, skill, and ability (KSA) in this category.**

**In this final step, please rank each KSA for:**

1. **Frequency**

**How frequently does the exercise oncology professional perform this activity?**

1. **Level of Mastery**

**Level of Mastery refers to the level of skill at which an exercise oncology professional performs during the management of patients/clients. What skill level would an exercise oncology professional demonstrate while performing this activity?**

KSA 7.1 Rarely (less than monthly)

How to establish a safe and stimulating activity Infrequently (monthly)

environment sensitive to the physical and Frequently (weekly)

psychological, confidentiality needs of Very frequently (daily)

patients/clients with cancer including the I'm not sure appropriateness of group or individual therapies.

KSA 7.1 Same as above Advanced beginner skill Competent skill level Proficient skill level Expert skill level

I'm not sure

Three KSAs that were suggested as additions to this category were near consensus (> 80%).

For each KSA, please rate whether you think it should be included in the final list. Then rate the frequency of use in practice and level of mastery required.

KSA 7.2 Yes

No

Select appropriate objective outcome measures to address needs raised patient history, including Patient Related Outcome Measures (PROMS).

(84% = very important/absolute essential; 16% = of average importance)

KSA 7.2 Same as above Rarely (less than monthly) Infrequently (monthly) Frequently (weekly)

Very frequently (daily) I'm not sure

KSA 7.2 Same as above Advanced beginner skill Competent skill level Proficient skill level Expert skill level

I'm not sure

KSA 7.3 Yes

No

Establish collaborative working professional relationships with the oncology treatment and cancer rehabilitation teams.

(84% = very important/absolute essential; 12% = of average importance; 4% of little importance)

KSA 7.3 Same as above Rarely (less than monthly) Infrequently (monthly) Frequently (weekly)

Very frequently (daily) I'm not sure

KSA 7.3 Same as above Advanced beginner skill Competent skill level Proficient skill level Expert skill level

I'm not sure

KSA 7.4 Yes

No

Understand your role as part of a patient’s multi-disciplinary care team.

(80% = very important/absolute essential; 16% = of average importance; 4% of little importance)

KSA 7.4 Same as above Rarely (less than monthly) Infrequently (monthly) Frequently (weekly)

Very frequently (daily) I'm not sure

KSA 7.4 Same as above Advanced beginner skill Competent skill level Proficient skill level Expert skill level

I'm not sure

**Category 8: Clinical and Medical Considerations**

**n=25**

**Specific items in category**

**During Rounds 1 & 2 consensus (>90% agreement) was reached to include 18 knowledge, skills, and abilities (KSAs) in this category.**

**In this final step, please rank each KSA for:**

1. **Frequency**

**How frequently does the exercise oncology professional perform this activity?**

1. **Level of Mastery**

**Level of Mastery refers to the level of skill at which an exercise oncology professional performs during the management of patients/clients. What skill level would an exercise oncology professional demonstrate while performing this activity?**

KSA 8.1 Rarely (less than monthly)

Knowledge of the common side effects and symptoms of Infrequently (monthly) typical cancer treatments (surgeries, chemotherapy, Frequently (weekly) radiation, hormone manipulations, other drugs). Very frequently (daily)

I'm not sure

KSA 8.1 Same as above Advanced beginner skill Competent skill level Proficient skill level Expert skill level

I'm not sure

KSA 8.2 Rarely (less than monthly)

Knowledge that cancer treatment may accelerate Infrequently (monthly) functional decline associated with aging, particularly Frequently (weekly)

in the elderly, and that exercise programming may need Very frequently (daily) to be adjusted accordingly. I'm not sure

KSA 8.2 Same as above Advanced beginner skill Competent skill level Proficient skill level Expert skill level

I'm not sure

KSA 8.3 Rarely (less than monthly)

Knowledge of the combined effects of aging and Infrequently (monthly) cancer-treatment on exercise capacity and selection of Frequently (weekly) appropriate testing modalities and interpretation of Very frequently (daily) results. I'm not sure

KSA 8.3 Same as above Advanced beginner skill Competent skill level Proficient skill level Expert skill level

I'm not sure

KSA 8.4 Rarely (less than monthly)

Knowledge of the common sites of metastases and Infrequently (monthly) ability to design and implement appropriate exercise Frequently (weekly) programs consistent with this knowledge. Very frequently (daily)

I'm not sure

KSA 8.4 Same as above Advanced beginner skill Competent skill level Proficient skill level Expert skill level

I'm not sure

KSA 8.5 Rarely (less than monthly)

Knowledge of the signs and symptoms associated with Infrequently (monthly) new onset lymphedema, and the major cancer types Frequently (weekly) associated with increased lymphedema risk (e.g. Very frequently (daily)

breast, head and neck cancer). I'm not sure

KSA 8.5 Same as above Advanced beginner skill Competent skill level Proficient skill level Expert skill level

I'm not sure

KSA 8.6 Rarely (less than monthly)

Knowledge of lymphedema risk reduction practices, and Infrequently (monthly) exercise guidelines. Frequently (weekly)

Very frequently (daily) I'm not sure

KSA 8.6 Same as above Advanced beginner skill Competent skill level Proficient skill level Expert skill level

I'm not sure

KSA 8.7 Rarely (less than monthly)

Knowledge of how cancer treatment may alter Infrequently (monthly)

cardiovascular risk factors, and inappropriate far Frequently (weekly)

responses to exercise testing or training. Very frequently (daily) I'm not sure

KSA 8.7 Same as above Advanced beginner skill Competent skill level Proficient skill level Expert skill level

I'm not sure

KSA 8.8 Rarely (less than monthly)

Knowledge of lymphatic, neurological and immune system Infrequently (monthly) factors in cancer survivors that may require further Frequently (weekly) evaluation by medical or allied health professionals Very frequently (daily) before participation in physical activity. I'm not sure

KSA 8.8 Same as above Advanced beginner skill Competent skill level Proficient skill level Expert skill level

I'm not sure

KSA 8.9 Rarely (less than monthly)

Knowledge of how common cancer treatments affect the Infrequently (monthly) ability of cancer survivors to perform exercise, and Frequently (weekly) how to adjust programs accordingly. Very frequently (daily)

I'm not sure

KSA 8.9 Same as above Advanced beginner skill Competent skill level Proficient skill level Expert skill level

I'm not sure

KSA 8.10 Rarely (less than monthly)

Knowledge of the effect of cancer treatment on balance Infrequently (monthly) and mobility and the ability to develop an appropriate Frequently (weekly) exercise program that minimizes fall/injury risk. Very frequently (daily)

I'm not sure

KSA 8.10 Same as above Advanced beginner skill Competent skill level Proficient skill level Expert skill level

I'm not sure

KSA 8.11 Rarely (less than monthly)

Knowledge and ability to recognize the limits in the Infrequently (monthly) scope of practice for exercise professionals in Frequently (weekly) working with cancer survivors with complex medical Very frequently (daily) issues. I'm not sure

KSA 8.11 Same as above Advanced beginner skill Competent skill level Proficient skill level Expert skill level

I'm not sure

KSA 8.12 Rarely (less than monthly)

Knowledge of the major long-term effects of treatment Infrequently (monthly) among childhood cancer survivors that may require Frequently (weekly) careful screening and program adaptation for these Very frequently (daily) individuals. I'm not sure

KSA 8.12 Same as above Advanced beginner skill Competent skill level Proficient skill level Expert skill level

I'm not sure

KSA 8.13 Rarely (less than monthly)

Be familiar with and able to interpret medical Infrequently (monthly) information in the context of exercise prescriptions. Frequently (weekly)

Very frequently (daily) I'm not sure

KSA 8.13 Same as above Advanced beginner skill Competent skill level Proficient skill level Expert skill level

I'm not sure

KSA 8.14 Rarely (less than monthly)

Know common cancer pathophysiology, staging, grading, Infrequently (monthly) type of cancer e.g. TNM score and how this impacts Frequently (weekly) exercise prescription and precautions to consider or Very frequently (daily) implement etc. I'm not sure

KSA 8.14 Same as above Advanced beginner skill Competent skill level Proficient skill level Expert skill level

I'm not sure

KSA 8.15 Rarely (less than monthly)

General tissue healing timeframes, to then apply to Infrequently (monthly) exercise prescription post-surgery as core Frequently (weekly)

foundational knowledge. Very frequently (daily) I'm not sure

KSA 8.15 Same as above Advanced beginner skill Competent skill level Proficient skill level Expert skill level

I'm not sure

KSA 8.16 Rarely (less than monthly)

Ability to to identify potential signs of skeletal Infrequently (monthly)

metastases progression that may warrant further Frequently (weekly)

investigation. Very frequently (daily)

I'm not sure

KSA 8.16 Same as above Advanced beginner skill Competent skill level Proficient skill level Expert skill level

I'm not sure

KSA 8.17 Rarely (less than monthly)

Knowledge of the expected effects of treatment and Infrequently (monthly) their impact on patients' ability to exercise (i.e., Frequently (weekly) when patients feel well or unwell during a treatment Very frequently (daily) cycle). I'm not sure

KSA 8.17 Same as above Advanced beginner skill Competent skill level Proficient skill level Expert skill level

I'm not sure

KSA 8.18 Rarely (less than monthly)

Understand breast cancer reconstruction. Infrequently (monthly) Frequently (weekly) Very frequently (daily) I'm not sure

KSA 8.18 Same as above Advanced beginner skill Competent skill level Proficient skill level Expert skill level

I'm not sure

Two KSAs that were suggested as additions to this category were near consensus (> 80%).

For each KSA, please rate whether you think it should be included in the final list. Then rate the frequency of use in practice and level of mastery required.

KSA 8.19 Yes

No

Emotional and psychological impact of reduced activity tolerance, pain, and the existential threat of a

cancer diagnosis in many patients' sense of control and well-being.

(80% = very important/absolute essential; 12% = of average importance; 4% = of little importance; 4% = I’m not sure)

KSA 8.19 Same as above Rarely (less than monthly) Infrequently (monthly) Frequently (weekly)

Very frequently (daily) I'm not sure

KSA 8.19 Same as above Advanced beginner skill Competent skill level Proficient skill level Expert skill level

I'm not sure

KSA 8.20 Yes

No

Understand the symptoms specific to typical presentations of various cancer diagnoses.

(88% = very important/absolute essential; 4% = of average importance; 4% = of little importance; 4% = I’m not sure)

KSA 8.20 Same as above Rarely (less than monthly) Infrequently (monthly) Frequently (weekly)

Very frequently (daily) I'm not sure

KSA 8.20 Same as above Advanced beginner skill Competent skill level Proficient skill level Expert skill level

I'm not sure

**Category 9: Physiology, Diagnosis, and Treatment**

**n=25**

**Specific items in category**

**During Rounds 1 & 2 consensus (>90% agreement) was reached to include 5 knowledge, skills, and abilities (KSAs) in this category.**

**In this final step, please rank each KSA for:**

1. **Frequency**

**How frequently does the exercise oncology professional perform this activity?**

1. **Level of Mastery**

**Level of Mastery refers to the level of skill at which an exercise oncology professional performs during the management of patients/clients. What skill level would an exercise oncology professional demonstrate while performing this activity?**

KSA 9.1 Rarely (less than monthly)

Knowledge of the most common warning signs of Infrequently (monthly) recurrence for common cancers, and when to recommend Frequently (weekly) that clients seek additional medical evaluation. Very frequently (daily)

I'm not sure

KSA 9.1 Same as above Advanced beginner skill Competent skill level Proficient skill level Expert skill level

I'm not sure

KSA 9.2 Rarely (less than monthly)

General knowledge of current cancer treatment Infrequently (monthly)

strategies, including surgery, systemic therapies Frequently (weekly)

(e.g. chemotherapy) and targeted therapies (e.g, Very frequently (daily)

anti-angiogenesis inhibitors). I'm not sure

KSA 9.2 Same as above Advanced beginner skill Competent skill level Proficient skill level Expert skill level

I'm not sure

KSA 9.3 Rarely (less than monthly)

Knowledge of how lifestyle factors, including Infrequently (monthly)

nutrition, physical activity, and heredity, influence Frequently (weekly) hypothesized mechanisms of cancer etiology, reduce the Very frequently (daily) risk of relapse after initial treatments, and improve I'm not sure

long-term survival.

KSA 9.3 Same as above Advanced beginner skill Competent skill level Proficient skill level Expert skill level

I'm not sure

KSA 9.4 Rarely (less than monthly)

Be aware of and keep up-to-date with current research Infrequently (monthly) and best practice methods in the field. Frequently (weekly)

Very frequently (daily) I'm not sure

KSA 9.4 Same as above Advanced beginner skill Competent skill level Proficient skill level Expert skill level

I'm not sure

KSA 9.5 Rarely (less than monthly)

Recognize potential side effects of a patient's Infrequently (monthly)

medications and potential contraindications for Frequently (weekly)

exercise. Very frequently (daily)

I'm not sure

KSA 9.5 Same as above Advanced beginner skill Competent skill level Proficient skill level Expert skill level

I'm not sure

One KSA that was suggested as an addition to this category was near consensus (> 80%).

For this KSA, please rate whether you think it should be included in the final list. Then rate the frequency of use in practice and level of mastery required.

KSA 9.6 Yes

No

Understand whether the goal of treatment is curative or palliative and recognize how to support a patient through each scenario.

(88% = very important/absolute essential; 4% = of average importance; 4% = of little importance; 4% I'm not sure)

KSA 9.6 Same as above Rarely (less than monthly) Infrequently (monthly) Frequently (weekly)

Very frequently (daily) I'm not sure

KSA 9.6 Same as above Advanced beginner skill Competent skill level Proficient skill level Expert skill level

I'm not sure

**Category 10: Personal Skills and Attributes**

**n=25**

**Specific items in category**

**During Rounds 1 & 2 consensus (>90% agreement) was reached to include 9 knowledge, skills, and abilities (KSAs) in this category.**

**In this final step, please rank each KSA for:**

1. **Frequency**

**How frequently does the exercise oncology professional perform this activity?**

1. **Level of Mastery**

**Level of Mastery refers to the level of skill at which an exercise oncology professional performs during the management of patients/clients. What skill level would an exercise oncology professional demonstrate while performing this activity?**

KSA 10.1 Rarely (less than monthly)

Ability to be flexible with programming based on a Infrequently (monthly) patient's needs. Frequently (weekly)

Very frequently (daily) I'm not sure

KSA 10.1 Same as above Advanced beginner skill Competent skill level Proficient skill level Expert skill level

I'm not sure

KSA 10.2 Rarely (less than monthly)

Verbal and written communication skills necessary to Infrequently (monthly) clearly describe programming goals, expectations, and Frequently (weekly) patient progress to both patients and clinicians. Very frequently (daily)

I'm not sure

KSA 10.2 Same as above Advanced beginner skill Competent skill level Proficient skill level Expert skill level

I'm not sure

KSA 10.3 Rarely (less than monthly)

Ability to empathize with patients. Infrequently (monthly) Frequently (weekly) Very frequently (daily) I'm not sure

KSA 10.3 Same as above Advanced beginner skill Competent skill level Proficient skill level Expert skill level

I'm not sure

KSA 10.4 Rarely (less than monthly)

Listening skills. Infrequently (monthly)

Frequently (weekly) Very frequently (daily) I'm not sure

KSA 10.4 Same as above Advanced beginner skill Competent skill level Proficient skill level Expert skill level

I'm not sure

KSA 10.5 Rarely (less than monthly)

Ability to observe patient needs and respond Infrequently (monthly)

accordingly. Frequently (weekly)

Very frequently (daily) I'm not sure

KSA 10.5 Same as above Advanced beginner skill Competent skill level Proficient skill level Expert skill level

I'm not sure

KSA 10.6 Rarely (less than monthly)

Demonstrate patience in approach to a patient's needs. Infrequently (monthly)

Frequently (weekly) Very frequently (daily) I'm not sure

KSA 10.6 Same as above Advanced beginner skill Competent skill level Proficient skill level Expert skill level

I'm not sure

KSA 10.7 Rarely (less than monthly)

Ability to establish rapport with patients in a Infrequently (monthly)

therapeutic relationship. Frequently (weekly) Very frequently (daily) I'm not sure

KSA 10.7 Same as above Advanced beginner skill Competent skill level Proficient skill level Expert skill level

I'm not sure

KSA 10.8 Rarely (less than monthly)

Problem solving/critical thinking skills. Infrequently (monthly) Frequently (weekly) Very frequently (daily) I'm not sure

KSA 10.8 Same as above Advanced beginner skill Competent skill level Proficient skill level Expert skill level

I'm not sure

KSA 10.9 Rarely (less than monthly)

Be willing to accept feedback for programming and Infrequently (monthly) professional improvement. Frequently (weekly)

Very frequently (daily) I'm not sure

KSA 10.9 Same as above Advanced beginner skill Competent skill level Proficient skill level Expert skill level

I'm not sure

Two KSAs that were suggested as additions to this category were near consensus (> 80%).

For each KSA, please rate whether you think it should be included in the final list. Then rate the frequency of use in practice and level of mastery required.

KSA 10.10 Yes

No

A positive approach to the work aiming to make exercise as enjoyable as possible for the patient.

(88% = very important/absolute essential; 12% = of average importance)

KSA 10.10 Same as above Rarely (less than monthly) Infrequently (monthly) Frequently (weekly)

Very frequently (daily) I'm not sure

KSA 10.10 Same as above Advanced beginner skill Competent skill level Proficient skill level Expert skill level

I'm not sure

KSA 10.11 Yes

No

Ability to manage patient programming in an organized and efficient manner.

(88% = very important/absolute essential; 12% = of average importance)

KSA 10.11 Same as above Rarely (less than monthly) Infrequently (monthly) Frequently (weekly)

Very frequently (daily) I'm not sure

KSA 10.11 Same as above Advanced beginner skill Competent skill level Proficient skill level Expert skill level

I'm not sure

Reflecting on the results of Round 3 , do you have any final comments or suggestions about the specialized sillset and training requirements an exercise physiologist needs to deliver exercise programming to people with cancer?

If yes, please detail your comments below.

You have reached the end of the Round 3 survey. This is the FINAL survey in this study.

THANK YOU for contributing your time and expertise to this important project. After you submit the survey, you will receive an email with a link to a gift card as a token of our appreciation.

Please click the "Submit" button to complete the survey.
