# Supplementary material for: Identification of core competencies for exercise oncology professionals: A Delphi study of United States and Australian participants
Source: Cancer Med. 2024 Jul 24;13(14):e70004. doi: 10.1002/cam4.70004 (PMC11267632; doi:10.1002/cam4.70004)
Supplement: Supplementary file 1 — Data S1. [file CAM4-13-e70004-s001.zip › S4.Complete consensus process.docx]

S4. Comprehensive results of the competencies assessed across the three-round Delphi study

Legend:

The different color boxes denote how every item was managed in each round:

- Green=achieved >90% consensus
- Yellow=did not achieve >90% and was re-presented to the group
- Red=did not achieve >90% and was removed from further analysis
- Black=was not included in the round

The bold competencies were included in the final list; the non-bold competencies were eliminated during the Delphi process

| Category 1: Exercise physiology and related exercise science (100%) | | | | | |
| --- | --- | --- | --- | --- | --- |
| **Specific Knowledge, Skill, Ability (KSA) for evaluation** | **Source** | **Round 1 (n=29)** | **Round 2 (n=25)** | **Round 3 (n=24)** | |
| 1. **Knowledge of physiologic outcomes that may be improved by exercise training among cancer survivors.** | ACSM |  |  | Frequency  Rarely = 0%  Infreq = 0%  Freq = 12%  Very Freq = 88%  Not sure = 0% | Mastery  Adv beg = 4%  Comp = 21%  Proficient = 42%  Expert = 33%  Not sure = 0% |
| 1. **Knowledge of symptoms and psychological attributes that may be improved by exercise training among cancer survivors.** | ACSM |  |  | Frequency  Rarely = 0%  Infreq = 4%  Freq = 25%  Very Freq = 71%  Not sure = 0% | Mastery  Adv beg = 4%  Comp = 17%  Proficient = 50%  Expert = 29%  Not sure = 0% |
| 1. **Knowledge of lymph, immunologic, cardiac, neurologic, endocrine and hematologic systems as they pertain to cancer specific exercise issues.** | ACSM |  |  | Frequency  Rarely = 0%  Infreq = 0%  Freq = 25%  Very Freq = 75%  Not sure = 0% | Mastery  Adv beg = 4%  Comp = 21%  Proficient = 37%  Expert = 37%  Not sure = 0% |
| 1. Knowledge of acute and chronic effects of exercise on temperature regulation and the adverse thermoregulatory/vasomotor symptoms (e.g., hot flashes) experienced by many cancer survivors. | ACSM | 72% = very important/ absolutely essential  28% = of average importance |  |  | |
| 1. **Knowledge of cancer diagnosis and treatment effects on physiological response to acute and chronic exercise, particularly with regard to physical deconditioning, body composition changes, and range of motion.** | ACSM + CanRehab |  |  | Frequency  Rarely = 0%  Infreq = 0%  Freq = 17%  Very Freq = 83%  Not sure = 0% | Mastery  Adv beg = 4%  Comp = 8%  Proficient = 38%  Expert = 50%  Not sure = 0% |
| 1. **Add musculoskeletal to the list in the current statement: Knowledge of lymph, immunologic, cardiac, neurologic, endocrine and hematologic systems as they pertain to cancer specific exercise issues.*** | Participant addition | N/A |  |  | |
| 1. **Understand the emerging evidence regarding the potential effects of exercise on the physiology of cancer treatment (e.g., accelerated aging).** | Participant addition | N/A |  | Frequency  Rarely = 0%  Infreq = 13%  Freq = 54%  Very Freq = 33%  Not sure = 0% | Mastery  Adv beg = 8%  Comp = 21%  Proficient = 50%  Expert = 17%  Not sure = 0% |
| 1. **Understand the impact of exercise on oncology related comorbidities such as cardiotoxicity, diabetes, etc.** | Participant addition | N/A |  | Frequency  Rarely = 0%  Infreq = 0%  Freq = 42%  Very Freq = 58%  Not sure = 0% | Mastery  Adv beg = 4%  Comp = 13%  Proficient = 50%  Expert = 33%  Not sure = 0% |
| 1. **Understand how exercise can impact cognition and mental health.** | Participant addition | N/A |  | Frequency  Rarely = 0%  Infreq = 13%  Freq = 50%  Very Freq = 37%  Not sure = 0% | Mastery  Adv beg = 8%  Comp = 25%  Proficient = 50%  Expert = 17%  Not sure = 0% |
| 1. **Understand how exercise can assist cancer patients across the disease spectrum (diagnosis, treatment, recovery, palliative care).** | Participant addition | N/A |  | Frequency  Rarely = 0%  Infreq = 4%  Freq = 25%  Very Freq = 71%  Not sure = 0% | Mastery  Adv beg = 4%  Comp = 12%  Proficient = 38%  Expert = 46%  Not sure = 0% |

*included as part of KSA 1.3, not as an additional item.

| Category 2: Health appraisal, fitness, and clinical exercise testing (93%) | | | | | |
| --- | --- | --- | --- | --- | --- |
| **Specific Knowledge, Skill, Ability (KSA) for evaluation** | **Source** | **Round 1 (n=29)** | **Round 2 (n=25)** | **Round 3 (n=24)** | |
| 1. **Ability to obtain a basic history regarding cancer diagnosis (e.g., type, stage) and treatment (e.g., surgeries, systemic and targeted therapies).** | ACSM |  |  | Frequency  Rarely = 0%  Infreq = 4%  Freq = 17%  Very Freq = 79%  Not sure = 0% | Mastery  Adv beg = 12%  Comp = 17%  Proficient = 25%  Expert = 46%  Not sure = 0% |
| 1. **Knowledge of and the ability to recognize the adverse acute, chronic, and late-effects of cancer treatments.** | ACSM |  |  | Frequency  Rarely = 0%  Infreq = 0%  Freq = 37%  Very Freq = 63%  Not sure = 0% | Mastery  Adv beg = 0%  Comp = 17%  Proficient = 33%  Expert = 50%  Not sure = 0% |
| 1. **Ability to obtain medical history for other health conditions (e.g. neurological, cardiovascular, musculoskeletal, pulmonary) that may co-occur and interact with adverse effects of cancer treatments.** | ACSM |  |  | Frequency  Rarely = 0%  Infreq = 4%  Freq = 17%  Very Freq = 79%  Not sure = 0% | Mastery  Adv beg = 12%  Comp = 17%  Proficient = 25%  Expert = 46%  Not sure = 0% |
| 1. **Knowledge of and ability to discuss physiologic systems affected by cancer and treatment and how this would affect the major components of fitness, including balance, agility, speed, flexibility, endurance, and strength.** | ACSM |  |  | Frequency  Rarely = 0%  Infreq = 0%  Freq = 37%  Very Freq = 63%  Not sure = 0% | Mastery  Adv beg = 0%  Comp = 13%  Proficient = 54%  Expert = 33%  Not sure = 0% |
| 1. **Knowledge of how cancer and its treatments may alter balance, agility, speed, flexibility, endurance, and strength in cancer survivors and ability to select/modify and interpret tests of these fitness elements.** | ACSM |  |  | Frequency  Rarely = 0%  Infreq = 0%  Freq = 25%  Very Freq = 67%  Not sure = 4%  Missing = 4% | Mastery  Adv beg = 0%  Comp = 12%  Proficient = 42%  Expert = 42%  Not sure = 4% |
| 1. **Knowledge of how cancer and its treatments may affect body composition in cancer survivors and ability to select/modify and interpret tests of body composition in cancer survivors.** | ACSM |  |  | Frequency  Rarely = 4%  Infreq = 13%  Freq = 33%  Very Freq = 50%  Not sure = 0% | Mastery  Adv beg = 4%  Comp = 17%  Proficient = 54%  Expert = 25%  Not sure = 0% |
| 1. **Knowledge of categories of patients that require medical clearance prior to testing or exercise prescription.** | ACSM |  |  | Frequency  Rarely = 4%  Infreq = 4%  Freq = 38%  Very Freq = 54%  Not sure = 0% | Mastery  Adv beg = 4%  Comp = 17%  Proficient = 42%  Expert = 37%  Not sure = 0% |
| 1. **Knowledge of cancer-specific relative and absolute contraindications to exercise testing.** | ACSM |  |  | Frequency  Rarely = 4%  Infreq = 12%  Freq = 42%  Very Freq = 42%  Not sure = 0% | Mastery  Adv beg = 4%  Comp = 8%  Proficient = 46%  Expert = 42%  Not sure = 0% |
| 1. **How to assess, interpret and record a client's baseline parameters within the categories of cardio-respiratory endurance, muscular strength and endurance, flexibility, range of motion, balance, body composition based on their physical and psychological parameters related to their cancer but also considering other associated medical conditions such as diabetes, anxiety, depression, hypertension, arthritis, osteoporosis, cardiac disease which may be associated with cancer treatments.** | CanRehab |  |  | Frequency  Rarely = 0%  Infreq = 0%  Freq = 37%  Very Freq = 63%  Not sure = 0% | Mastery  Adv beg = 0%  Comp = 17%  Proficient = 46%  Expert = 37%  Not sure = 0% |
| 1. **Individual risk stratification using recognized guidelines.** | CanRehab | 83% = very important or absolutely essential  17% = of average importance |  | Frequency  Rarely = 0%  Infreq = 13%  Freq = 54%  Very Freq = 33%  Not sure = 0% | Mastery  Adv beg = 4%  Comp = 13%  Proficient = 58%  Expert = 25%  Not sure = 0% |
| 1. **Ability to perform subjective interview to understand patient’s goals and patient burden of symptoms from cancer or cancer treatment.** | Participant addition | N/A |  | Frequency  Rarely = 0%  Infreq = 4%  Freq = 21%  Very Freq = 75%  Not sure = 0% | Mastery  Adv beg = 4%  Comp = 17%  Proficient = 54%  Expert = 25%  Not sure = 0% |
| 1. **Ability to develop and use appropriate assessment protocols.** | Participant addition | N/A |  | Frequency  Rarely = 0%  Infreq = 13%  Freq = 29%  Very Freq = 58%  Not sure = 0% | Mastery  Adv beg = 4%  Comp = 17%  Proficient = 42%  Expert = 37%  Not sure = 0% |
| 1. **Ability to effectively review medical chart notes to understand cancer diagnosis (e.g., stage/grade of cancer) and treatments.** | Participant addition | N/A | 88% = very important or absolutely essential  12% = of average importance | Frequency  Rarely = 4%  Infreq = 0%  Freq = 42%  Very Freq = 54%  Not sure = 0 | Mastery  Adv beg = 0%  Comp = 29%  Proficient = 46%  Expert = 25%  Not sure = 0% |

| Category 3: Exercise prescription and programming (97%) |  |  |  |  | |
| --- | --- | --- | --- | --- | --- |
| **Specific Knowledge, Skill, Ability (KSA) for evaluation** | **Source** | **Round 1 (n=29)** | **Round 2 (n=25)** | **Round 3 (n=24)** | |
| 1. **Knowledge of current guidelines for exercise in cancer survivors.** | ACSM + CanRehab |  |  | Frequency  Rarely = 4%  Infreq = 4%  Freq = 33%  Very Freq = 58%  Not sure = 0% | Mastery  Adv beg = 8%  Comp = 17%  Proficient = 38%  Expert = 38%  Not sure = 0% |
| 1. **Ability to describe benefits and risks of exercise training in the cancer survivor.** | ACSM + CanRehab |  |  | Frequency  Rarely = 0%  Infreq = 0%  Freq = 21%  Very Freq = 79%  Not sure = 0% | Mastery  Adv beg = 8%  Comp = 17%  Proficient = 29%  Expert = 46%  Not sure = 0% |
| 1. **Ability to recognize relative and absolute contraindications for starting or resuming an exercise program, and knowledge of when it is necessary to refer participant back to an appropriate care provider or when they are eligible for referral to community-based exercise programs.** | ACSM + CanRehab |  |  | Frequency  Rarely = 8%  Infreq = 4%  Freq = 38%  Very Freq = 50%  Not sure = 0% | Mastery  Adv beg = 0%  Comp = 8%  Proficient = 42%  Expert = 50%  Not sure = 0% |
| 1. **Knowledge of potential for overtraining with the cancer survivor.** | ACSM |  |  | Frequency  Rarely = 4%  Infreq = 21%  Freq = 50%  Very Freq = 25%  Not sure = 0% | Mastery  Adv beg = 0%  Comp = 25%  Proficient = 54%  Expert = 21%  Not sure = 0% |
| 1. **How to design an individualized exercise program based on the initial assessment.** | CanRehab |  |  | Frequency  Rarely = 0%  Infreq = 0%  Freq = 29%  Very Freq = 71%  Not sure = 0% | Mastery  Adv beg = 0%  Comp = 8%  Proficient = 42%  Expert = 50%  Not sure = 0% |
| 1. **How to determine which baseline parameters can be monitored during the forthcoming exercise program in order to assess ongoing effectiveness and if necessary modify the program and offer alternative exercises.** | CanRehab |  |  | Frequency  Rarely = 0%  Infreq = 12%  Freq = 42%  Very Freq = 46%  Not sure = 0% | Mastery  Adv beg = 0%  Comp = 8%  Proficient = 58%  Expert = 29%  Not sure = 0%  Missing = 4% |
| 1. How to ensure carers/caregivers are comfortable with the principles of the exercise prescription. | CanRehab | 66% = very important or absolutely essential  34% = of average importance |  |  | |
| 1. The important general lifestyle factors after cancer and the ability to signpost clients to suitable written materials regarding weight control, adequate protein intake relevant to the level of exercise, healthy and unhealthy diets, supplements, smoking, sun exposure, carcinogens and environmental pollutants. | CanRehab | 69% = very important or absolutely essential  31% = of average importance |  |  | |
| 1. Knowledge of and ability to use appropriate sun protection for outdoor programming. | ACSM | 48% = very important or absolutely essential  48% = of average importance  4% = of little importance |  |  | |
| 1. **Knowledge, skill and ability to undertake appropriate ongoing screening in order to detect a change in condition and modify exercise prescription/program based on a current medical condition.** | ACSM + CanRehab |  |  | Frequency  Rarely = 0%  Infreq = 12%  Freq = 38%  Very Freq = 50%  Not sure = 0% | Mastery  Adv beg = 0%  Comp = 17%  Proficient = 50%  Expert = 33%  Not sure = 0% |
| 1. **Knowledge, skill and ability to undertake appropriate ongoing screening in order to detect a change in condition and modify exercise prescription/program based on time since diagnosis on or off adjuvant treatment.** | ACSM + CanRehab |  |  | Frequency  Rarely = 0%  Infreq = 12%  Freq = 50%  Very Freq = 38%  Not sure = 0% | Mastery  Adv beg = 0%  Comp = 12%  Proficient = 46%  Expert = 42%  Not sure = 0% |
| 1. **Knowledge, skill and ability to undertake appropriate ongoing screening in order to detect a change in condition and modify exercise prescription/program based on type of current therapies (e.g. no swimming during radiation).** | ACSM + CanRehab |  |  | Frequency  Rarely = 0%  Infreq = 4%  Freq = 58%  Very Freq = 38%  Not sure = 0% | Mastery  Adv beg = 0%  Comp = 8%  Proficient = 54%  Expert = 38%  Not sure = 0% |
| 1. **Knowledge, skill and ability to undertake appropriate ongoing screening in order to detect a change in condition and modify exercise prescription/program based on type and recency of surgical procedures (e.g., curative or reconstructive).** | ACSM + CanRehab |  |  | Frequency  Rarely = 0%  Infreq = 12%  Freq = 58%  Very Freq = 29%  Not sure = 0% | Mastery  Adv beg = 0%  Comp = 4%  Proficient = 58%  Expert = 29%  Not sure = 0% |
| 1. **Knowledge, skill and ability to undertake appropriate ongoing screening in order to detect a change in condition and modify exercise prescription/program based on range of motion.** | ACSM + CanRehab |  |  | Frequency  Rarely = 0%  Infreq = 17%  Freq = 50%  Very Freq = 29%  Not sure = 4% | Mastery  Adv beg = 8%  Comp = 17%  Proficient = 38%  Expert = 33%  Not sure = 4% |
| 1. **Knowledge, skill and ability to undertake appropriate ongoing screening in order to detect a change in condition and modify exercise prescription/program based on presence of implants** | ACSM + CanRehab |  |  | Frequency  Rarely = 12%  Infreq = 25%  Freq = 46%  Very Freq = 17%  Not sure = 0% | Mastery  Adv beg = 4%  Comp = 25%  Proficient = 46%  Expert = 25%  Not sure = 0% |
| 1. **Knowledge, skill and ability to undertake appropriate ongoing screening in order to detect a change in condition and modify exercise prescription/program based on amputations/fusions.** | ACSM + CanRehab |  |  | Frequency  Rarely = 25%  Infreq = 46%  Freq = 21%  Very Freq = 8%  Not sure = 0% | Mastery  Adv beg = 4%  Comp = 21%  Proficient = 46%  Expert = 29%  Not sure = 0% |
| 1. **Knowledge, skill and ability to undertake appropriate ongoing screening in order to detect a change in condition and modify exercise prescription/program based on effects of treatment on all elements of fitness (agility, speed, coordination, flexibility, strength, and endurance).** | ACSM + CanRehab |  |  | Frequency  Rarely = 0%  Infreq = 4%  Freq = 42%  Very Freq = 46%  Not sure = 8% | Mastery  Adv beg = 4%  Comp = 17%  Proficient = 42%  Expert = 29%  Not sure = 8% |
| 1. **Knowledge, skill and ability to undertake appropriate ongoing screening in order to detect a change in condition and modify exercise prescription/program based on hematologic considerations (e.g. anemia, neutropenia).** | ACSM + CanRehab |  |  | Frequency  Rarely = 4%  Infreq = 17%  Freq = 50%  Very Freq = 29%  Not sure = 0% | Mastery  Adv beg = 0%  Comp = 8%  Proficient = 58%  Expert = 38%  Not sure = 0% |
| 1. **Knowledge, skill and ability to undertake appropriate ongoing screening in order to detect a change in condition and modify exercise prescription/program based on presence of a central line (PICC or Port).** | ACSM + CanRehab |  |  | Frequency  Rarely = 0%  Infreq = 25%  Freq = 67%  Very Freq = 8%  Not sure = 0% | Mastery  Adv beg = 0%  Comp = 12%  Proficient = 63%  Expert = 25%  Not sure = 0% |
| 1. **Knowledge, skill and ability to undertake appropriate ongoing screening in order to detect a change in condition and modify exercise prescription/program based on current adverse effects of treatment, both acute and chronic.** | ACSM + CanRehab |  |  | Frequency  Rarely = 0%  Infreq = 0%  Freq = 38%  Very Freq = 62%  Not sure = 0% | Mastery  Adv beg = 0%  Comp = 8%  Proficient = 46%  Expert = 46%  Not sure = 0% |
| 1. **Knowledge, skill and ability to undertake appropriate ongoing screening in order to detect a change in condition and modify exercise prescription/program based on individuals that may be at increased risk for adverse late effects that could increase risks associated with exercise (e.g., heart failure).** | ACSM + CanRehab |  |  | Frequency  Rarely = 4%  Infreq = 12%  Freq = 63%  Very Freq = 21%  Not sure = 0% | Mastery  Adv beg = 0%  Comp = 12%  Proficient = 54%  Expert = 33%  Not sure = 0% |
| 1. **Ability to safely and appropriately progress exercise to ensure appropriately intense exercise dose to stimulate desired adaptations while minimizing risk is important to ensure not only safety but also efficacy of exercise.** | Participant addition | N/A |  | Frequency  Rarely = 0%  Infreq = 0%  Freq = 42%  Very Freq = 54%  Not sure = 4% | Mastery  Adv beg = 4%  Comp = 8%  Proficient = 42%  Expert = 42%  Not sure = 4% |
| 1. **Ability to adapt the program on demand in response to highs and lows of energy, emotion or function.** | Participant addition | N/A |  | Frequency  Rarely = 0%  Infreq = 0%  Freq = 46%  Very Freq = 54%  Not sure = 0% | Mastery  Adv beg = 4%  Comp = 12%  Proficient = 50%  Expert = 33%  Not sure = 0% |
| 1. Ability to lead patient in balance exercises. | Participant addition | N/A |  |  | |
| 1. Understanding of how to adjust treatment plan based on patient finances/insurance coverage. | Participant addition | N/A |  |  | |
| 1. **When to start resistance based exercises.** | Participant addition | N/A | 84% = very important or absolutely essential  8% = of average importance  4% = of little importance | Frequency  Rarely = 0%  Infreq = 0%  Freq = 42%  Very Freq = 58%  Not sure = 0% | Mastery  Adv beg = 8%  Comp = 17%  Proficient = 46%  Expert = 29%  Not sure = 0% |
| 1. **Knowledge of how to add progressive overload in an exercise prescription while also finding the balance between what is enough, but what is not too much.** | Participant addition | N/A |  | Frequency  Rarely = 0%  Infreq = 4%  Freq = 21%  Very Freq = 75%  Not sure = 0% | Mastery  Adv beg = 4%  Comp = 12%  Proficient = 33%  Expert = 50%  Not sure = 0% |
| 1. Understand manual skills with respect to treatment status, safe and effective exercise prescription to improve outcomes and treatment adherence. | Participant addition | N/A |  |  | |
| 1. **Provide education and strategies for pacing activity throughout the day outside of physical exercise activities, including avoiding sedentary behaviors.** | Participant addition | N/A | 84% = very important or absolutely essential  12% = of average importance  4% = of little importance | Frequency  Rarely = 0%  Infreq = 0%  Freq = 67%  Very Freq = 33%  Not sure = 0% | Mastery  Adv beg = 12%  Comp = 12%  Proficient = 58%  Expert = 17%  Not sure = 0% |
| 1. **Ability to effectively use the Borg Scale or other perceived exertion charts.** | Participant addition | N/A |  | Frequency  Rarely = 0%  Infreq = 4%  Freq = 29%  Very Freq = 67%  Not sure = 0% | Mastery  Adv beg = 12%  Comp = 29%  Proficient = 17%  Expert = 38%  Not sure = 0%  Missing = 4% |
| 1. **Identify and use appropriate tools to monitor progress.** | Participant addition | N/A |  | Frequency  Rarely = 0%  Infreq = 8%  Freq = 50%  Very Freq = 42%  Not sure = 0% | Mastery  Adv beg = 17%  Comp = 17%  Proficient = 29%  Expert = 38%  Not sure = 0% |

| Category 4: Nutrition and weight management (76%) | | | | | |
| --- | --- | --- | --- | --- | --- |
| **Specific Knowledge, Skill, Ability (KSA) for evaluation** | **Source** | **Round 1 (n=29)** | **Round 2 (n=25)** | **Round 3 (n=24)** | |
| 1. **Knowledge of common effects of cancer treatment on energy balance and body composition for individuals with non-metastatic disease.** | ACSM |  |  | Frequency  Rarely = 0%  Infreq = 8%  Freq = 67%  Very Freq = 25%  Not sure = 0% | Mastery  Adv beg = 4%  Comp = 17%  Proficient = 50%  Expert = 29%  Not sure = 0% |
| 1. **Knowledge of effects of cancer cachexia on energy balance, intake, and activity level among individuals with metastatic disease.** | ACSM |  |  | Frequency  Rarely = 0%  Infreq = 29%  Freq = 46%  Very Freq = 25%  Not sure = 0% | Mastery  Adv beg = 0%  Comp = 4%  Proficient = 54%  Expert = 42%  Not sure = 0% |
| 1. Knowledge of relationship between body composition as a risk factor for the development of some cancers, and possibly as a risk factor for cancer recurrence. | ACSM + CanRehab |  |  |  | |
| 1. Knowledge that many cancer survivors may use complementary and alternative medicine (CAM) approaches, and of the potential for these remedies to influence exercise testing and prescription parameters. | ACSM |  |  |  | |
| 1. Ability to identify unintentional weight change that may relate to disease status and recommend that the client seek appropriate medical attention. | ACSM |  |  |  | |
| 1. Knowledge of effect of chemotherapy and radiation on the mouth and gastrointestinal system, and the result of these changes on appetite, and food preferences and choices. | ACSM |  |  |  | |
| 1. **Ability to discern when a participant's nutritional questions or status would be best managed by referral to a registered dietitian.** | ACSM |  |  | Frequency  Rarely = 0%  Infreq = 21%  Freq = 42%  Very Freq = 37%  Not sure = 0% | Mastery  Adv beg = 12%  Comp = 21%  Proficient = 46%  Expert = 21%  Not sure = 0% |
| 1. Knowledge of current nutrition guidelines during and after cancer treatment. | ACSM |  |  |  | |
| 1. Knowledge of hydration needs specific to cancer patients and survivors. | ACSM |  |  |  | |
| 1. Knowledge of safety of weight loss programs for cancer survivors. | ACSM |  |  |  | |
| 1. Understanding of the general guidelines of nutrition and how they are related to sarcopenia. | Participant addition | N/A |  |  | |
| 1. Sarcopenic impact of body composition changes and their impact on lean body mass and weight gain. | Participant addition | N/A |  |  | |
| 1. Ability to administer an appropriate nutritional screening. | Participant addition | N/A |  |  | |
| 1. **Know when and how to refer to and collaborate with Registered Dieticians.** | Participant addition | N/A | 88% = very important or absolutely essential  8% = of average importance  4% = of little importance | Frequency  Rarely = 0%  Infreq = 38%  Freq = 42%  Very Freq = 21%  Not sure = 0% | Mastery  Adv beg = 12%  Comp = 29%  Proficient = 46%  Expert = 13%  Not sure = 0% |

| Category 5: Human behavior and counseling (93%) |  |  |  |  | |
| --- | --- | --- | --- | --- | --- |
| **Specific Knowledge, Skill, Ability (KSA) for evaluation** | **Source** | **Round 1 (n=29)** | **Round 2 (n=25)** | **Round 3 (n=24)** | |
| 1. **Knowledge to identify a teachable moment for cancer survivors and ability to use that time to provide appropriate information and education about resuming or adopting an exercise program.** | ACSM |  |  | Frequency  Rarely = 0%  Infreq = 8%  Freq = 54%  Very Freq = 38%  Not sure = 0% | Mastery  Adv beg = 0%  Comp = 25%  Proficient = 38%  Expert = 38%  Not sure = 0% |
| 1. **General knowledge of psycho-social problems common to cancer survivors, such as depression, anxiety, fear of recurrence, sleep disturbances, body image, sexual dysfunction, and work and marital difficulties.** | ACSM | 88% = very important or absolutely essential  14% = of average importance |  | Frequency  Rarely = 0%  Infreq = 4%  Freq = 54%  Very Freq = 42%  Not sure = 0% | Mastery  Adv beg = 8%  Comp = 12%  Proficient = 54%  Expert = 25%  Not sure = 0% |
| 1. **Knowledge of behavioral strategies that can enhance motivation and adherence (e.g. goal setting, exercise logs, planning).** | ACSM + CanRehab |  |  | Frequency  Rarely = 0%  Infreq = 4%  Freq = 54%  Very Freq = 42%  Not sure = 0% | Mastery  Adv beg = 8%  Comp = 17%  Proficient = 46%  Expert = 29%  Not sure = 0% |
| 1. **Knowledge of the impact of cancer diagnosis and treatment on quality of life (QOL), and the potential for exercise to enhance a range of QOL outcomes for survivors (e.g. sleep, fatigue, and other factors).** | ACSM |  |  | Frequency  Rarely = 0%  Infreq = 0%  Freq = 42%  Very Freq = 58%  Not sure = 0% | Mastery  Adv beg = 4%  Comp = 21%  Proficient = 33%  Expert = 38%  Not sure = 0%  Missing = 4% |
| 1. Knowledge of and ability to determine effectiveness of group exercise programming vs. individual exercise to meet client's needs. | ACSM | 66% = very important or absolutely essential  34% = of average importance |  |  | |
| 1. **Knowledge of how cancer and cancer treatment relate to ability and readiness to start an exercise program.** | ACSM |  |  | Frequency  Rarely = 0%  Infreq = 4%  Freq = 50%  Very Freq = 42%  Not sure = 4% | Mastery  Adv beg = 0%  Comp = 21%  Proficient = 46%  Expert = 29%  Not sure = 4% |
| 1. Ability to facilitate the social support needs that are cancer specific including connections to websites and local support groups. | ACSM | 66% = very important or absolutely essential  34% = of average importance |  |  | |
| 1. **Demonstrate communication skills and compassion for patients/clients who have suffered the physical and psychological trauma of cancer and its management.** | CanRehab |  |  | Frequency  Rarely = 0%  Infreq = 0%  Freq = 33%  Very Freq = 67%  Not sure = 0% | Mastery  Adv beg = 4%  Comp = 17%  Proficient = 50%  Expert = 29%  Not sure = 0% |
| 1. **Understand the patient’s goals for exercise and know how to use them to set realistic expectations for exercise.** | Participant addition | N/A | 88% = very important or absolutely essential  8% = of average importance  4% = of little importance | Frequency  Rarely = 0%  Infreq = 4%  Freq = 46%  Very Freq = 50%  Not sure = 0% | Mastery  Adv beg = 8%  Comp = 17%  Proficient = 50%  Expert = 25%  Not sure = 0% |
| 1. Knowledge of health behavior change strategies to help patients be as physically active as possible. Three specific health behavior techniques were raised: 2. Motivational Interviewing | Participant addition | N/A |  |  | |
| 11. Knowledge of health behavior change strategies to help patients be as physically active as possible. Three specific health behavior techniques were raised:   1. Health coaching | Participant addition | N/A |  |  | |
| 12. Knowledge of health behavior change strategies to help patients be as physically active as possible. Three specific health behavior techniques were raised:   1. Cognitive Behavioral Therapy | Participant addition | N/A |  |  | |
| 13. Understand who is part of a patient’s support system. | Participant addition | N/A |  |  | |
| **14. Demonstrate an understanding of the patient’s personal circumstances, needs, and concerns relating to their cancer treatment.** | Participant addition | N/A | 84% = very important or absolutely essential  12% = of average importance  4% = of little importance | Frequency  Rarely = 4%  Infreq = 4%  Freq = 58%  Very Freq = 33%  Not sure = 0% | Mastery  Adv beg = 8%  Comp = 21%  Proficient = 58%  Expert = 8%  Not sure = 4% |
| **15. Understand common barriers to (and facilitators of) exercise and be able to work with patient to overcome as many as possible.** | Participant addition | N/A | 88% = very important or absolutely essential  8% = of average importance  4% = of little importance | Frequency  Rarely = 0%  Infreq = 0%  Freq = 50%  Very Freq = 50%  Not sure = 0% | Mastery  Adv beg = 4%  Comp = 21%  Proficient = 46%  Expert = 29%  Not sure = 0% |

| Category 6: Safety, injury prevention, and emergency procedures (97%) | | | | | |
| --- | --- | --- | --- | --- | --- |
| **Specific Knowledge, Skill, Ability (KSA) for evaluation** | **Source** | **Round 1 (n=29)** | **Round 2 (n=25)** | **Round 3 (n=24)** | |
| 1. **Knowledge of and ability to recognize and respond to cancer-specific safety issues, such as: susceptibility to infection, musculoskeletal and orthopedic changes, unilateral edema, fatigue, lymphedema, neurological changes, osteoporosis, cognitive decline associated with treatment.** | ACSM + CanRehab |  |  | Frequency  Rarely = 4%  Infreq = 4%  Freq = 50%  Very Freq = 42%  Not sure = 0% | Mastery  Adv beg = 0%  Comp = 4%  Proficient = 58%  Expert = 38%  Not sure = 0% |
| 1. **Knowledge of and ability to respond to cancer specific emergencies, including: sudden loss of limb function, fever in immune-incompetent patient, and mental status changes.** | ACSM + CanRehab |  |  | Frequency  Rarely = 33%  Infreq = 17%  Freq = 38%  Very Freq = 13%  Not sure = 0% | Mastery  Adv beg = 4%  Comp = 17%  Proficient = 33%  Expert = 46%  Not sure = 0% |
| 1. **Knowledge of and ability to respond to the signs and symptoms of new onset and major life threatening complications of cancer, such as superior vena cava syndrome (SVCS), sepsis or infection, and spinal cord compression.** | ACSM + CanRehab |  |  | Frequency  Rarely = 50%  Infreq = 38%  Freq = 4%  Very Freq = 8%  Not sure = 0% | Mastery  Adv beg = 4%  Comp = 17%  Proficient = 17%  Expert = 63%  Not sure = 0% |
| 1. **Knowledge of and ability to write-up incident documentation related to cancer specific adverse events.** | ACSM |  |  | Frequency  Rarely = 54%  Infreq = 29%  Freq = 13%  Very Freq = 4%  Not sure = 0% | Mastery  Adv beg = 0%  Comp = 25%  Proficient = 50%  Expert = 25%  Not sure = 0% |

| Category 7: Program administration, quality assurance, and outcome assessment (93%) | | | | | |
| --- | --- | --- | --- | --- | --- |
| **Specific Knowledge, Skill, Ability (KSA) for evaluation** | **Source** | **Round 1 (n=29)** | **Round 2 (n=25)** | **Round 3 (n=24)** | |
| 1. Knowledge of role in administration and program management within a cancer center, cancer treatment facility, and outpatient setting. | ACSM |  |  |  | |
| 1. Knowledge of the types of exercise resources and programs available nationally and in the local community and which of these programs cater specifically to the needs of cancer survivors. | ACSM + CanRehab |  |  |  | |
| 1. Knowledge of and ability to implement effective, professional business practices and ethical promotion of personal training services to the cancer care community (e.g. physicians, nurses, social workers, physical therapists, survivors and their families). | ACSM |  |  |  | |
| 1. Knowledge of the patient privacy standards and ability to implement systems to ensure confidentiality of cancer related protected health information of participants. | ACSM |  |  |  | |
| 1. Knowledge and ability to obtain referral from physician and communicate with physician about adverse events, abilities and limitations of survivor, and outcomes of testing and training. | ACSM |  |  |  | |
| 1. Ability to recommend appropriate websites and refer to other health professionals. | ACSM |  |  |  | |
| 1. Knowledge of reimbursement programs as eligible/available. | ACSM |  |  |  | |
| 1. Relevant medical/legal issues. | CanRehab |  |  |  | |
| 1. **How to establish a safe and stimulating activity environment sensitive to the physical and psychological, confidentially needs of patients/clients with cancer including the appropriateness of group or individual therapies.** | CanRehab |  |  | Frequency  Rarely = 4%  Infreq = 4%  Freq = 54%  Very Freq = 38%  Not sure = 0% | Mastery  Adv beg = 8%  Comp = 17%  Proficient = 42%  Expert = 29%  Not sure = 0%  Missing = 4% |
| 1. The management, evaluation and reporting of information, in verbal and written formats. | CanRehab |  |  |  | |
| 1. **Select appropriate objective outcome measures to address needs raised patient history, including Patient Related Outcome Measures (PROMS) and quality of life assessments.** | Participant addition | N/A | 84% = very important or absolutely essential  16% = of average importance | Frequency  Rarely = 0%  Infreq = 25%  Freq = 42%  Very Freq = 25%  Not sure = 4%  Missing = 4% | Mastery  Adv beg = 4%  Comp = 21%  Proficient = 50%  Expert = 17%  Not sure = 4%  Missing = 4% |
| 1. **Establish collaborative working professional relationships with the oncology treatment and cancer rehabilitation teams where possible.** | Participant addition | N/A | 84% = very important or absolutely essential  12% = of average importance  4% = of little importance | Frequency  Rarely = 4%  Infreq = 21%  Freq = 54%  Very Freq = 21%  Not sure = 0% | Mastery  Adv beg = 0%  Comp = 12%  Proficient = 50%  Expert = 38%  Not sure = 0% |
| 1. **Understand your role as part of a multidisciplinary care team.** | Participant addition | N/A | 80% = very important or absolutely essential  16% = of average importance  4% = of little importance | Frequency  Rarely = 0%  Infreq = 21%  Freq = 33%  Very Freq = 46%  Not sure = 0% | Mastery  Adv beg = 0%  Comp = 21%  Proficient = 50%  Expert = 29%  Not sure = 0% |

| Category 8: Clinical and medical considerations (96%) |  |  |  |  | |
| --- | --- | --- | --- | --- | --- |
| **Specific Knowledge, Skill, Ability (KSA) for evaluation** | **Source** | **Round 1**  **(n=28; missing = 1)** | **Round 2 (n=25)** | **Round 3 (n=24)** | |
| 1. **Knowledge of the major long-term effects among childhood cancer survivors that may require careful screening and program adaptation for these individuals.** | ACSM | 86% = very important or absolutely essential  11% = of average importance  3% = not sure |  | Frequency  Rarely = 33%  Infreq = 38%  Freq = 25%  Very Freq = 4%  Not sure = 0% | Mastery  Adv beg = 0%  Comp = 17%  Proficient = 50%  Expert = 33%  Not sure = 0% |
| 1. **Knowledge of the common side effects and symptoms of typical cancer treatments (surgeries, chemotherapy, radiation, hormone manipulations, other drugs).** | ACSM + CanRehab |  |  | Frequency  Rarely = 0%  Infreq = 0%  Freq = 25%  Very Freq = 75%  Not sure = 0% | Mastery  Adv beg = 4%  Comp = 12%  Proficient = 38%  Expert = 46%  Not sure = 0% |
| 1. **Knowledge that cancer treatment may accelerate functional decline associated with aging, particularly in the elderly, and that exercise programming may need to be adjusted accordingly.** | ACSM |  |  | Frequency  Rarely = 0%  Infreq = 8%  Freq = 42%  Very Freq = 50%  Not sure = 0% | Mastery  Adv beg = 0%  Comp = 21%  Proficient = 46%  Expert = 33%  Not sure = 0% |
| 1. **Knowledge of the combined effects of aging and cancer-treatment on exercise capacity and selection of appropriate testing modalities and interpretation of results.** | ACSM |  |  | Frequency  Rarely = 0%  Infreq = 4%  Freq = 62%  Very Freq = 29%  Not sure = 0%  Missing = 4% | Mastery  Adv beg = 0%  Comp = 17%  Proficient = 37%  Expert = 46%  Not sure = 0% |
| 1. **Knowledge of the common sites of metastases and ability to design and implement appropriate exercise programs consistent with this knowledge.** | ACSM |  |  | Frequency  Rarely = 0%  Infreq = 25%  Freq = 46%  Very Freq = 29%  Not sure = 0% | Mastery  Adv beg = 0%  Comp = 12%  Proficient = 33%  Expert = 54%  Not sure = 0% |
| 1. **Knowledge of the signs and symptoms associated with new onset lymphedema, and the major cancer types associated with increased lymphedema risk (e.g. breast, head and neck cancer).** | ACSM |  |  | Frequency  Rarely = 0%  Infreq = 33%  Freq = 42%  Very Freq = 25%  Not sure = 0% | Mastery  Adv beg = 4%  Comp = 8%  Proficient = 46%  Expert = 42%  Not sure = 0% |
| 1. **Knowledge of lymphedema risk reduction practices, and exercise guidelines.** | ACSM |  |  | Frequency  Rarely = 4%  Infreq = 12%  Freq = 58%  Very Freq = 25%  Not sure = 0% | Mastery  Adv beg = 4%  Comp = 17%  Proficient = 38%  Expert = 42%  Not sure = 0% |
| 1. **Knowledge of how cancer treatment may alter cardiovascular risk factors, and inappropriate far responses to exercise testing or training.** | ACSM |  |  | Frequency  Rarely = 8%  Infreq = 4%  Freq = 58%  Very Freq = 29%  Not sure = 0% | Mastery  Adv beg = 4%  Comp = 17%  Proficient = 54%  Expert = 25%  Not sure = 0% |
| 1. **Knowledge of lymphatic, neurological and immune system factors in cancer survivors that may require further evaluation by medical or allied health professionals before participation in physical activity.** | ACSM |  |  | Frequency  Rarely = 8%  Infreq = 21%  Freq = 46%  Very Freq = 25%  Not sure = 0% | Mastery  Adv beg = 0%  Comp = 17%  Proficient = 42%  Expert = 42%  Not sure = 0% |
| 1. **Knowledge of how common cancer treatments affects the ability of cancer survivors to perform exercise, and how to adjust programs accordingly.** | ACSM |  |  | Frequency  Rarely = 0%  Infreq = 0%  Freq = 42%  Very Freq = 58%  Not sure = 0% | Mastery  Adv beg = 0%  Comp = 8%  Proficient = 50%  Expert = 38%  Not sure = 0%  Missing = 4% |
| 1. **Knowledge of the effect of cancer treatment on balance and mobility and the ability to develop an appropriate exercise program that minimizes fall/injury risk.** | ACSM |  |  | Frequency  Rarely = 0%  Infreq = 4%  Freq = 46%  Very Freq = 46%  Not sure = 4% | Mastery  Adv beg = 4%  Comp = 21%  Proficient = 37%  Expert = 33%  Not sure = 4% |
| 1. **Knowledge and ability to recognize the limits in the scope of practice for exercise professionals in working with cancer survivors with complex medical issues.** | ACSM |  |  | Frequency  Rarely = 0%  Infreq = 8%  Freq = 67%  Very Freq = 25%  Not sure = 0% | Mastery  Adv beg = 0%  Comp = 17%  Proficient = 50%  Expert = 33%  Not sure = 0% |
| 1. The structure of cancer services and the roles of different professionals involved in the care of the patient at the various stages in their management pathway. | CanRehab | 86% = very important or absolutely essential  14% = of average importance |  |  | |
| 1. Ability to include specific discussions on brain metastases and judgment/cognition, as well as balance. | Participant addition | N/A |  |  | |
| 1. **Be familiar with and able to interpret medical information in the context of exercise prescriptions.** | Participant addition | N/A |  | Frequency  Rarely = 0%  Infreq = 8%  Freq = 42%  Very Freq = 46%  Not sure = 4% | Mastery  Adv beg = 0%  Comp = 12%  Proficient = 54%  Expert = 25%  Not sure = 4%  Missing = 4% |
| 1. Understand roles and responsibilities of members of a holistic multidisciplinary care team (e.g., exercise physiologist,physical therapist, occupational therapist, etc.). | Participant addition | N/A |  |  | |
| 1. **Know common cancer pathophysiology, staging, grading, type of cancer (e.g., TNM score and how this impacts exercise prescription and precautions to consider or implement, etc).** | Participant addition | N/A |  | Frequency  Rarely = 4%  Infreq = 4%  Freq = 50%  Very Freq = 42%  Not sure = 0% | Mastery  Adv beg = 4%  Comp = 21%  Proficient = 42%  Expert = 33%  Not sure = 0% |
| 1. **Understand breast reconstruction.** | Participant addition | N/A |  | Frequency  Rarely = 0%  Infreq = 29%  Freq = 54%  Very Freq = 17%  Not sure = 0% | Mastery  Adv beg = 4%  Comp = 25%  Proficient = 38%  Expert = 33%  Not sure = 0% |
| 1. **General tissue healing timeframes, to then apply to exercise prescription post-surgery as core foundational knowledge.** | Participant addition | N/A |  | Frequency  Rarely = 0%  Infreq = 25%  Freq = 54%  Very Freq = 21%  Not sure = 0% | Mastery  Adv beg = 0%  Comp = 21%  Proficient = 54%  Expert = 25%  Not sure = 0% |
| 1. Know when, where, and how to seek guidance if the client is not making expected gains in training. | Participant addition | N/A |  |  | |
| 1. Emotional and psychological impact of reduced activity tolerance, pain, and the existential threat of a cancer diagnosis in many patients’ sense of control and well-being. | Participant addition | N/A | 80% = very important or absolutely essential  12% = of average importance  4% = of little importance  4% = not sure |  | |
| 1. **Understand the symptoms specific to typical presentation of various cancer diagnoses.** | Participant addition | N/A | 88% = very important or absolutely essential  4% = of average importance  4% = of little importance  4% = not sure | Frequency  Rarely = 0%  Infreq = 8%  Freq = 46%  Very Freq = 37%  Not sure = 4%  Missing = 4% | Mastery  Adv beg = 4%  Comp = 17%  Proficient = 50%  Expert = 25%  Not sure = 4% |
| 1. **Ability to identify potential signs of skeletal metastases progression that may warrant further investigation.** | Participant addition | N/A |  | Frequency  Rarely = 29%  Infreq =46%  Freq = 12%  Very Freq = 13%  Not sure = 0% | Mastery  Adv beg = 0%  Comp = 21%  Proficient = 38%  Expert = 42%  Not sure = 0% |
| 1. **Knowledge of the expected effects of treatment and their impact on patients’ ability to exercise (i.e., when patients will feel well or unwell during a treatment cycle).** | Participant addition | N/A |  | Frequency  Rarely = 0%  Infreq = 4%  Freq = 54%  Very Freq = 42%  Not sure = 0% | Mastery  Adv beg = 0%  Comp = 21%  Proficient = 42%  Expert = 37%  Not sure = 0% |

| Category 9: Physiology, diagnosis, and treatment (93%) |  |  |  |  | |
| --- | --- | --- | --- | --- | --- |
| **Specific Knowledge, Skill, Ability (KSA) for evaluation** | **Source** | **Round 1**  **(n=28; missing = 1)** | **Round 2 (n=25)** | **Round 3 (n=23; missing = 1)** | |
| 1. Knowledge of currently accepted screening practices for surveillance of recurrence for common cancers (e.g., mammography, colonoscopy, prostate specific antigen, pap smears). | ACSM |  |  |  | |
| 1. Knowledge of the pathology tests used to diagnose common cancers (e.g. biopsy, imaging technologies, and blood tests for tumor markers). | ACSM |  |  |  | |
| 1. Knowledge of how to communicate effectively with the major medical specialties with whom cancer survivors may interact, including surgery, medical oncology, radiology, dietitians, and psychologists/psychiatrists. | ACSM + CanRehab |  |  |  | |
| 1. **Knowledge of the most common warning signs of recurrence for common cancers, and when to recommend that clients seek additional medical evaluation.** | ACSM |  |  | Frequency  Rarely = 26%  Infreq = 39%  Freq = 30%  Very Freq = 4%  Not sure = 0% | Mastery  Adv beg = 4%  Comp = 22%  Proficient = 30%  Expert = 43%  Not sure = 0% |
| 1. Understand typical durations of cancer therapy for the major cancers (breast, prostate, melanoma, ovary, lung, colon), and that therapies are continually evolving/changing. | ACSM |  |  |  | |
| 1. **General knowledge of current cancer treatment strategies, including surgery, systemic therapies (e.g. chemotherapy) and targeted therapies (e.g, anti-angiogenesis inhibitors).** | ACSM + CanRehab |  |  | Frequency  Rarely = 0%  Infreq = 9%  Freq = 39%  Very Freq = 52%  Not sure = 0% | Mastery  Adv beg = 0%  Comp = 26%  Proficient = 34%  Expert = 39%  Not sure = 0% |
| 1. **Knowledge of how lifestyle factors, including nutrition, physical activity, and heredity, influence hypothesized mechanisms of cancer etiology, reduce the risk of relapse after initial treatments, and improve long-term survival.** | ACSM + CanRehab |  |  | Frequency  Rarely = 4%  Infreq = 9%  Freq = 48%  Very Freq = 35%  Not sure = 4% | Mastery  Adv beg = 4%  Comp = 30%  Proficient = 26%  Expert = 39%  Not sure = 0% |
| 1. General knowledge of the descriptive epidemiology of cancer, including the prevalence, incidence, and survival statistics for the major cancer types. | ACSM |  |  |  | |
| 1. General knowledge of cancer biology (e.g., initiation, promotion/progression, and metastases), particularly for the four most common cancers: lung, breast, colon, and prostate. | ACSM + CanRehab |  |  |  | |
| 1. The environmental/risk factors that can cause cancer and the factors which help our bodies defend against it. | CanRehab |  |  |  | |
| 1. **Understand whether the goal of treatment is curative or palliative and recognize how to support a patient through each scenario.** | Participant addition | N/A | 86% = very important or absolutely essential  4% = of average importance  4% = of little importance  4% = not sure | Frequency  Rarely = 4%  Infreq = 17%  Freq = 43%  Very Freq = 30%  Not sure = 4% | Mastery  Adv beg = 9%  Comp = 13%  Proficient = 39%  Expert = 34%  Not sure = 4% |
| 1. **Be aware of and keep up-to-date with current research and best practice methods in the field.** | Participant addition | N/A |  | Frequency  Rarely = 4%  Infreq = 39%  Freq = 52%  Very Freq = 4%  Not sure = 0% | Mastery  Adv beg = 4%  Comp = 17%  Proficient = 39%  Expert = 39%  Not sure = 0% |
| 1. **Recognize potential side effects of a patient’s medications and potential contraindications for exercise.** | Participant addition | N/A |  | Frequency  Rarely = 4%  Infreq = 9%  Freq = 52%  Very Freq = 35%  Not sure = 0% | Mastery  Adv beg = 0%  Comp = 30%  Proficient = 30%  Expert = 39%  Not sure = 0% |

| Category 10: Personal skills and attributes |  |  |  |  | |
| --- | --- | --- | --- | --- | --- |
| **Specific Knowledge, Skill, Ability (KSA) for evaluation** | **Source** | **Round 1** | **Round 2 (n=25)** | **Round 3 (n=23; missing = 1)** | |
| 1. **Ability to be flexible with programming based on a patient’s needs.** | Participant addition | N/A |  | Frequency  Rarely = 0%  Infreq = 0%  Freq = 30%  Very Freq = 70%  Not sure = 0% | Mastery  Adv beg = 0%  Comp = 17%  Proficient = 35%  Expert = 48%  Not sure = 0% |
| 1. **Verbal and written communication skills necessary to clearly describe programming goals, expectations, and patient progress to both patients and clinicians.** | Participant addition | N/A |  | Frequency  Rarely = 0%  Infreq = 4%  Freq = 17%  Very Freq = 78%  Not sure = 0% | Mastery  Adv beg = 4%  Comp = 17%  Proficient = 35%  Expert = 44%  Not sure = 0% |
| 1. **Ability to empathize with patients.** | Participant addition | N/A |  | Frequency  Rarely = 0%  Infreq = 0%  Freq = 4%  Very Freq = 96%  Not sure = 0% | Mastery  Adv beg = 9%  Comp = 26%  Proficient = 22%  Expert = 44%  Not sure = 0% |
| 1. **Listening skills.** | Participant addition | N/A |  | Frequency  Rarely = 0%  Infreq = 0%  Freq = 0%  Very Freq = 100%  Not sure = 0% | Mastery  Adv beg = 13%  Comp = 21%  Proficient = 26%  Expert = 39%  Not sure = 0% |
| 1. Ability to build a professional network. | Participant addition | N/A |  |  | |
| 1. **Ability to observe patient needs and respond accordingly.** | Participant addition | N/A |  | Frequency  Rarely = 0%  Infreq = 0%  Freq = 0%  Very Freq = 100%  Not sure = 0% | Mastery  Adv beg = 14%  Comp = 13%  Proficient = 23%  Expert = 50%  Not sure = 0% |
| 1. **Ability to manage patient programming in an organized and efficient manner.** | Participant addition | N/A | 88% = very important or absolutely essential  12% = of average importance | Frequency  Rarely = 0%  Infreq = 0%  Freq = 17%  Very Freq = 78%  Not sure = 4% | Mastery  Adv beg = 9%  Comp = 26%  Proficient = 35%  Expert = 26%  Not sure = 4% |
| 1. **Demonstrate patience in approach to a patient’s needs.** | Participant addition | N/A |  | Frequency  Rarely = 0%  Infreq = 0%  Freq = 17%  Very Freq = 83%  Not sure = 0% | Mastery  Adv beg = 13%  Comp = 17%  Proficient = 39%  Expert = 30%  Not sure = 0% |
| 1. **Ability to establish rapport with patients in a therapeutic relationship.** | Participant addition | N/A |  | Frequency  Rarely = 0%  Infreq = 0%  Freq = 9%  Very Freq = 91%  Not sure = 0% | Mastery  Adv beg = 17%  Comp = 13%  Proficient = 26%  Expert = 44%  Not sure = 0% |
| 1. **A positive approach aiming to make exercise as enjoyable as possible for the patient.** | Participant addition | N/A | 88% = very important or absolutely essential  12% = of average importance | Frequency  Rarely = 0%  Infreq = 4%  Freq = 22%  Very Freq = 74%  Not sure = 0% | Mastery  Adv beg = 13%  Comp = 26%  Proficient = 35%  Expert = 26%  Not sure = 0% |
| 1. **Problem solving/critical thinking skills.** | Participant addition | N/A |  | Frequency  Rarely = 0%  Infreq = 0%  Freq = 13%  Very Freq = 87%  Not sure = 0% | Mastery  Adv beg = 4%  Comp = 22%  Proficient = 30%  Expert = 44%  Not sure = 0% |
| 1. **Be willing to accept feedback for programming and professional improvement.** | Participant addition | N/A |  | Frequency  Rarely = 0%  Infreq = 13%  Freq = 43%  Very Freq = 44%  Not sure = 0% | Mastery  Adv beg = 13%  Comp = 22%  Proficient = 35%  Expert = 30%  Not sure = 0% |

Bolded competencies=included; Green box=the round when consensus occurred; Yellow box=competency that was returned to the group to be re-ranked; Red box=consensus was not reached and competency was removed; Infreq=infrequently; Freq=frequently; Adv beg=advanced beginner; Comp=competent
